# Supplementary figures and images for: Molecular mechanism of Afadin substrate recruitment to the receptor phosphatase PTPRK via its pseudophosphatase domain (part 1 of 2)
Source: eLife. 2022 Oct 20;11:e79855. doi: 10.7554/eLife.79855 (PMC9640194; doi:10.7554/eLife.79855)

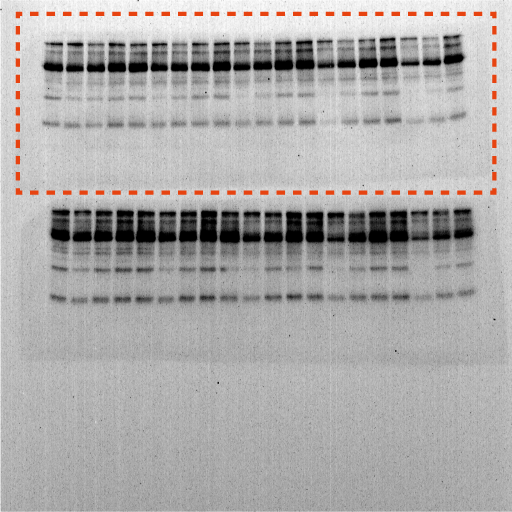

Supplement: Figure 1—source data 1. [file elife-79855-fig1-data1.zip › Figure 1 - source data 1/Original files/Figure 1 - source data 1 - highlighted/Afadin high exposure - highlighted.tif]

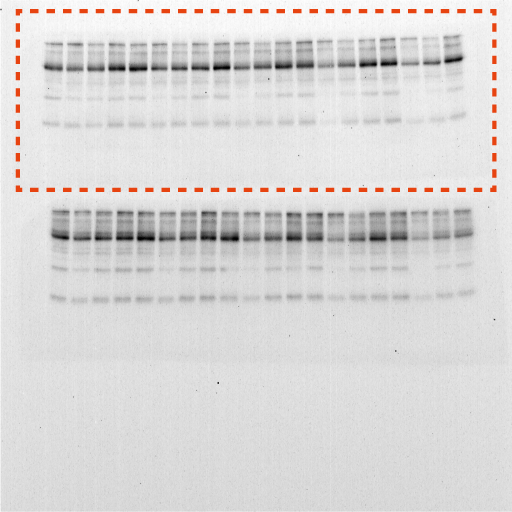

Supplement: Figure 1—source data 1. [file elife-79855-fig1-data1.zip › Figure 1 - source data 1/Original files/Figure 1 - source data 1 - highlighted/Afadin low exposure - highlighted.tif]

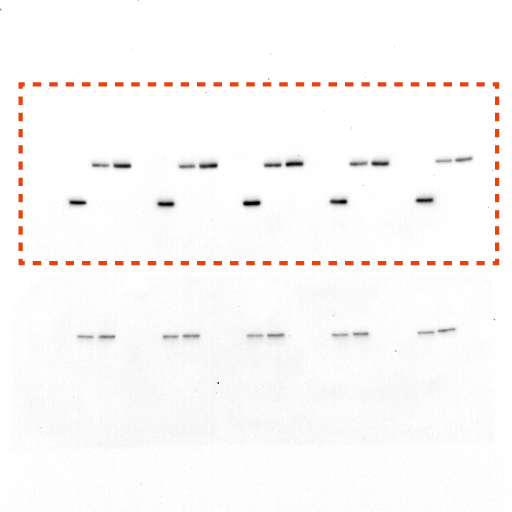

Supplement: Figure 1—source data 1. [file elife-79855-fig1-data1.zip › Figure 1 - source data 1/Original files/Figure 1 - source data 1 - highlighted/His tag - highlighted.tif]

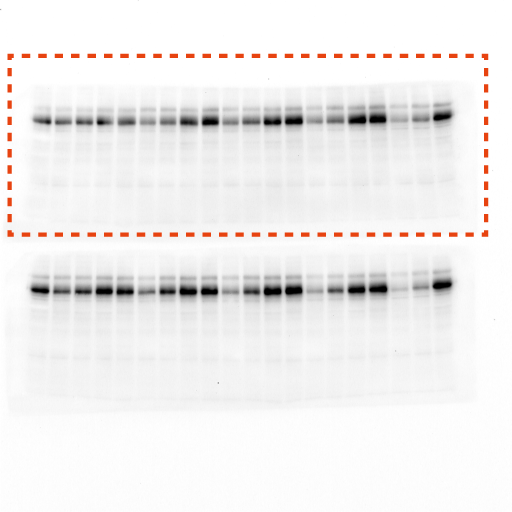

Supplement: Figure 1—source data 1. [file elife-79855-fig1-data1.zip › Figure 1 - source data 1/Original files/Figure 1 - source data 1 - highlighted/p120 Catenin pY228 - highlighted.tif]

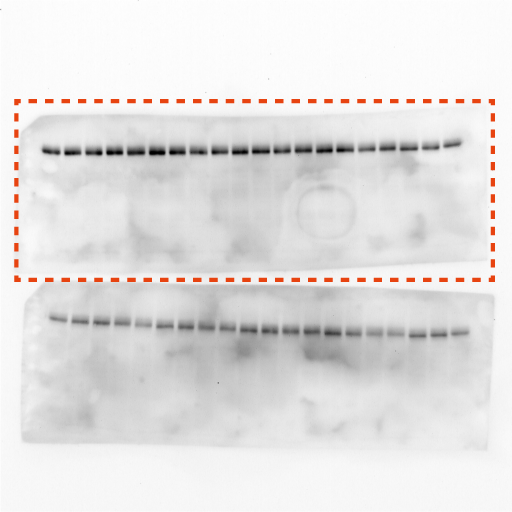

Supplement: Figure 1—source data 1. [file elife-79855-fig1-data1.zip › Figure 1 - source data 1/Original files/Figure 1 - source data 1 - highlighted/p120 total - highlighted.tif]

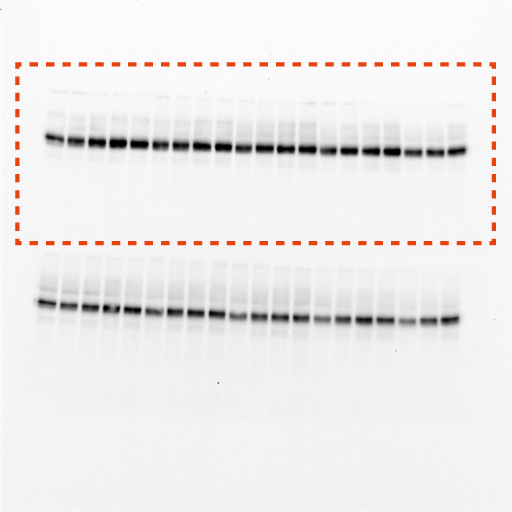

Supplement: Figure 1—source data 1. [file elife-79855-fig1-data1.zip › Figure 1 - source data 1/Original files/Figure 1 - source data 1 - highlighted/Paxillin pY118 - highlighted.tif]

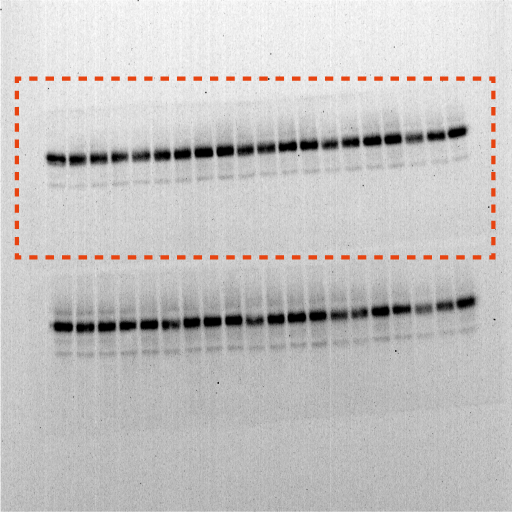

Supplement: Figure 1—source data 1. [file elife-79855-fig1-data1.zip › Figure 1 - source data 1/Original files/Figure 1 - source data 1 - highlighted/Paxillin total - highlighted.tif]

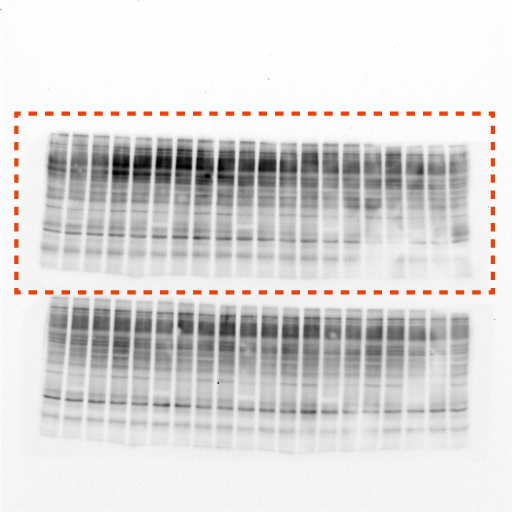

Supplement: Figure 1—source data 1. [file elife-79855-fig1-data1.zip › Figure 1 - source data 1/Original files/Figure 1 - source data 1 - highlighted/pTyr total - highlighted.tif]

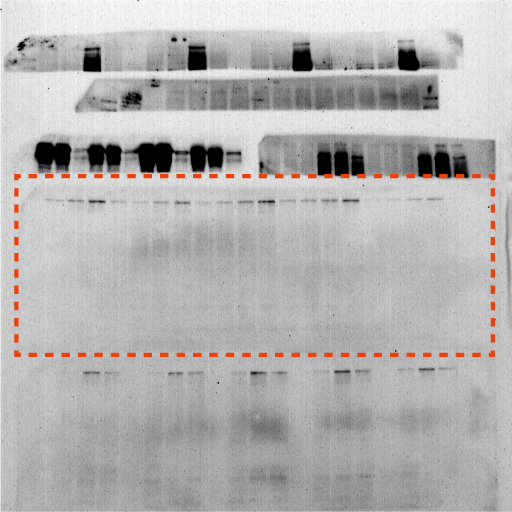

Supplement: Figure 1—source data 1. [file elife-79855-fig1-data1.zip › Figure 1 - source data 1/Original files/Figure 1 - source data 1 - highlighted/Total afadin - highlighted.tif]

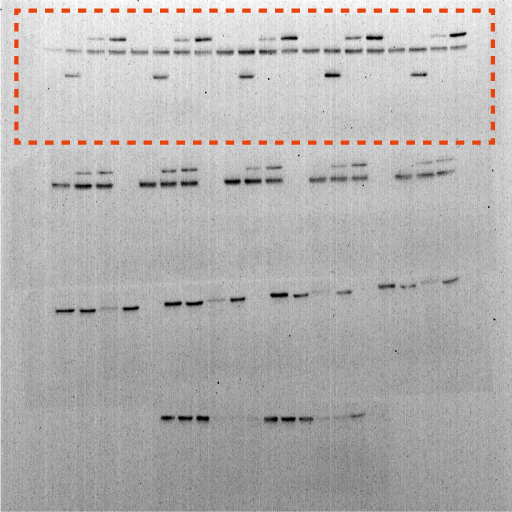

Supplement: Figure 1—source data 1. [file elife-79855-fig1-data1.zip › Figure 1 - source data 1/Original files/Figure 1 - source data 1 - highlighted/Tubulin - highlighted.tif]

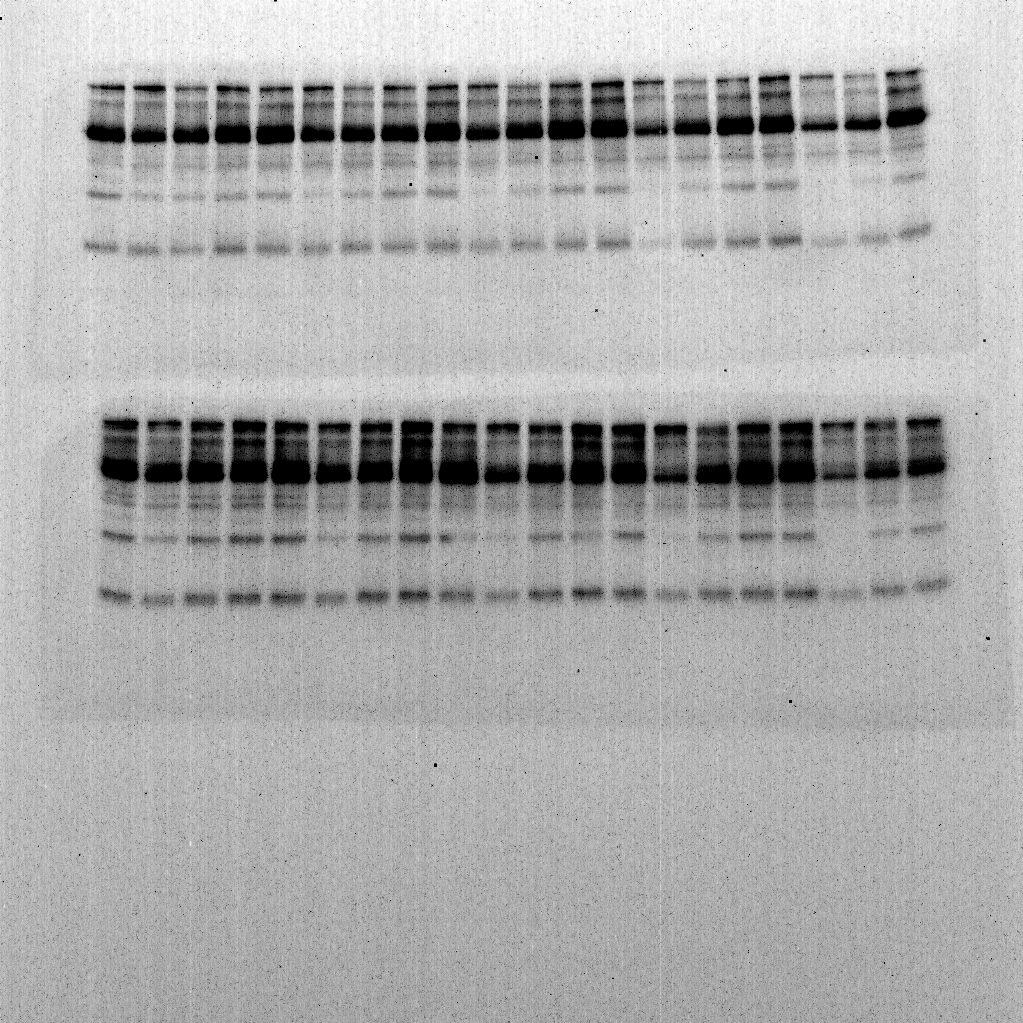

Supplement: Figure 1—source data 1. [file elife-79855-fig1-data1.zip › Figure 1 - source data 1/Original files/Figure 1 source data 1 - unmodified/Afadin high exposure - top blot.tif]

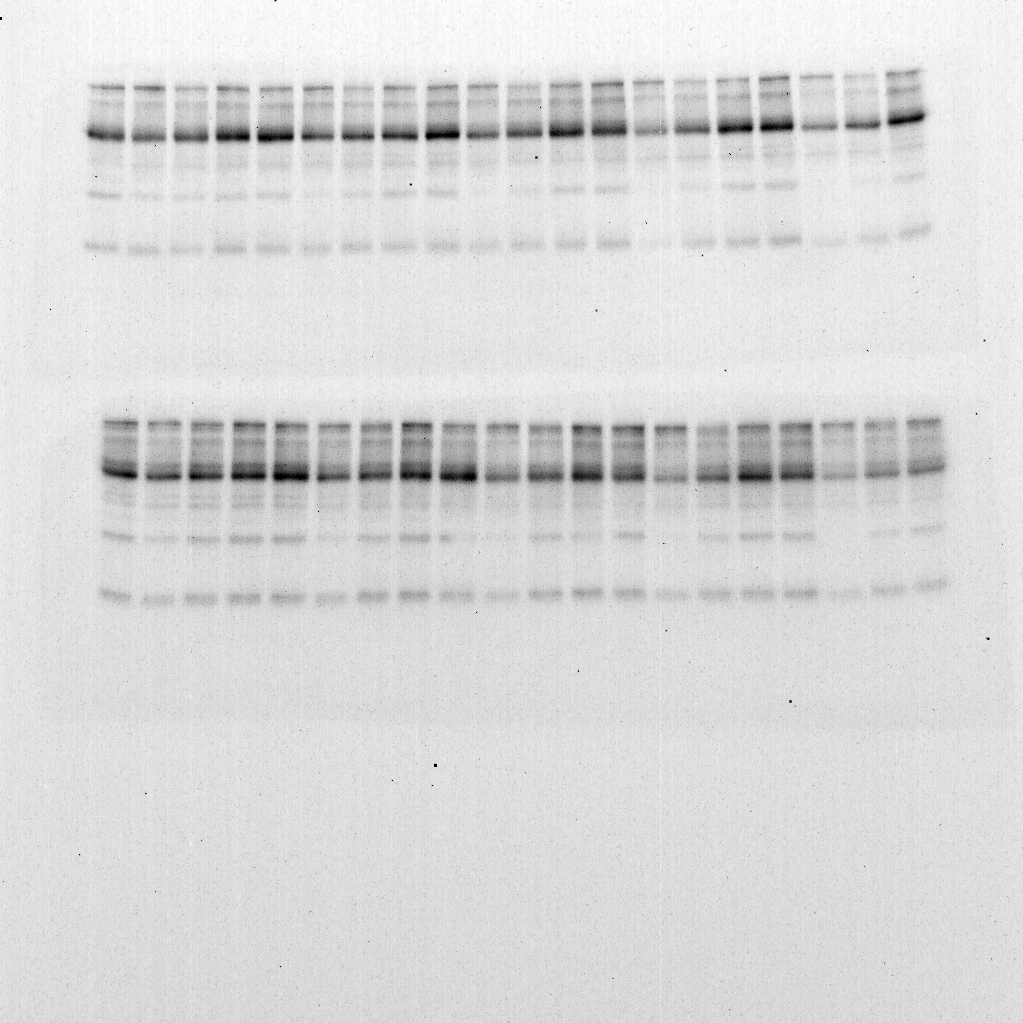

Supplement: Figure 1—source data 1. [file elife-79855-fig1-data1.zip › Figure 1 - source data 1/Original files/Figure 1 source data 1 - unmodified/Afadin low exposure - top blot.tif]

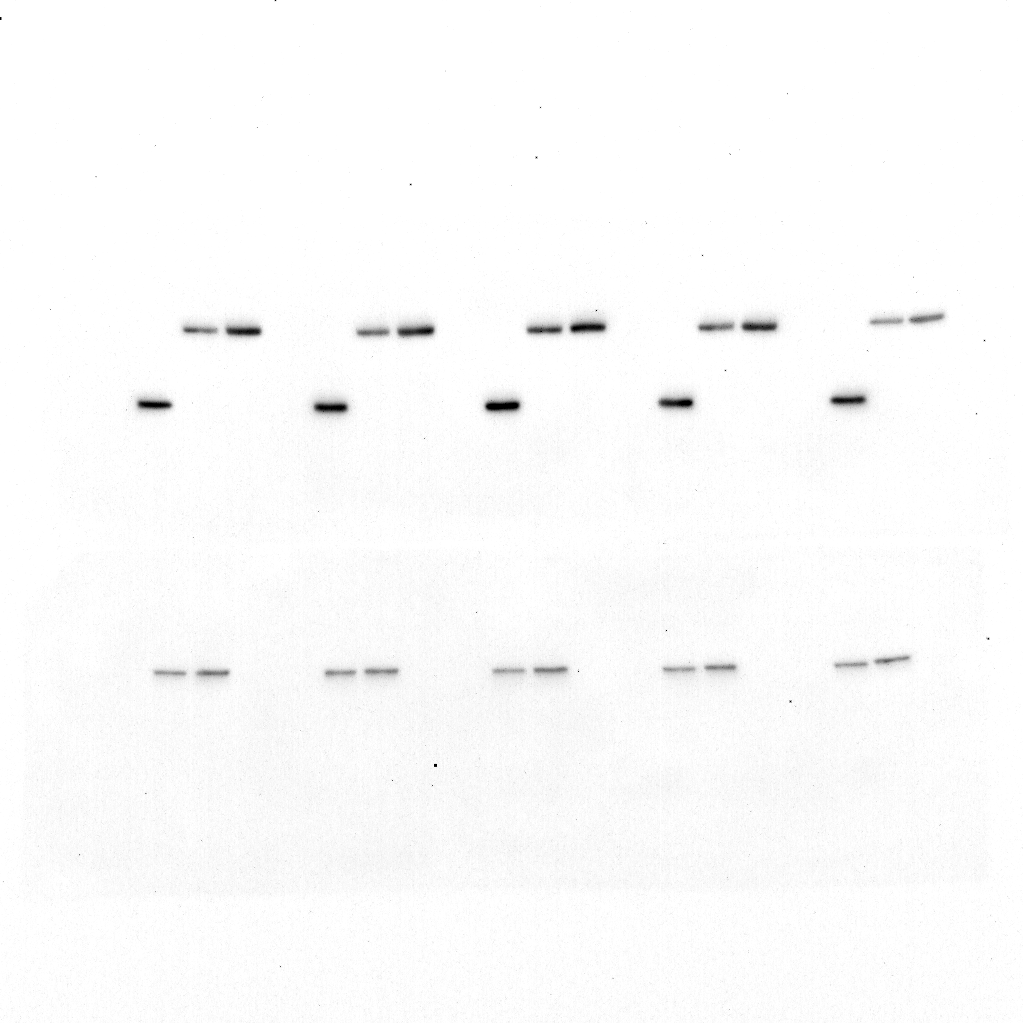

Supplement: Figure 1—source data 1. [file elife-79855-fig1-data1.zip › Figure 1 - source data 1/Original files/Figure 1 source data 1 - unmodified/His tag - top blot.tif]

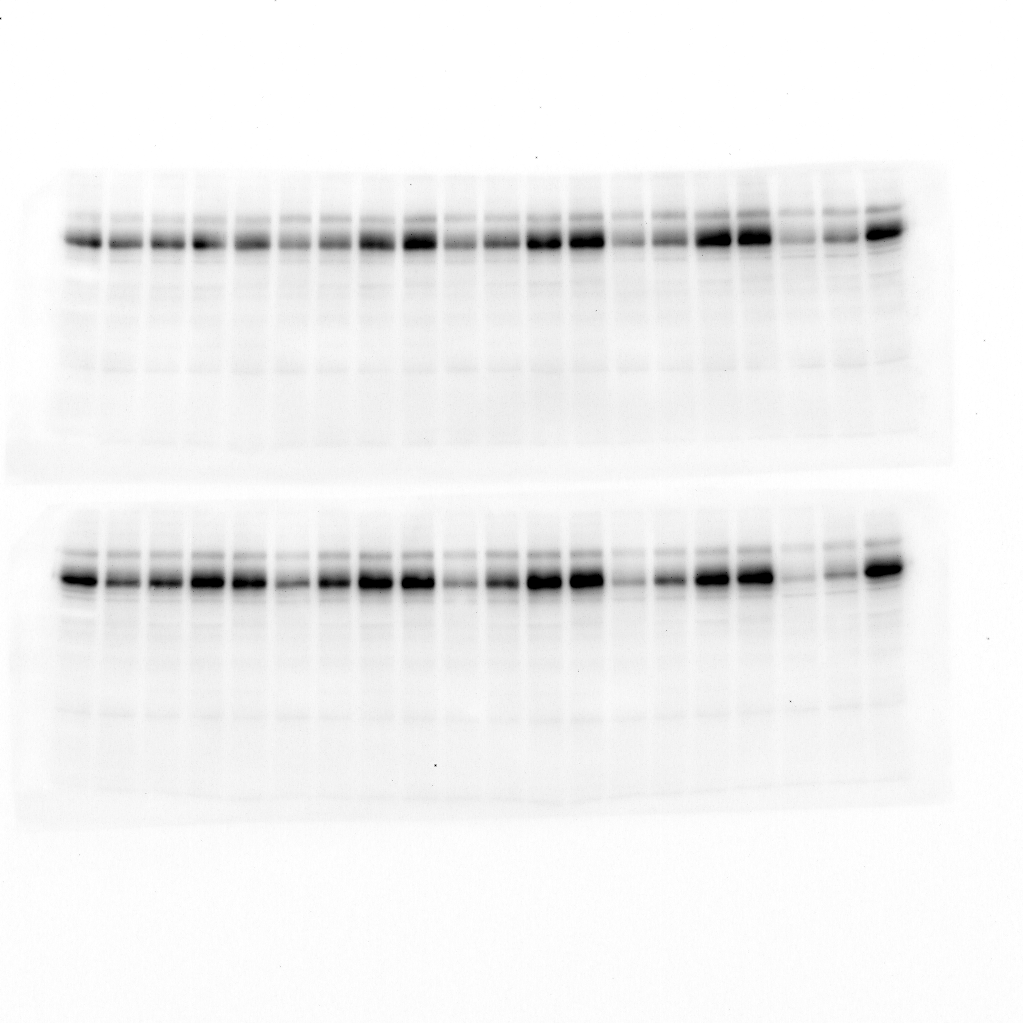

Supplement: Figure 1—source data 1. [file elife-79855-fig1-data1.zip › Figure 1 - source data 1/Original files/Figure 1 source data 1 - unmodified/p120 Catenin pY228 - top blot.tif]

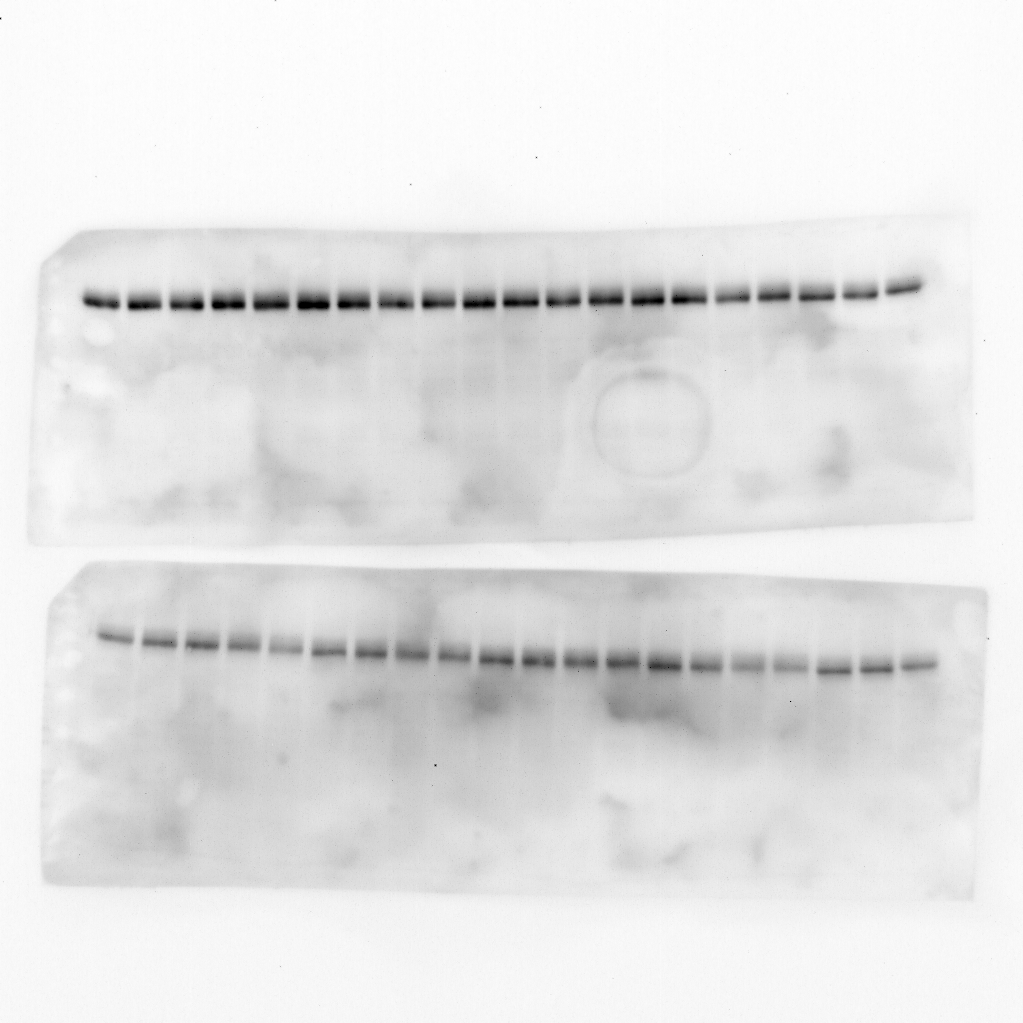

Supplement: Figure 1—source data 1. [file elife-79855-fig1-data1.zip › Figure 1 - source data 1/Original files/Figure 1 source data 1 - unmodified/p120 total - top blot.tif]

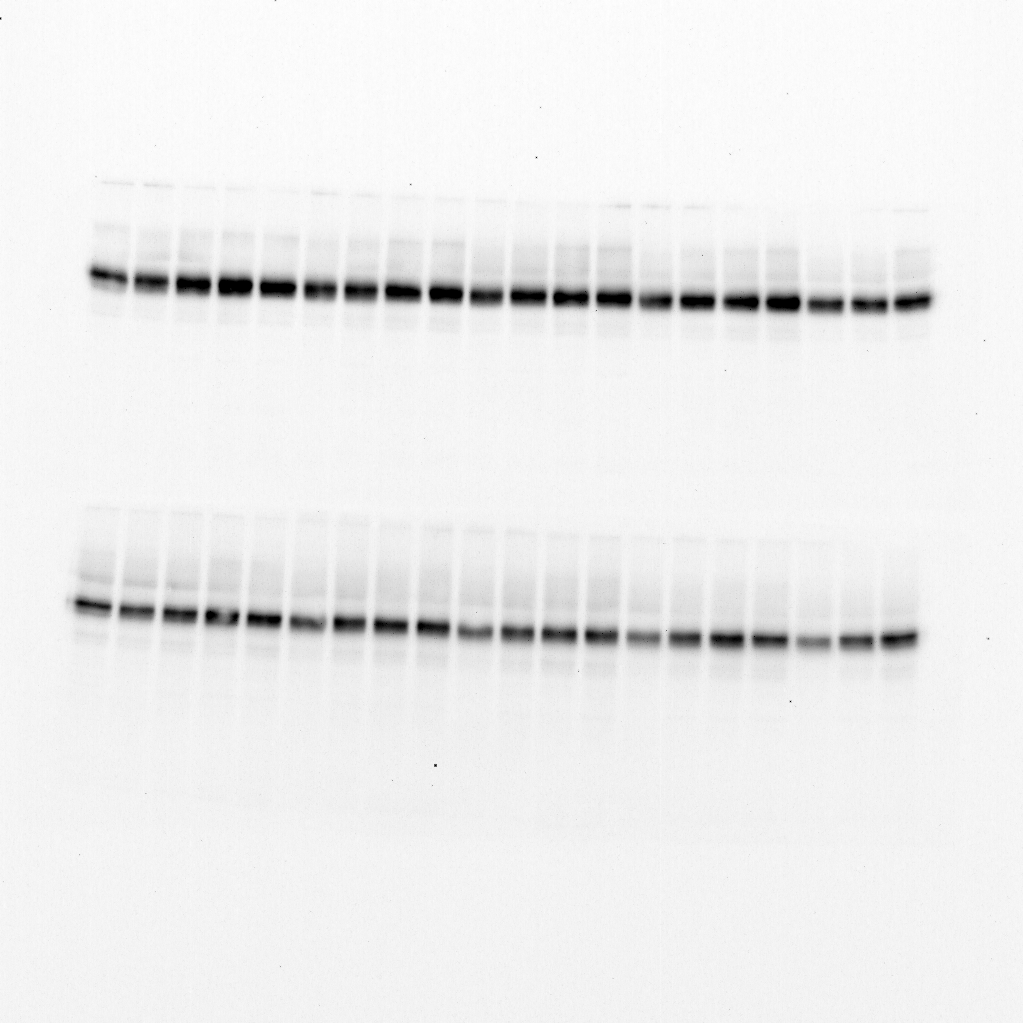

Supplement: Figure 1—source data 1. [file elife-79855-fig1-data1.zip › Figure 1 - source data 1/Original files/Figure 1 source data 1 - unmodified/Paxillin pY118 - top blot.tif]

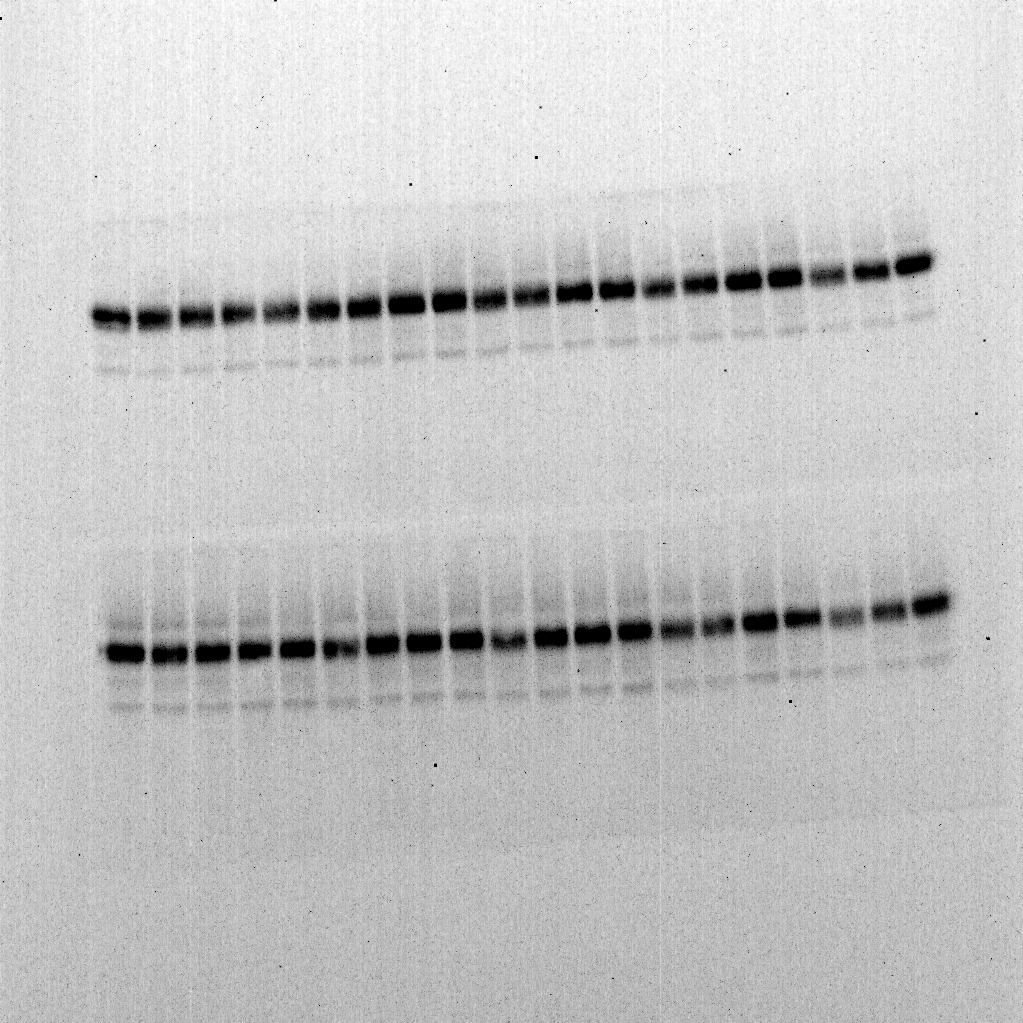

Supplement: Figure 1—source data 1. [file elife-79855-fig1-data1.zip › Figure 1 - source data 1/Original files/Figure 1 source data 1 - unmodified/Paxillin total - top blot.tif]

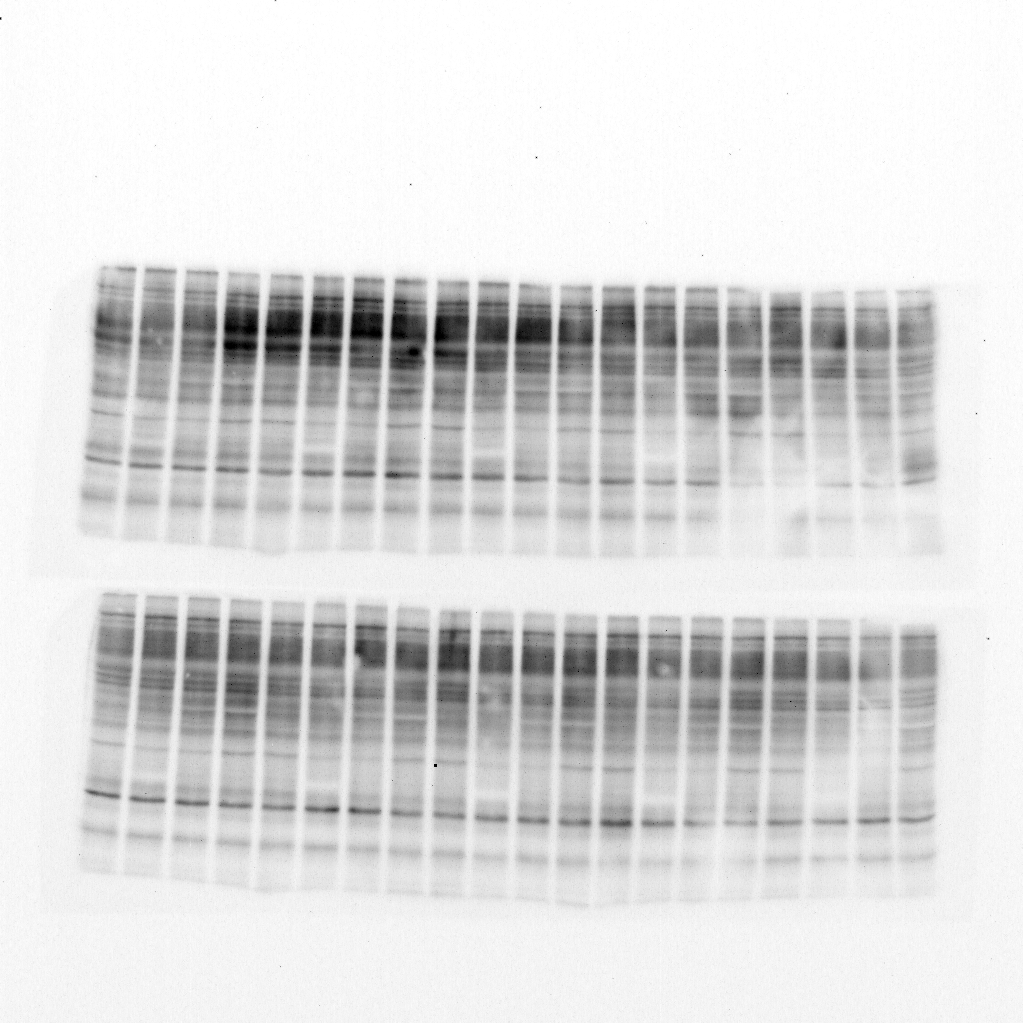

Supplement: Figure 1—source data 1. [file elife-79855-fig1-data1.zip › Figure 1 - source data 1/Original files/Figure 1 source data 1 - unmodified/pTyr total - top blot.tif]

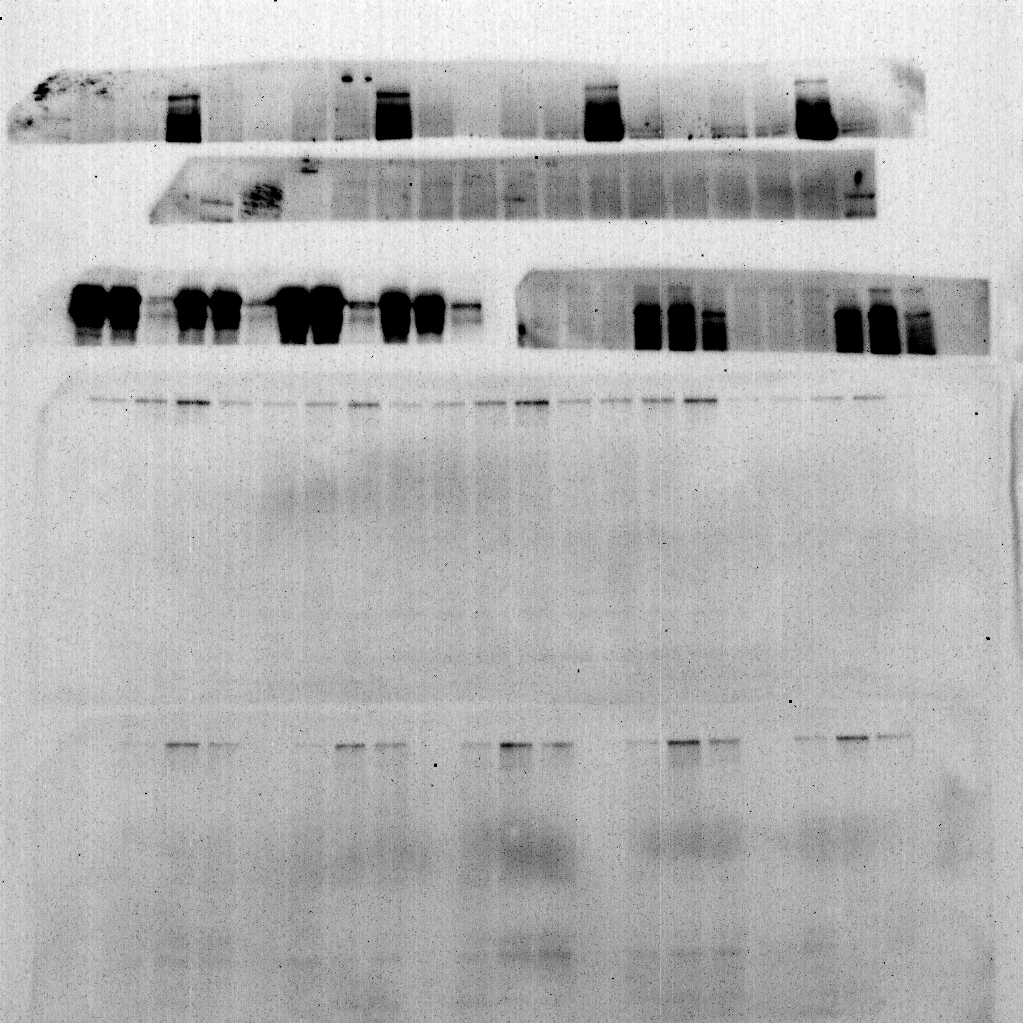

Supplement: Figure 1—source data 1. [file elife-79855-fig1-data1.zip › Figure 1 - source data 1/Original files/Figure 1 source data 1 - unmodified/Total afadin - 4th blot down.tif]

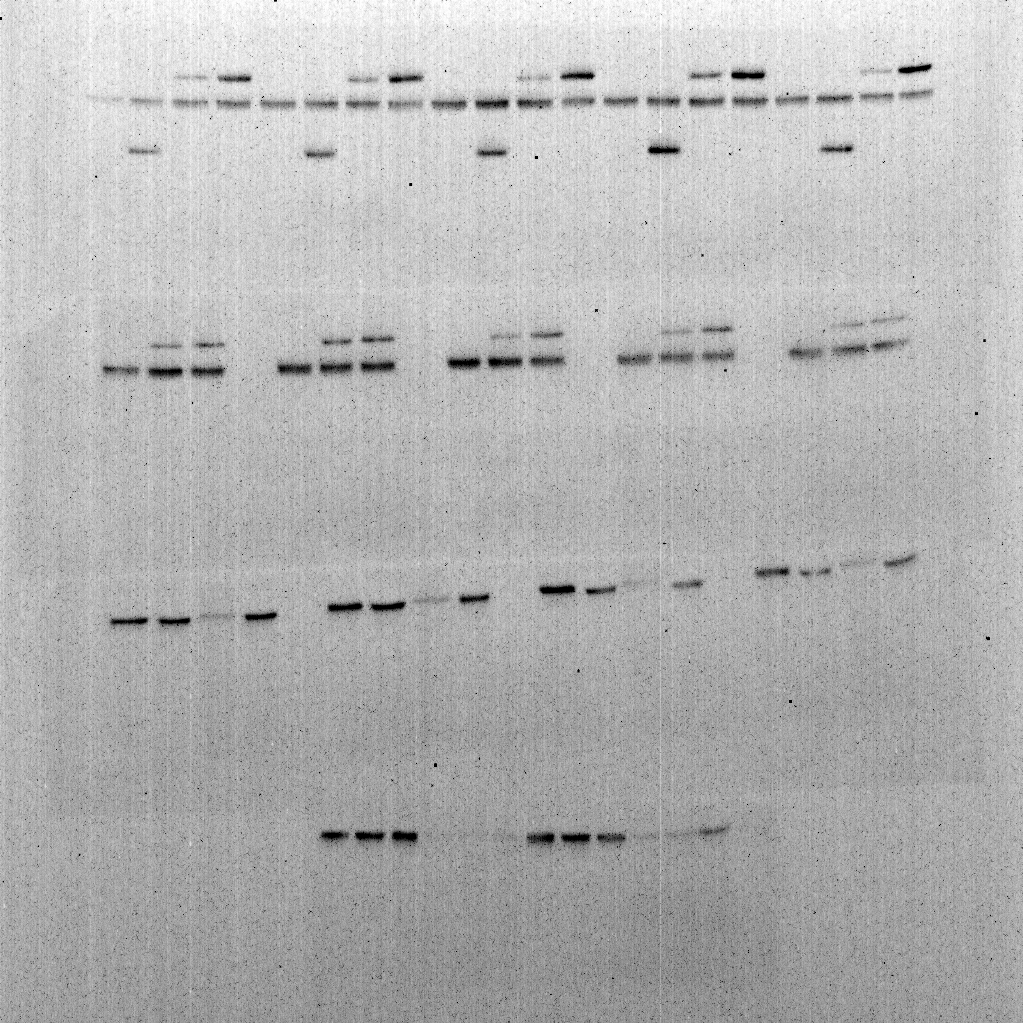

Supplement: Figure 1—source data 1. [file elife-79855-fig1-data1.zip › Figure 1 - source data 1/Original files/Figure 1 source data 1 - unmodified/Tubulin - top blot.tif]

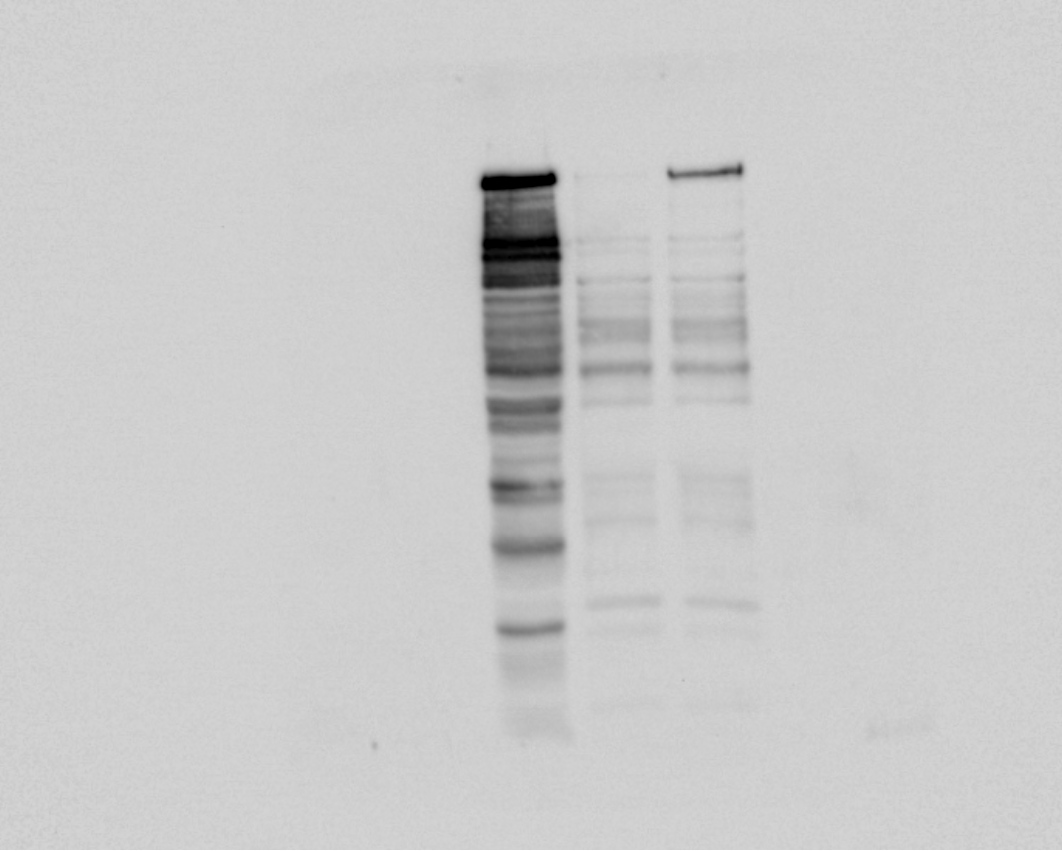

Supplement: Figure 1—source data 2. [file elife-79855-fig1-data2.zip › Figure 1 - source data 2/Original files/1C_Myctag.tif]

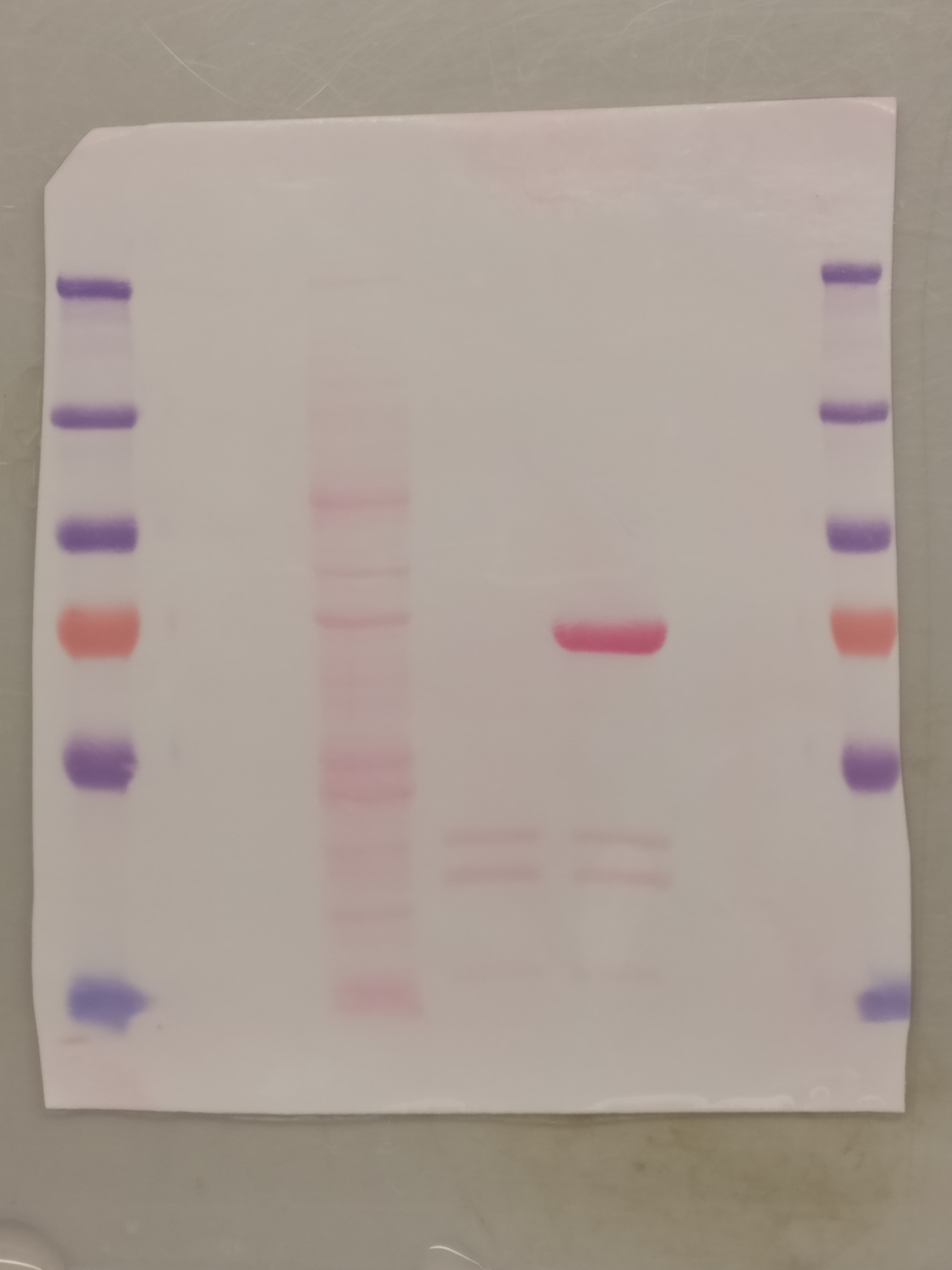

Supplement: Figure 1—source data 2. [file elife-79855-fig1-data2.zip › Figure 1 - source data 2/Original files/1C_ponceau.jpg]

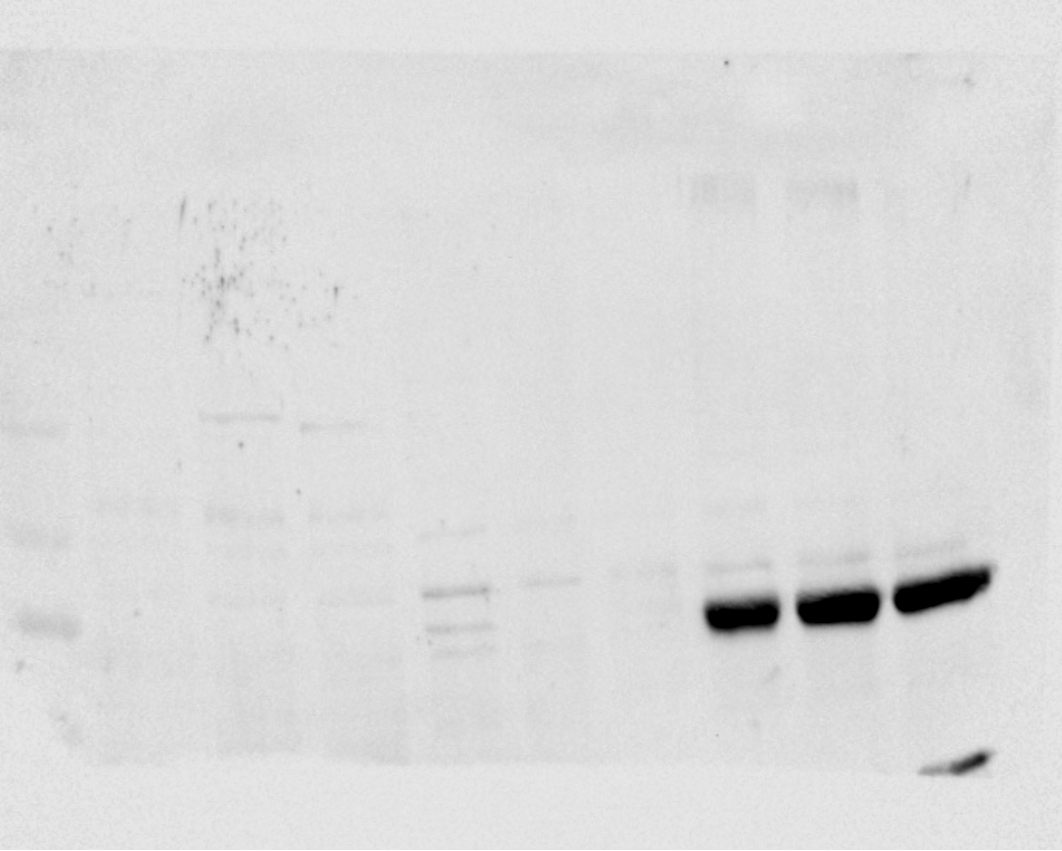

Supplement: Figure 1—source data 3. [file elife-79855-fig1-data3.zip › Figure 1 - source data 3/Original files/1D_Histag.tif]

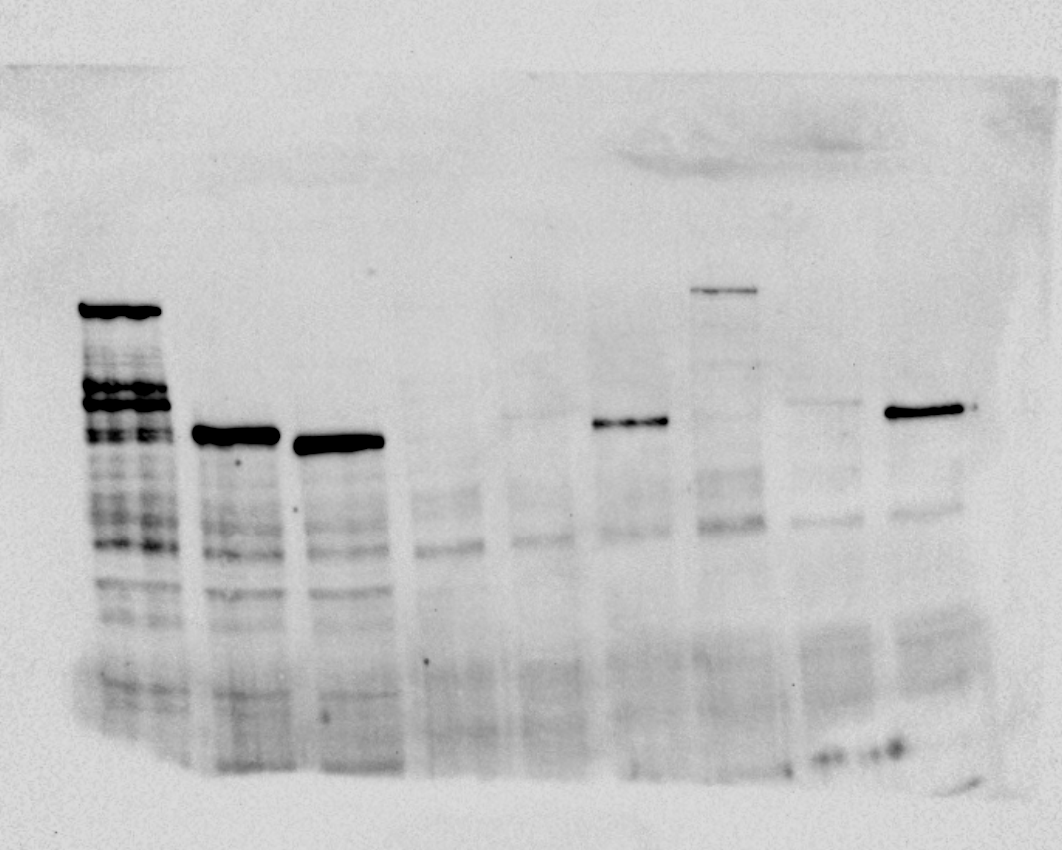

Supplement: Figure 1—source data 3. [file elife-79855-fig1-data3.zip › Figure 1 - source data 3/Original files/1D_Myctag.tif]

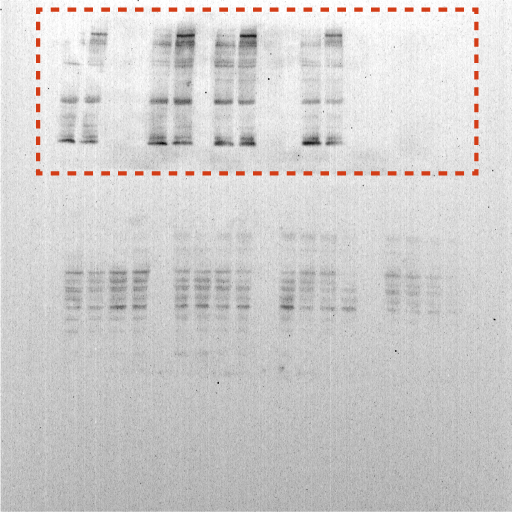

Supplement: Figure 1—figure supplement 2—source data 1. [file elife-79855-fig1-figsupp2-data1.zip › Figure 1 - figure supplement 2 - source data 1/Original files/Figure 1 - figure supplement 2 - source data 1 - highlighted/Afadin pY1230 - highlighted.tif]

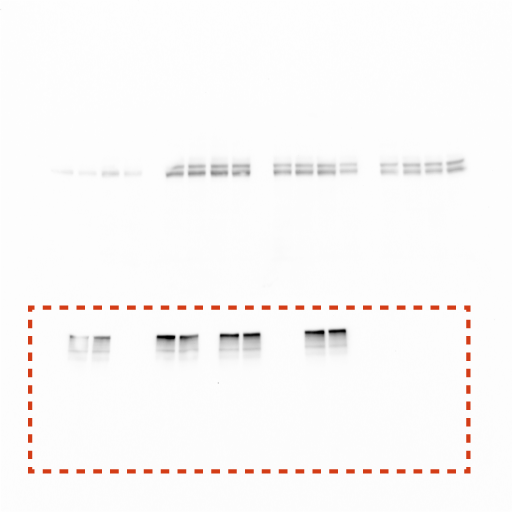

Supplement: Figure 1—figure supplement 2—source data 1. [file elife-79855-fig1-figsupp2-data1.zip › Figure 1 - figure supplement 2 - source data 1/Original files/Figure 1 - figure supplement 2 - source data 1 - highlighted/Afadin total - highlighted.tif]

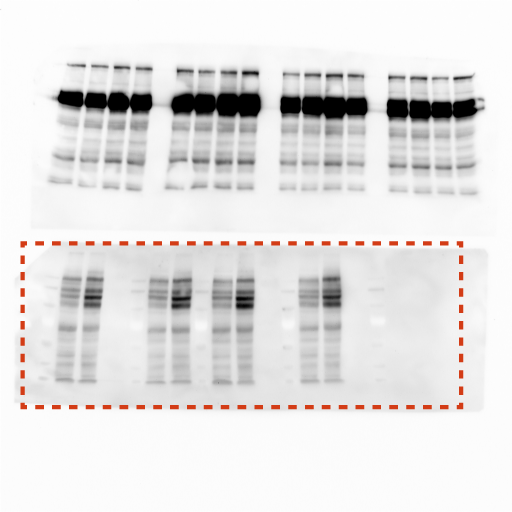

Supplement: Figure 1—figure supplement 2—source data 1. [file elife-79855-fig1-figsupp2-data1.zip › Figure 1 - figure supplement 2 - source data 1/Original files/Figure 1 - figure supplement 2 - source data 1 - highlighted/p120 catenin pY228 - highlighted.tif]

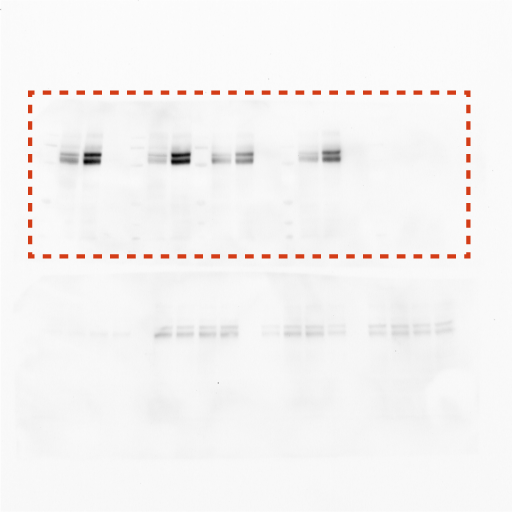

Supplement: Figure 1—figure supplement 2—source data 1. [file elife-79855-fig1-figsupp2-data1.zip › Figure 1 - figure supplement 2 - source data 1/Original files/Figure 1 - figure supplement 2 - source data 1 - highlighted/p120 catenin pY904 - highlighted.tif]

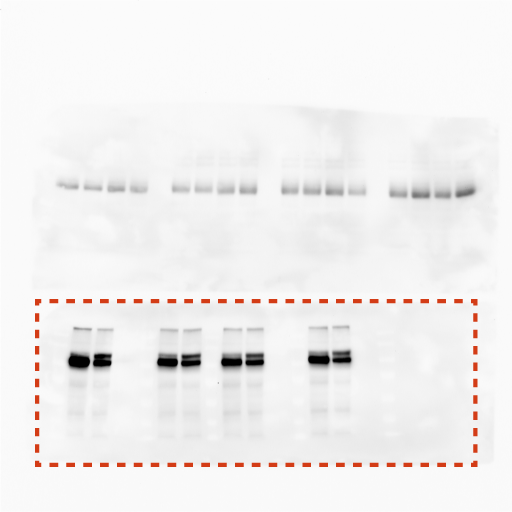

Supplement: Figure 1—figure supplement 2—source data 1. [file elife-79855-fig1-figsupp2-data1.zip › Figure 1 - figure supplement 2 - source data 1/Original files/Figure 1 - figure supplement 2 - source data 1 - highlighted/p120 catenin total - highlighted.tif]

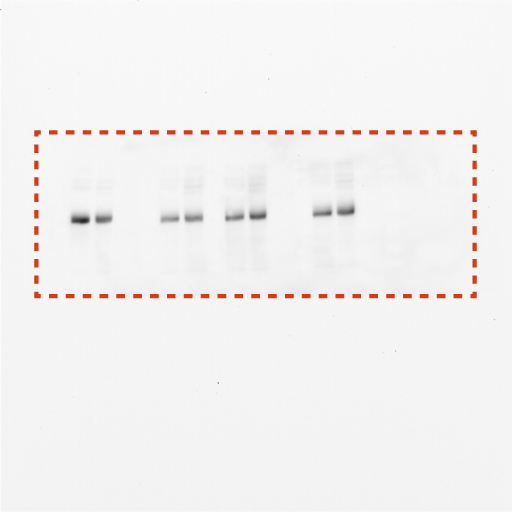

Supplement: Figure 1—figure supplement 2—source data 1. [file elife-79855-fig1-figsupp2-data1.zip › Figure 1 - figure supplement 2 - source data 1/Original files/Figure 1 - figure supplement 2 - source data 1 - highlighted/Paxillin pY118 - highlighted.tif]

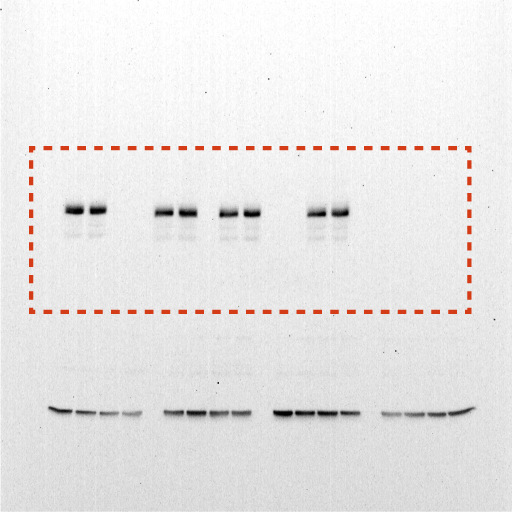

Supplement: Figure 1—figure supplement 2—source data 1. [file elife-79855-fig1-figsupp2-data1.zip › Figure 1 - figure supplement 2 - source data 1/Original files/Figure 1 - figure supplement 2 - source data 1 - highlighted/Paxillin total - highlighted.tif]

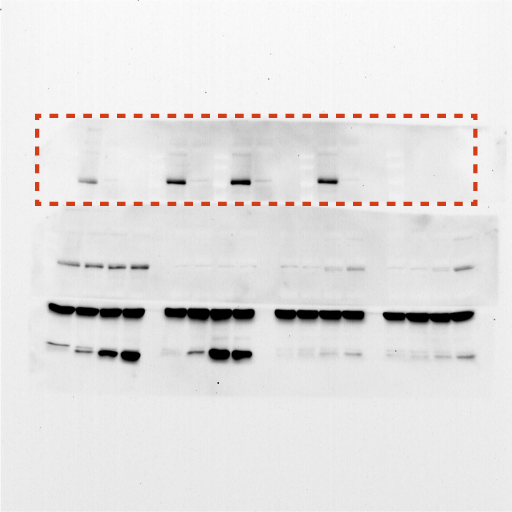

Supplement: Figure 1—figure supplement 2—source data 1. [file elife-79855-fig1-figsupp2-data1.zip › Figure 1 - figure supplement 2 - source data 1/Original files/Figure 1 - figure supplement 2 - source data 1 - highlighted/PTPRK - highlighted.tif]

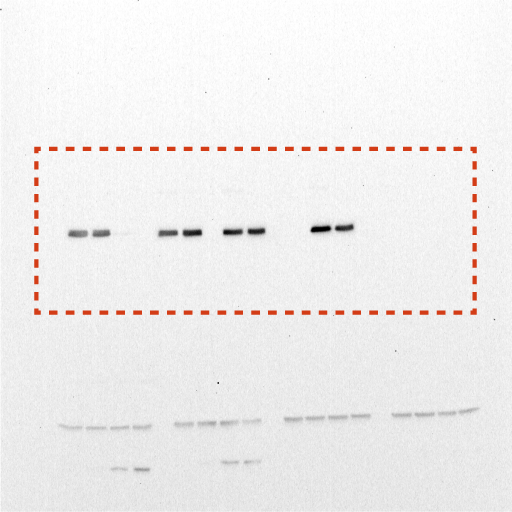

Supplement: Figure 1—figure supplement 2—source data 1. [file elife-79855-fig1-figsupp2-data1.zip › Figure 1 - figure supplement 2 - source data 1/Original files/Figure 1 - figure supplement 2 - source data 1 - highlighted/Tubulin - highlighted.tif]

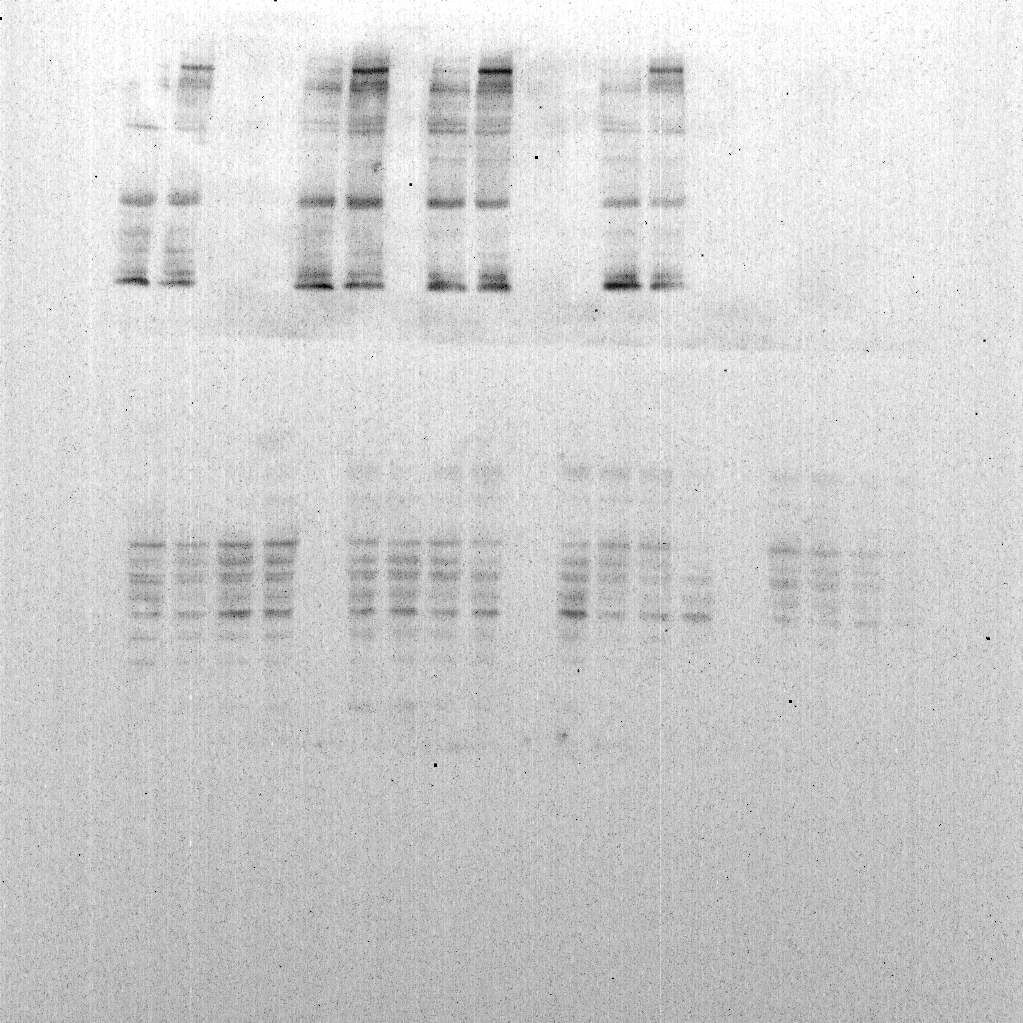

Supplement: Figure 1—figure supplement 2—source data 1. [file elife-79855-fig1-figsupp2-data1.zip › Figure 1 - figure supplement 2 - source data 1/Original files/Figure 1 - figure supplement 2 - source data 1 - unmodified/Afadin pY1230 - top blot.tif]

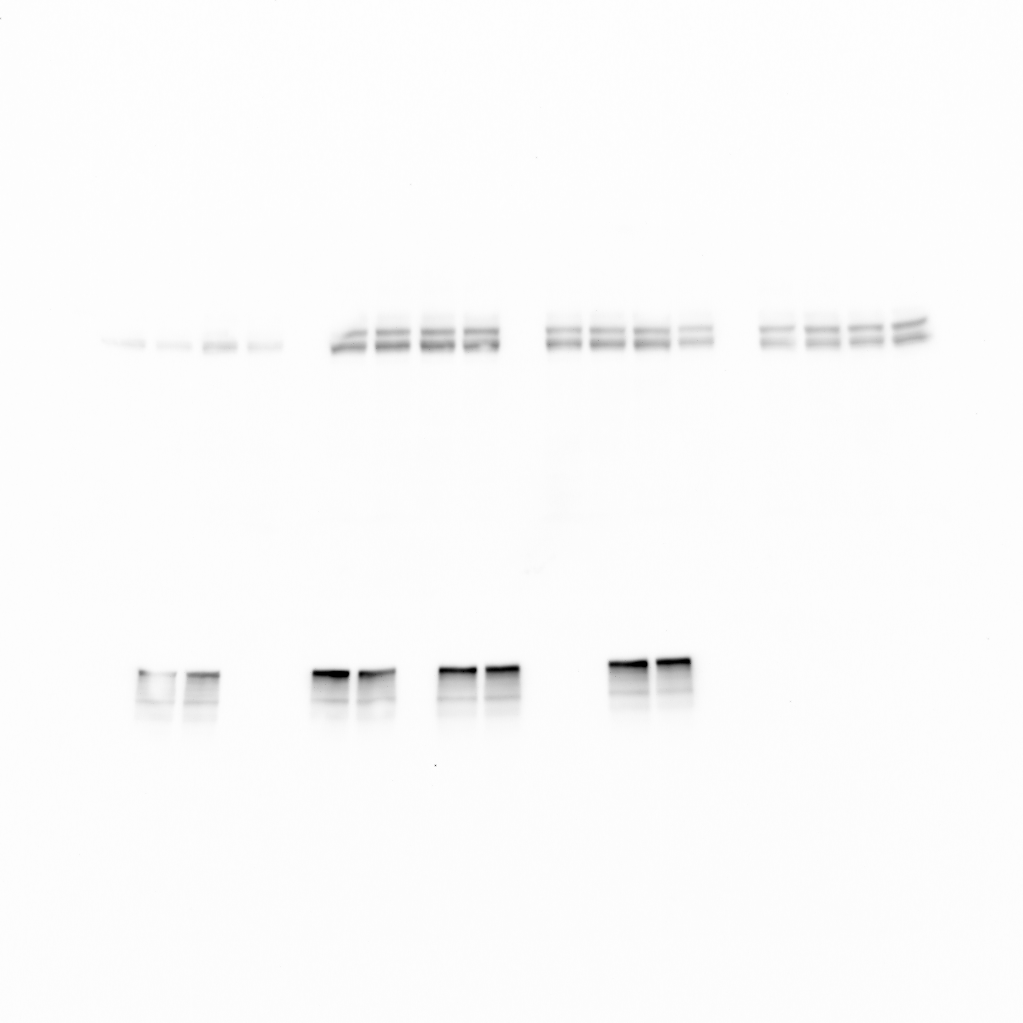

Supplement: Figure 1—figure supplement 2—source data 1. [file elife-79855-fig1-figsupp2-data1.zip › Figure 1 - figure supplement 2 - source data 1/Original files/Figure 1 - figure supplement 2 - source data 1 - unmodified/Afadin total - bottom blot.tif]

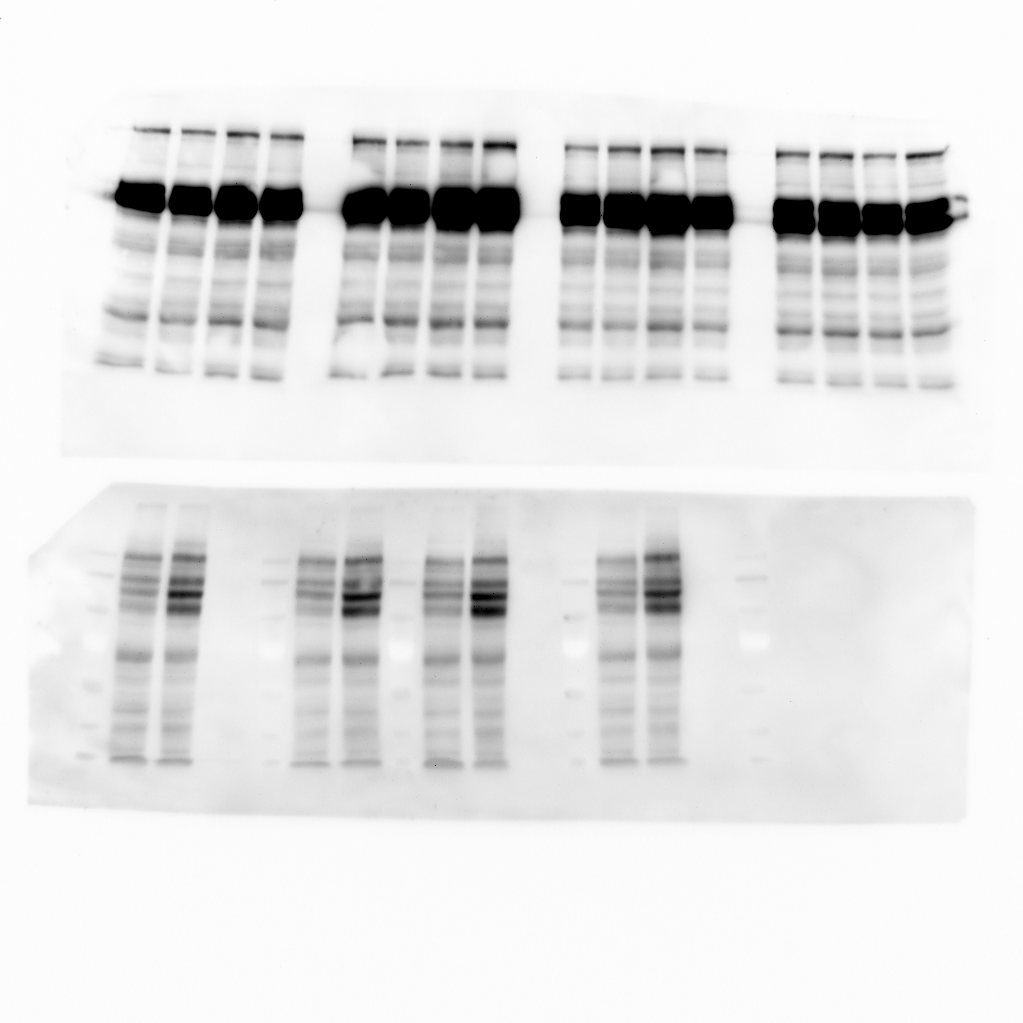

Supplement: Figure 1—figure supplement 2—source data 1. [file elife-79855-fig1-figsupp2-data1.zip › Figure 1 - figure supplement 2 - source data 1/Original files/Figure 1 - figure supplement 2 - source data 1 - unmodified/p120 catenin pY228 - bottom blot.tif]

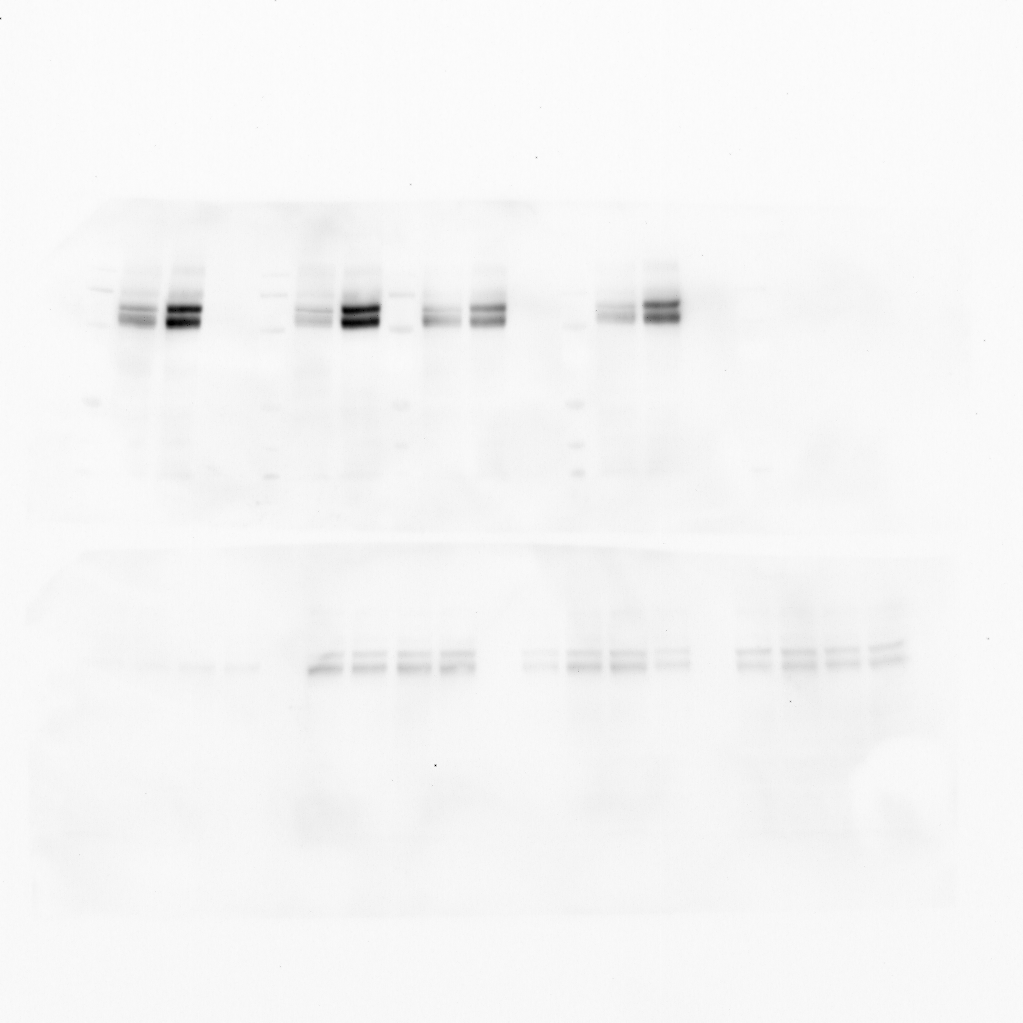

Supplement: Figure 1—figure supplement 2—source data 1. [file elife-79855-fig1-figsupp2-data1.zip › Figure 1 - figure supplement 2 - source data 1/Original files/Figure 1 - figure supplement 2 - source data 1 - unmodified/p120 catenin pY904 - top blot.tif]

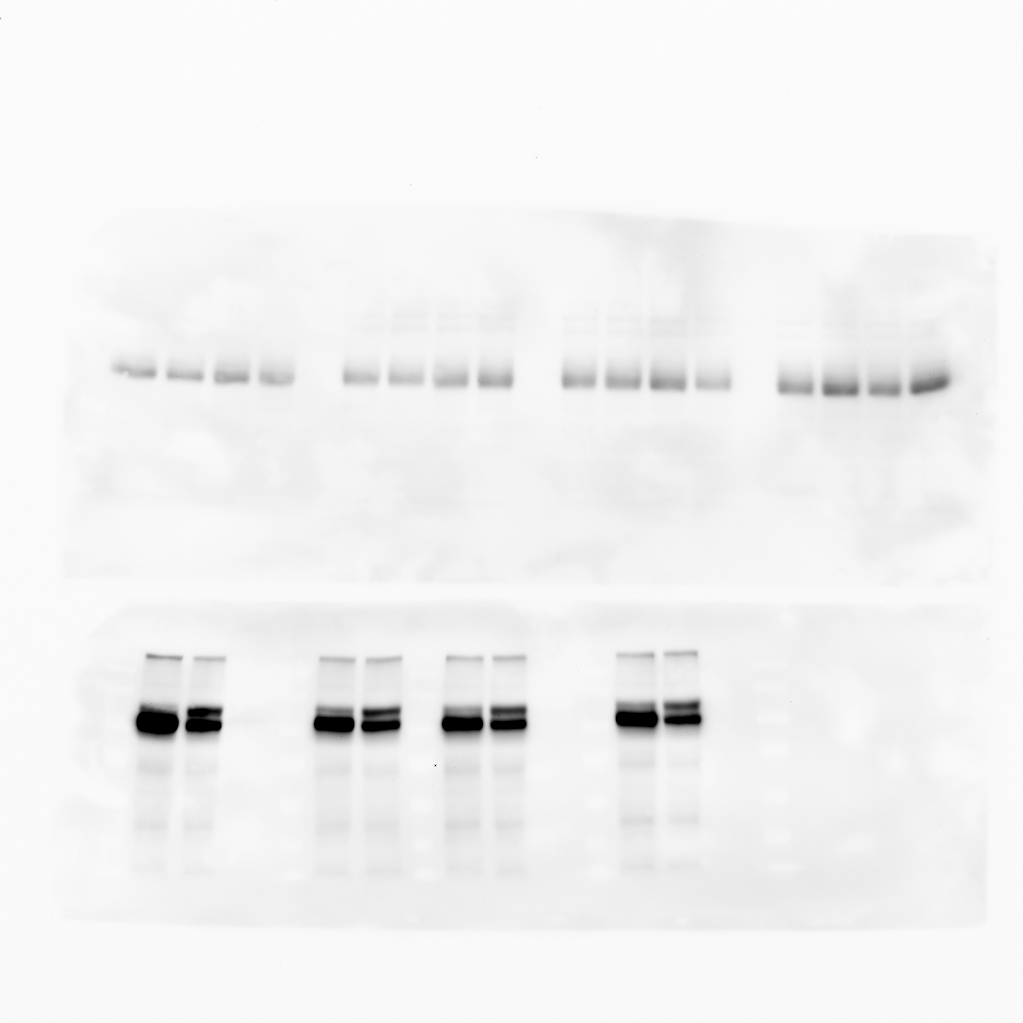

Supplement: Figure 1—figure supplement 2—source data 1. [file elife-79855-fig1-figsupp2-data1.zip › Figure 1 - figure supplement 2 - source data 1/Original files/Figure 1 - figure supplement 2 - source data 1 - unmodified/p120 catenin total - bottom blot.tif]

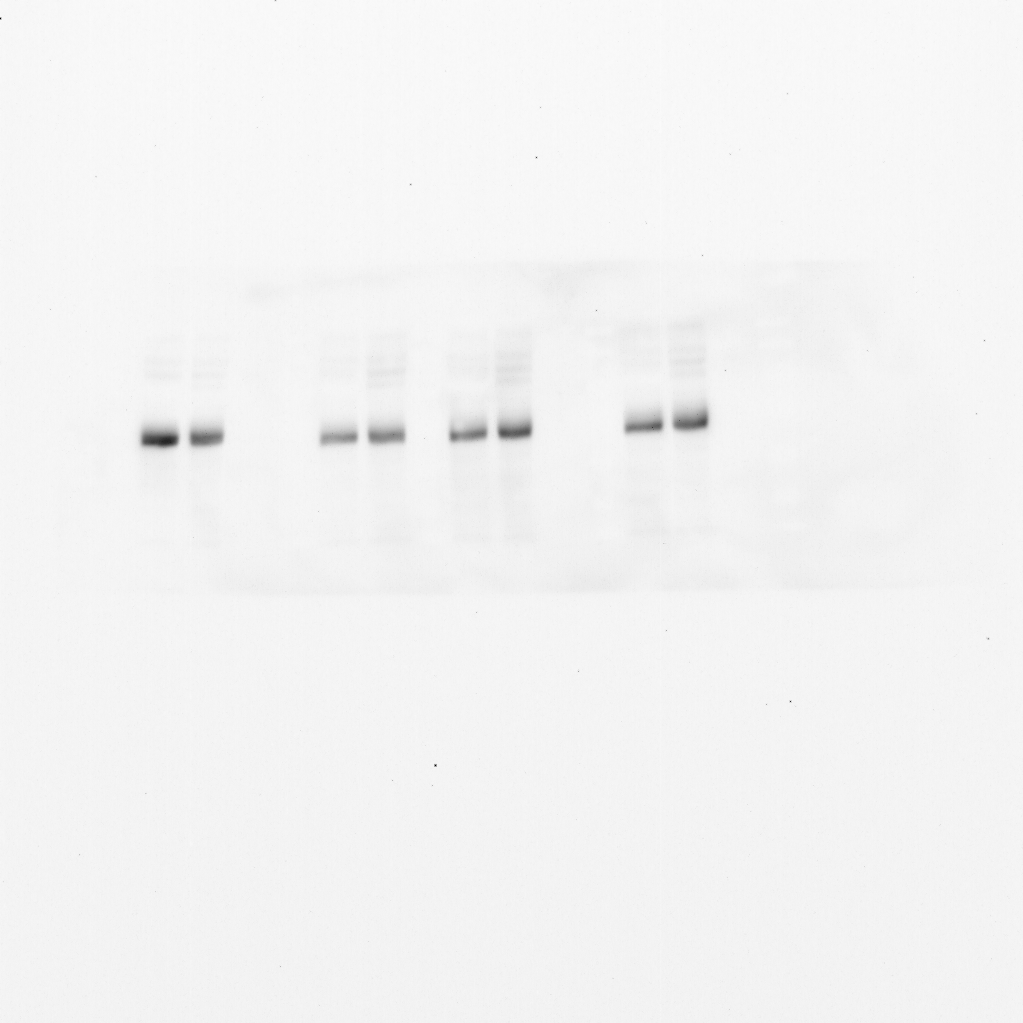

Supplement: Figure 1—figure supplement 2—source data 1. [file elife-79855-fig1-figsupp2-data1.zip › Figure 1 - figure supplement 2 - source data 1/Original files/Figure 1 - figure supplement 2 - source data 1 - unmodified/Paxillin pY118.tif]

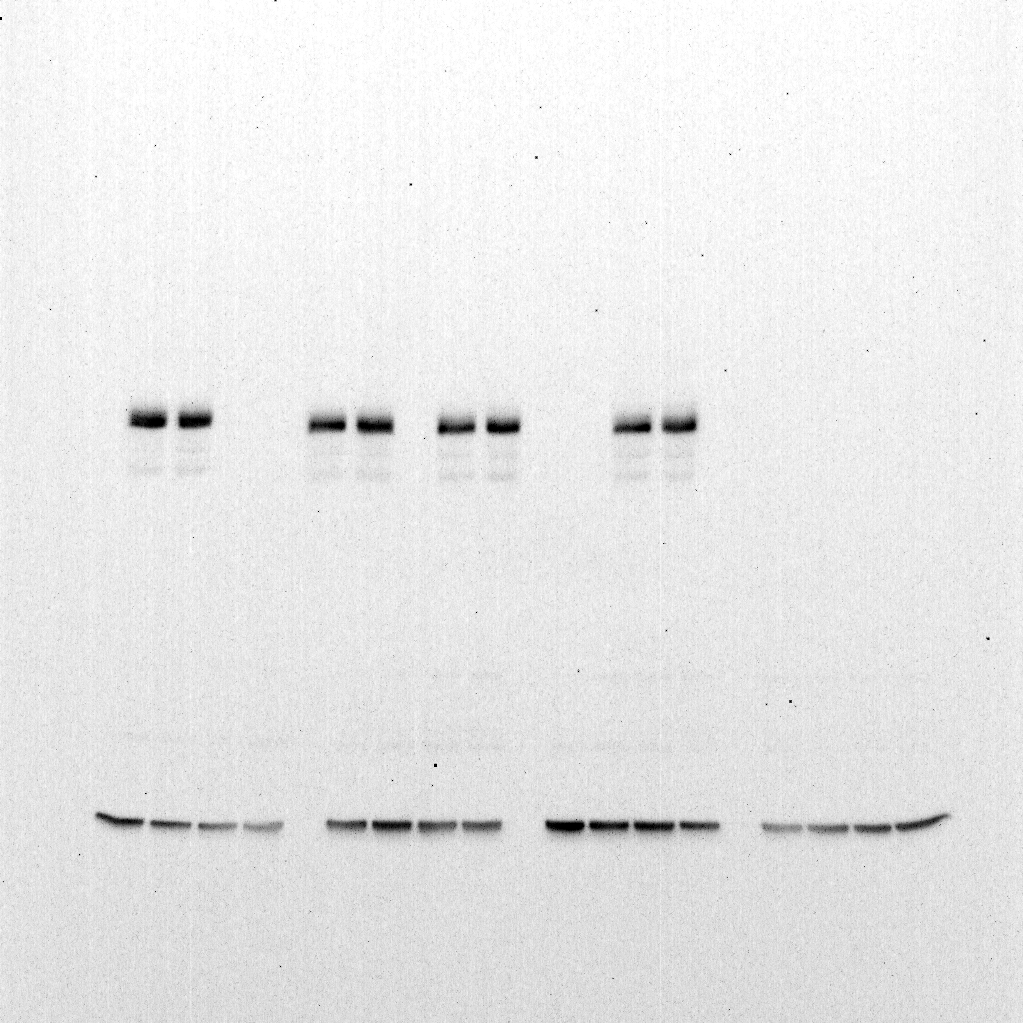

Supplement: Figure 1—figure supplement 2—source data 1. [file elife-79855-fig1-figsupp2-data1.zip › Figure 1 - figure supplement 2 - source data 1/Original files/Figure 1 - figure supplement 2 - source data 1 - unmodified/Paxillin total - top blot.tif]

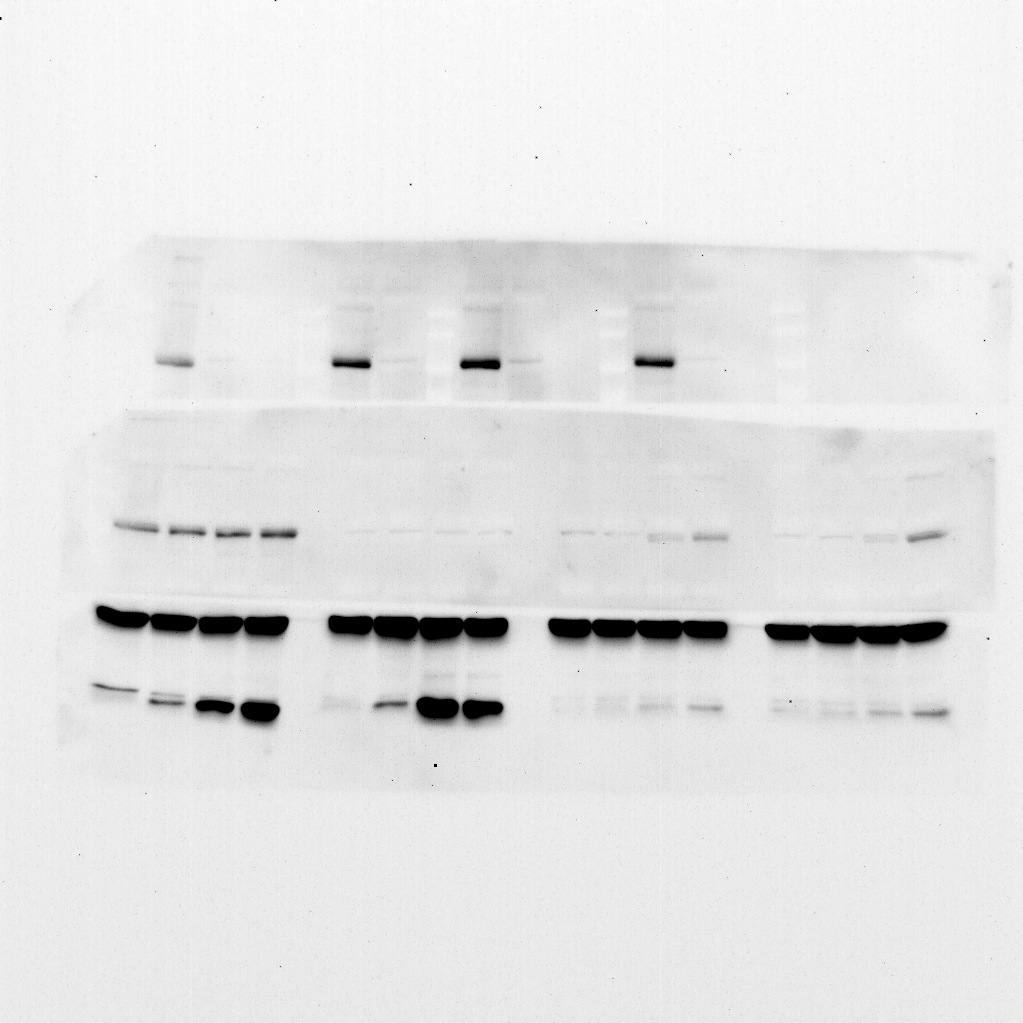

Supplement: Figure 1—figure supplement 2—source data 1. [file elife-79855-fig1-figsupp2-data1.zip › Figure 1 - figure supplement 2 - source data 1/Original files/Figure 1 - figure supplement 2 - source data 1 - unmodified/PTPRK - top blot.tif]

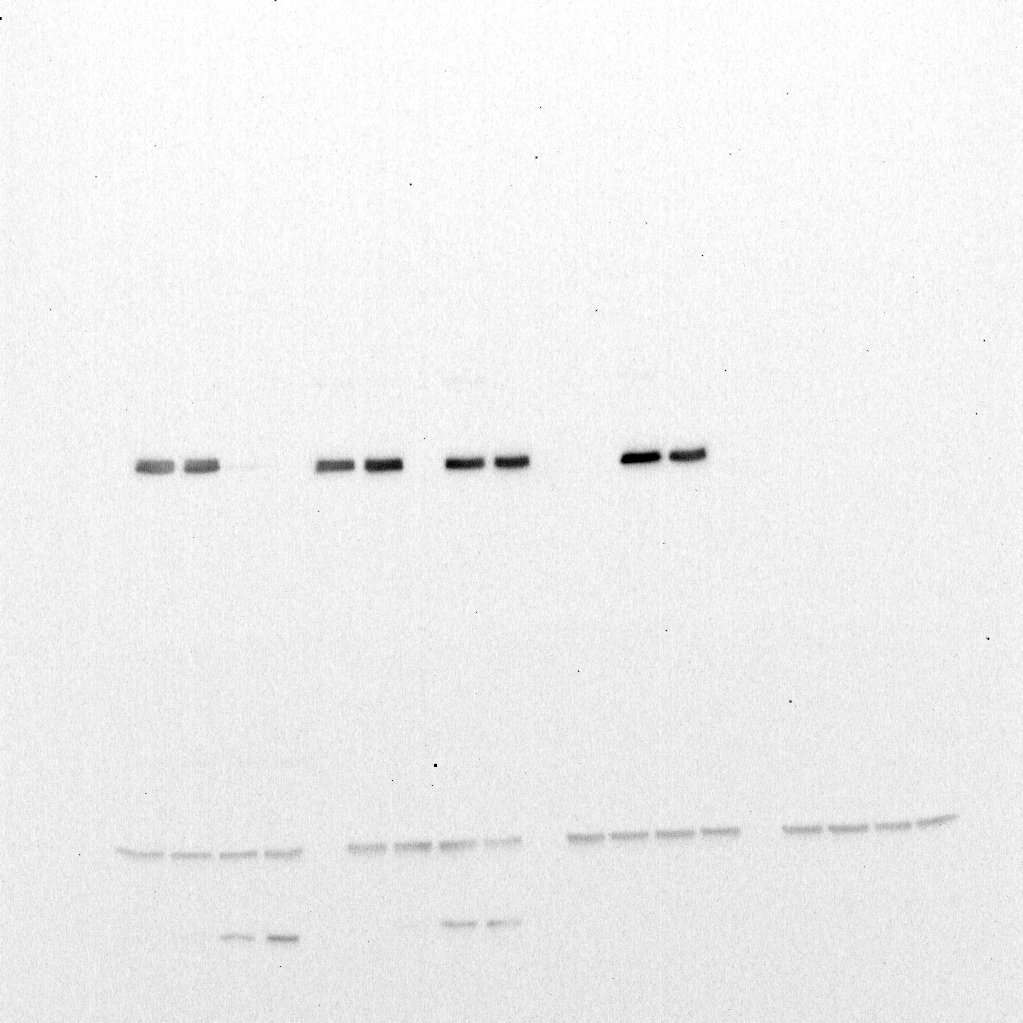

Supplement: Figure 1—figure supplement 2—source data 1. [file elife-79855-fig1-figsupp2-data1.zip › Figure 1 - figure supplement 2 - source data 1/Original files/Figure 1 - figure supplement 2 - source data 1 - unmodified/Tubulin - top blot.tif]

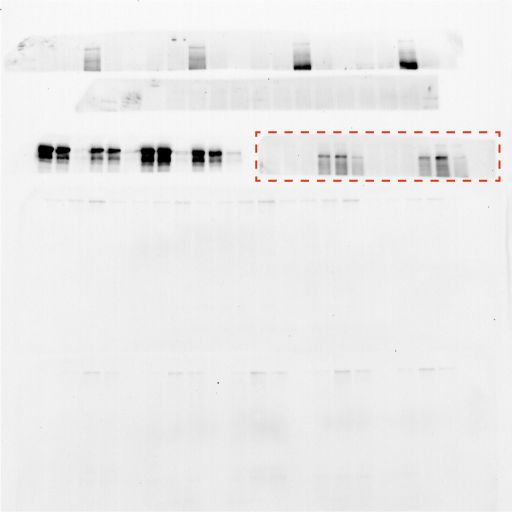

Supplement: Figure 1—figure supplement 2—source data 2. [file elife-79855-fig1-figsupp2-data2.zip › Figure 1 - figure supplement 2 - source data 2/Original files/Figure 1 - figure supplement 2 - source data 2 - highlighted/Afadin pY1230 - highlighted with red box.tif]

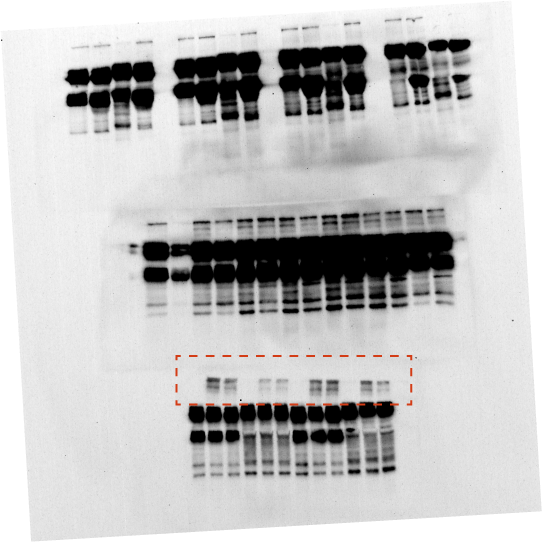

Supplement: Figure 1—figure supplement 2—source data 2. [file elife-79855-fig1-figsupp2-data2.zip › Figure 1 - figure supplement 2 - source data 2/Original files/Figure 1 - figure supplement 2 - source data 2 - highlighted/Afadin total - highlighted with red box.tif]

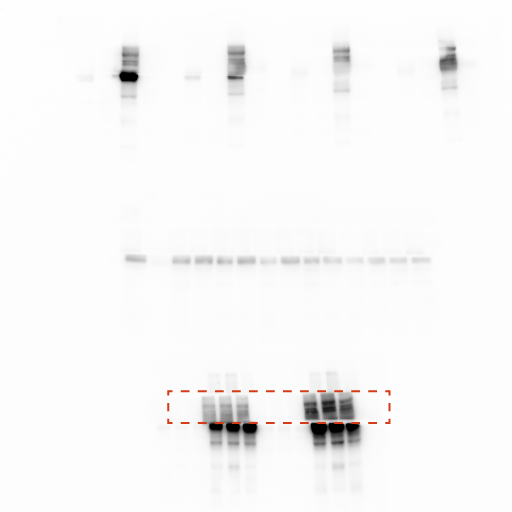

Supplement: Figure 1—figure supplement 2—source data 2. [file elife-79855-fig1-figsupp2-data2.zip › Figure 1 - figure supplement 2 - source data 2/Original files/Figure 1 - figure supplement 2 - source data 2 - highlighted/p120 catenin pY228 - highlighted with red box.tif]

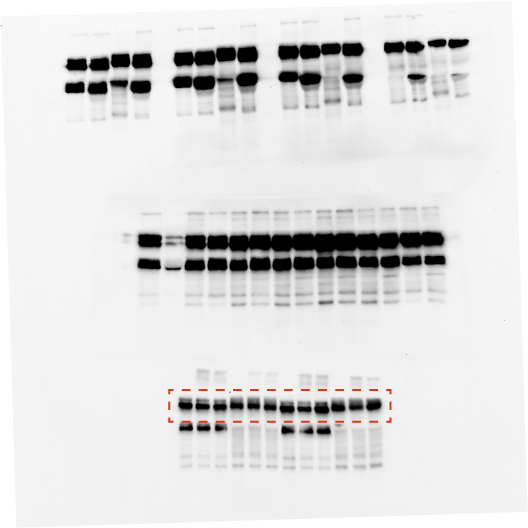

Supplement: Figure 1—figure supplement 2—source data 2. [file elife-79855-fig1-figsupp2-data2.zip › Figure 1 - figure supplement 2 - source data 2/Original files/Figure 1 - figure supplement 2 - source data 2 - highlighted/p120 catenin total - highlighted with red box.tif]

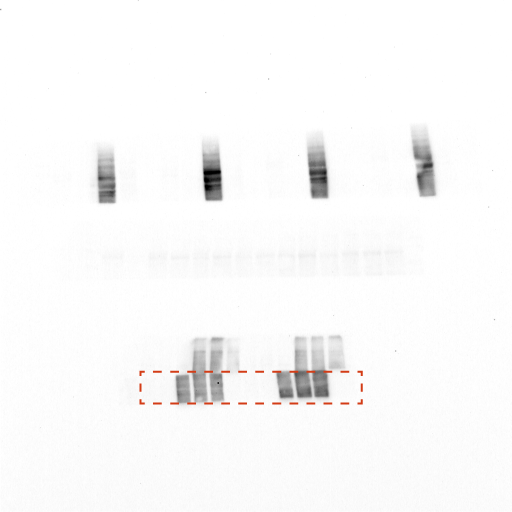

Supplement: Figure 1—figure supplement 2—source data 2. [file elife-79855-fig1-figsupp2-data2.zip › Figure 1 - figure supplement 2 - source data 2/Original files/Figure 1 - figure supplement 2 - source data 2 - highlighted/pTyr - highlighted with red box.tif]

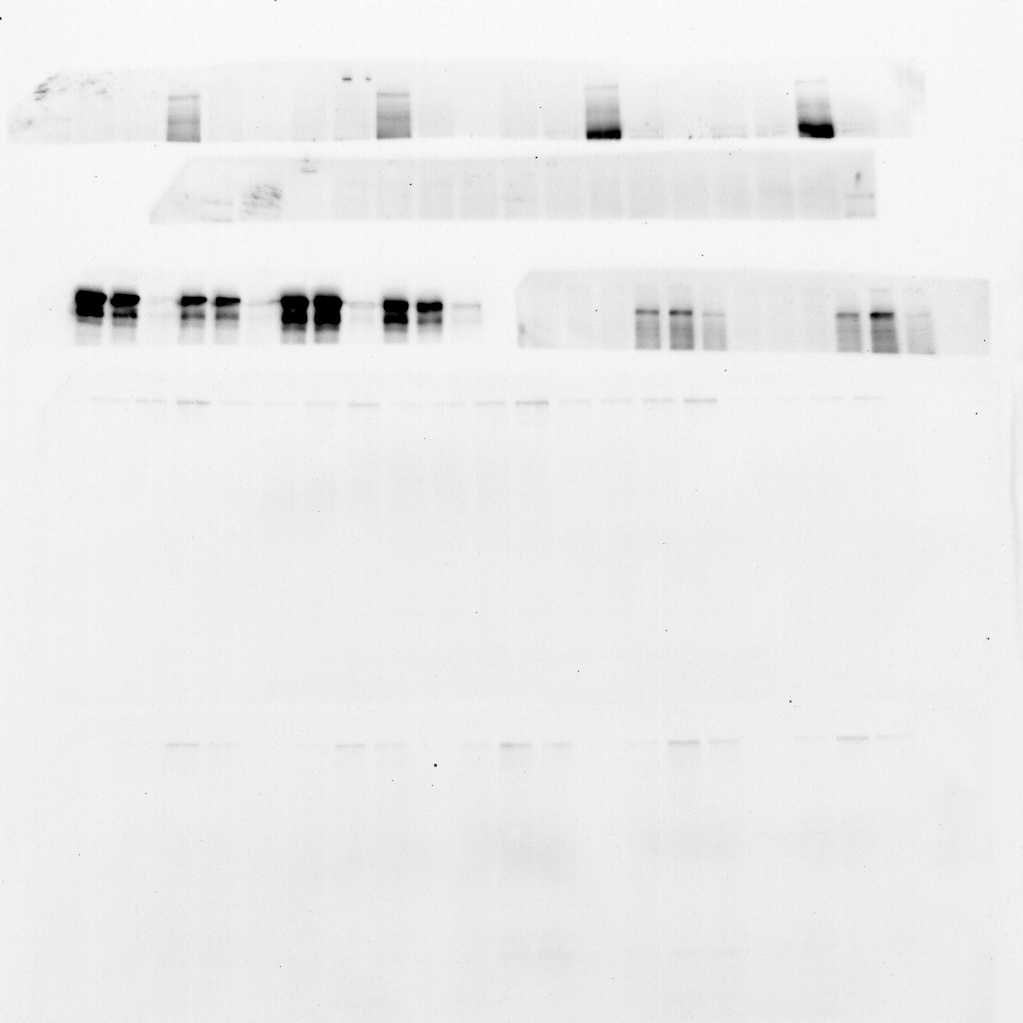

Supplement: Figure 1—figure supplement 2—source data 2. [file elife-79855-fig1-figsupp2-data2.zip › Figure 1 - figure supplement 2 - source data 2/Original files/Figure 1 - figure supplement 2 - source data 2 - unmodified/Afadin pY1230 - unmodified.tif]

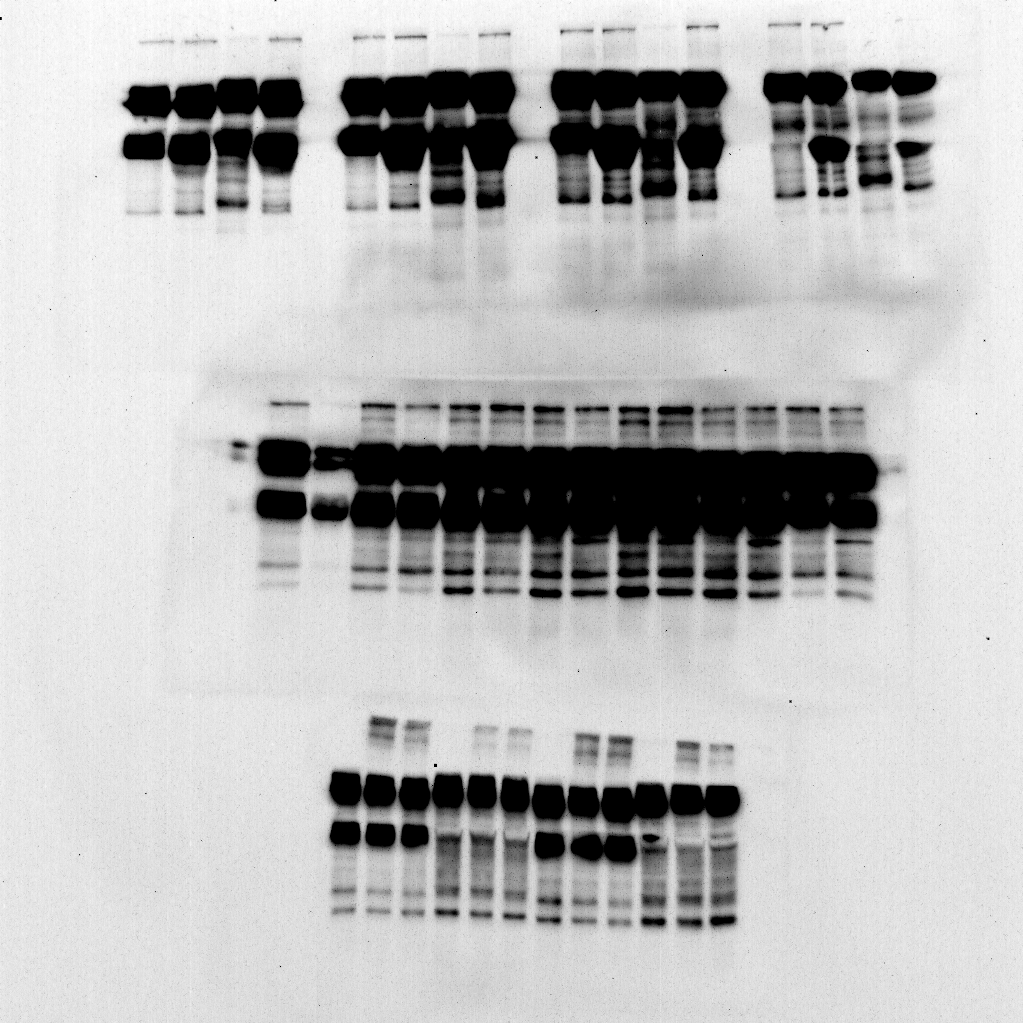

Supplement: Figure 1—figure supplement 2—source data 2. [file elife-79855-fig1-figsupp2-data2.zip › Figure 1 - figure supplement 2 - source data 2/Original files/Figure 1 - figure supplement 2 - source data 2 - unmodified/Afadin total unmodified.tif]

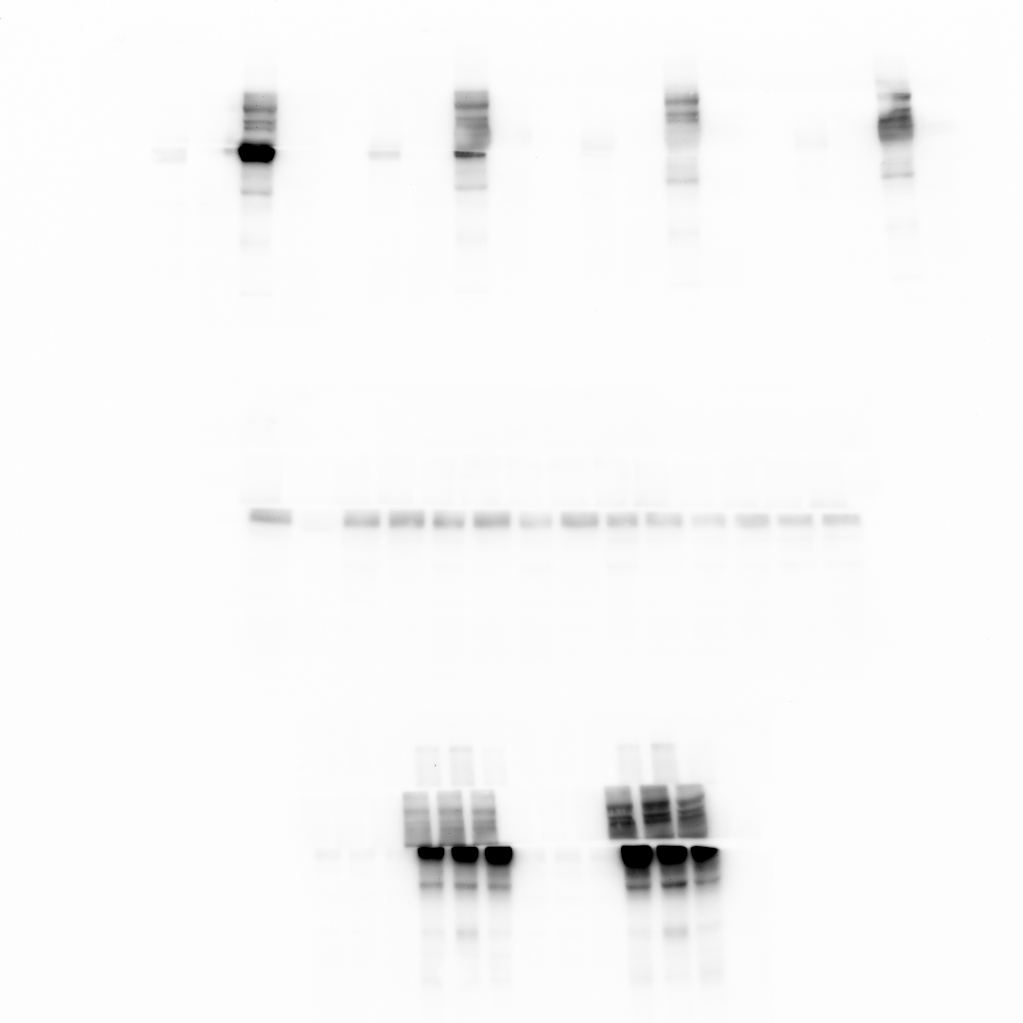

Supplement: Figure 1—figure supplement 2—source data 2. [file elife-79855-fig1-figsupp2-data2.zip › Figure 1 - figure supplement 2 - source data 2/Original files/Figure 1 - figure supplement 2 - source data 2 - unmodified/p120 catenin pY228 - unmodified.tif]

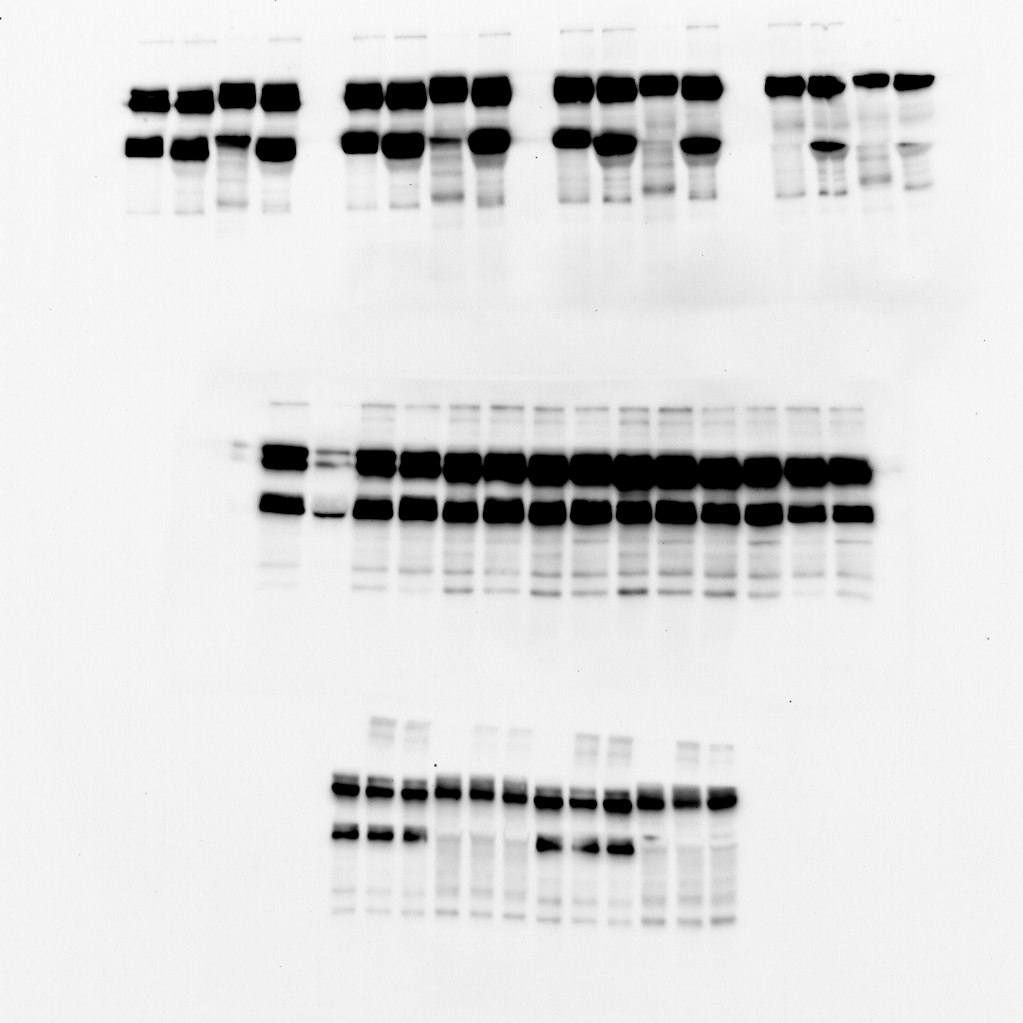

Supplement: Figure 1—figure supplement 2—source data 2. [file elife-79855-fig1-figsupp2-data2.zip › Figure 1 - figure supplement 2 - source data 2/Original files/Figure 1 - figure supplement 2 - source data 2 - unmodified/p120 catenin total - unmodified.tif]

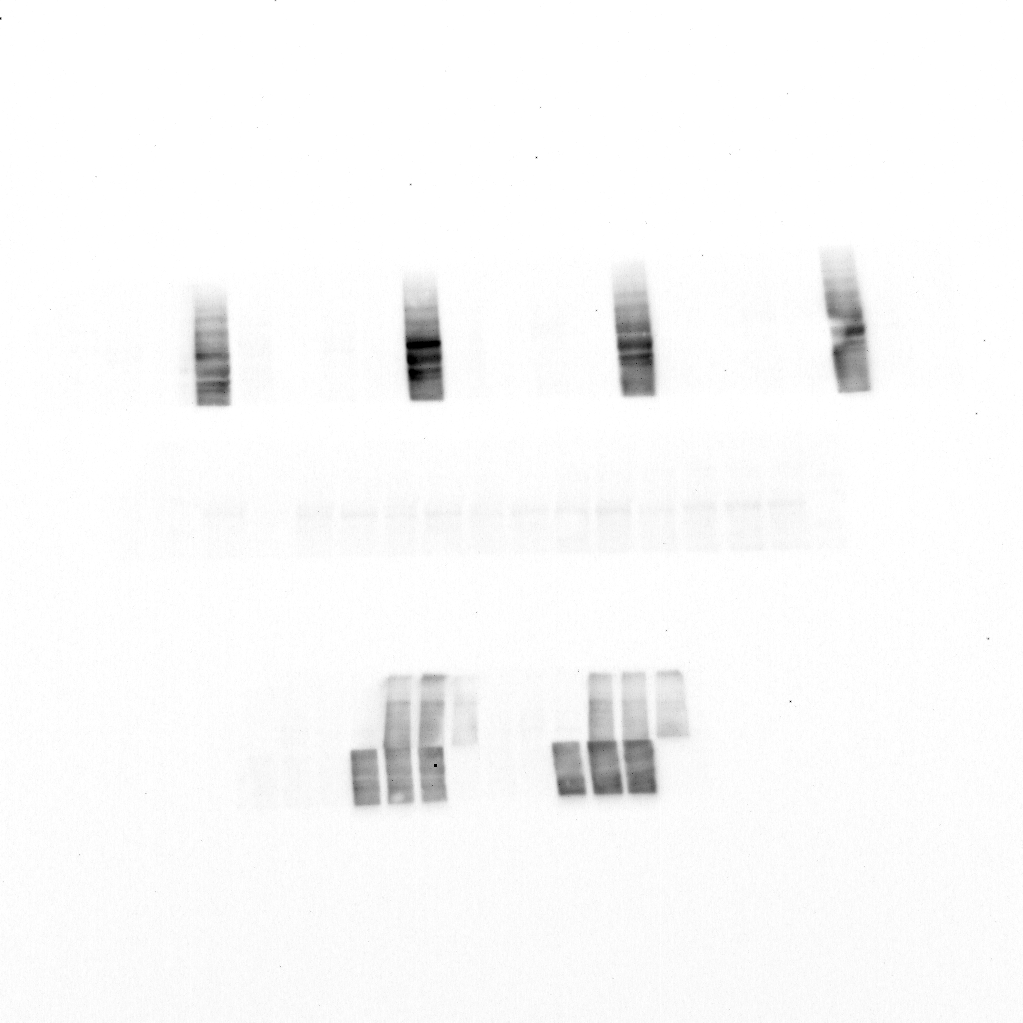

Supplement: Figure 1—figure supplement 2—source data 2. [file elife-79855-fig1-figsupp2-data2.zip › Figure 1 - figure supplement 2 - source data 2/Original files/Figure 1 - figure supplement 2 - source data 2 - unmodified/pTyr - unmodified.tif]

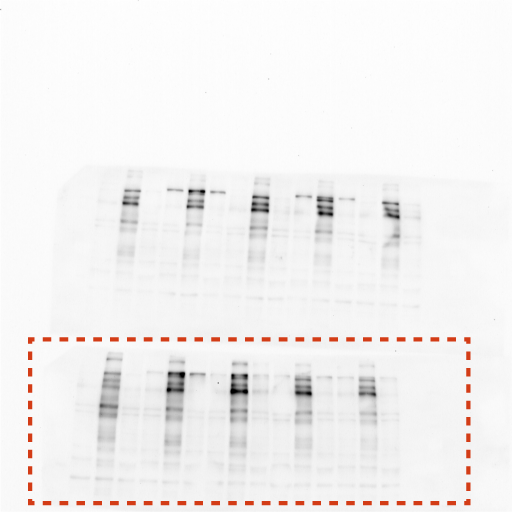

Supplement: Figure 1—figure supplement 2—source data 3. [file elife-79855-fig1-figsupp2-data3.zip › Figure 1 - figure supplement 2 - source data 3/Original files/Figure 1 - figure supplement 2 - source data 3 - highlighted/Afadin pY1230 - highlighted.tif]

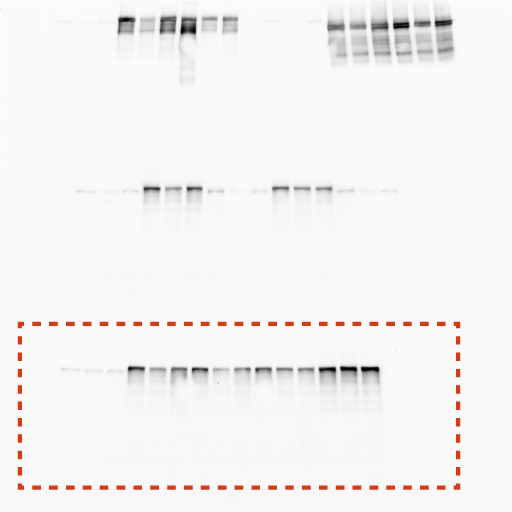

Supplement: Figure 1—figure supplement 2—source data 3. [file elife-79855-fig1-figsupp2-data3.zip › Figure 1 - figure supplement 2 - source data 3/Original files/Figure 1 - figure supplement 2 - source data 3 - highlighted/Afadin total - highlighted.tif]

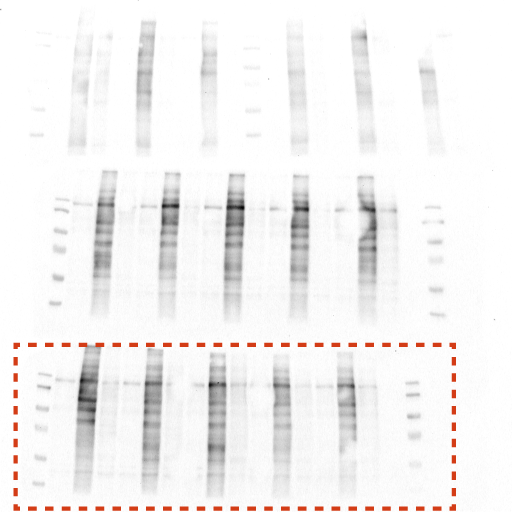

Supplement: Figure 1—figure supplement 2—source data 3. [file elife-79855-fig1-figsupp2-data3.zip › Figure 1 - figure supplement 2 - source data 3/Original files/Figure 1 - figure supplement 2 - source data 3 - highlighted/pTyr - highlighted.tif]

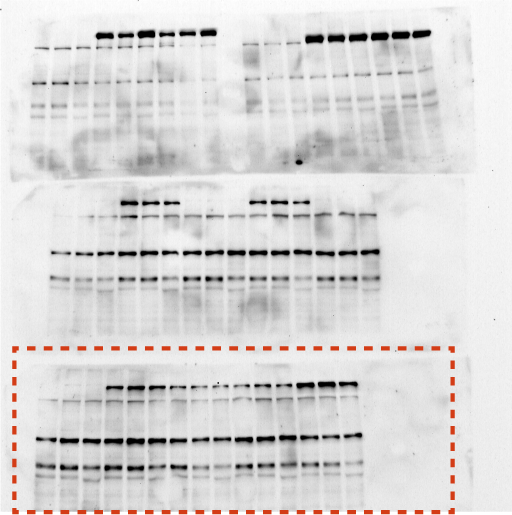

Supplement: Figure 1—figure supplement 2—source data 3. [file elife-79855-fig1-figsupp2-data3.zip › Figure 1 - figure supplement 2 - source data 3/Original files/Figure 1 - figure supplement 2 - source data 3 - highlighted/RFP - highlighted.tif]

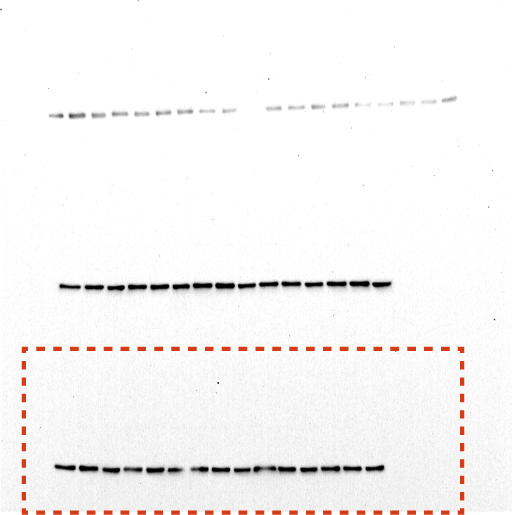

Supplement: Figure 1—figure supplement 2—source data 3. [file elife-79855-fig1-figsupp2-data3.zip › Figure 1 - figure supplement 2 - source data 3/Original files/Figure 1 - figure supplement 2 - source data 3 - highlighted/Tubulin - highlighted.tif]

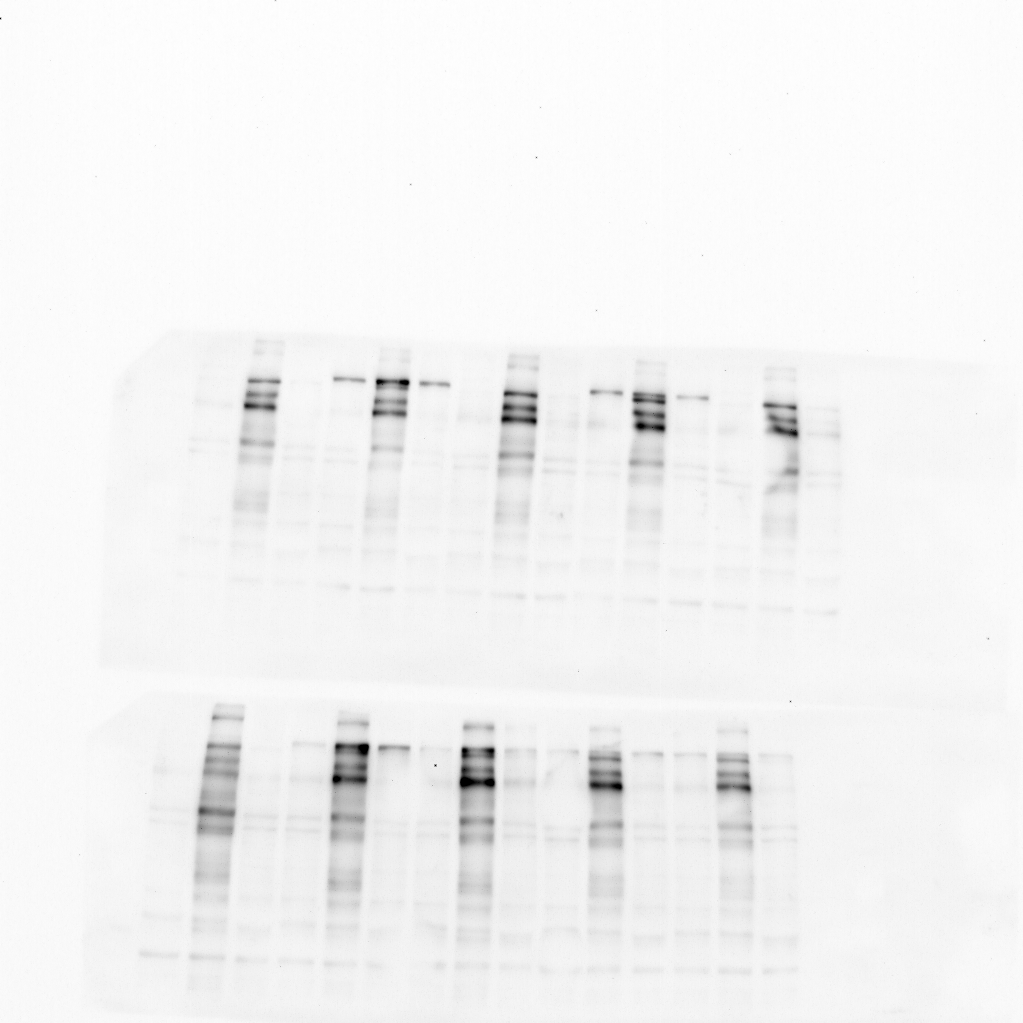

Supplement: Figure 1—figure supplement 2—source data 3. [file elife-79855-fig1-figsupp2-data3.zip › Figure 1 - figure supplement 2 - source data 3/Original files/Figure 1 - figure supplement 2 - source data 3 - unmodified/Afadin pY1230 - bottom blot.tif]

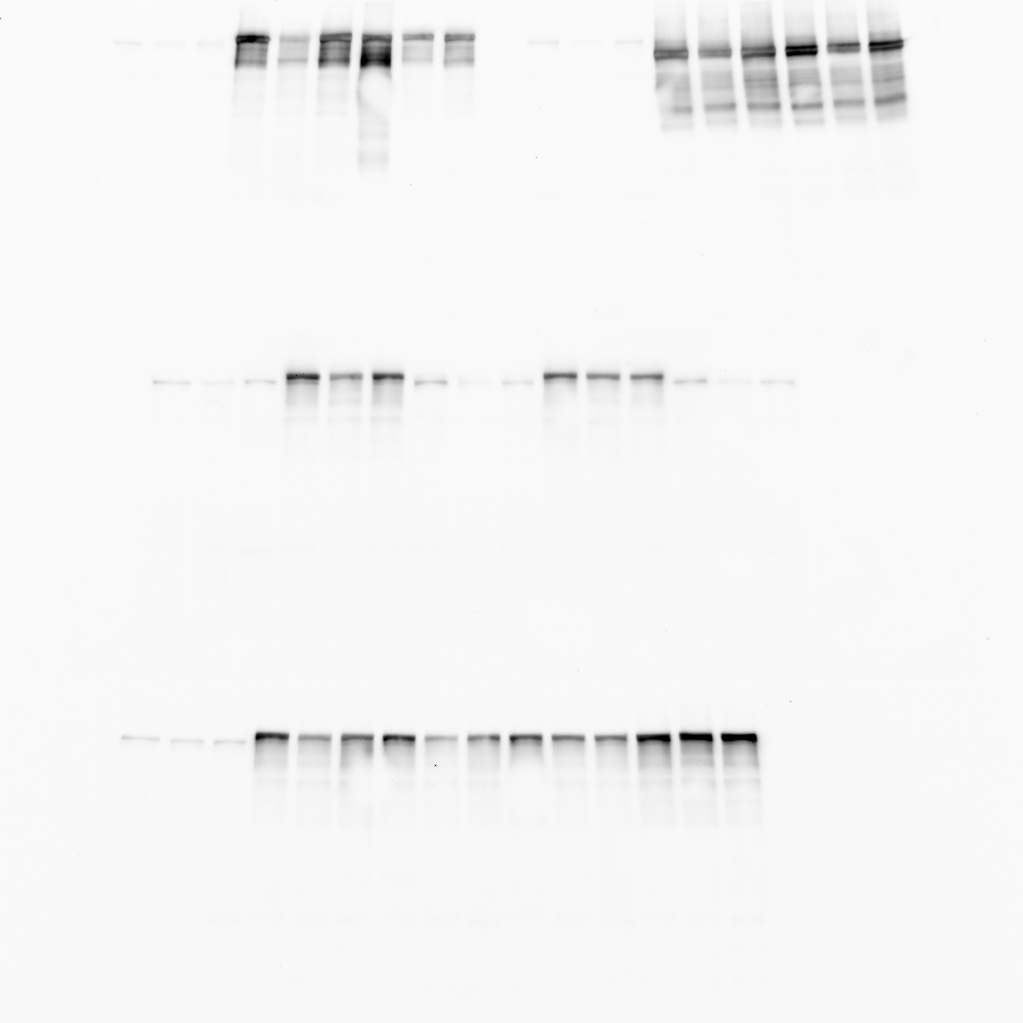

Supplement: Figure 1—figure supplement 2—source data 3. [file elife-79855-fig1-figsupp2-data3.zip › Figure 1 - figure supplement 2 - source data 3/Original files/Figure 1 - figure supplement 2 - source data 3 - unmodified/Afadin total - bottom blot.tif]

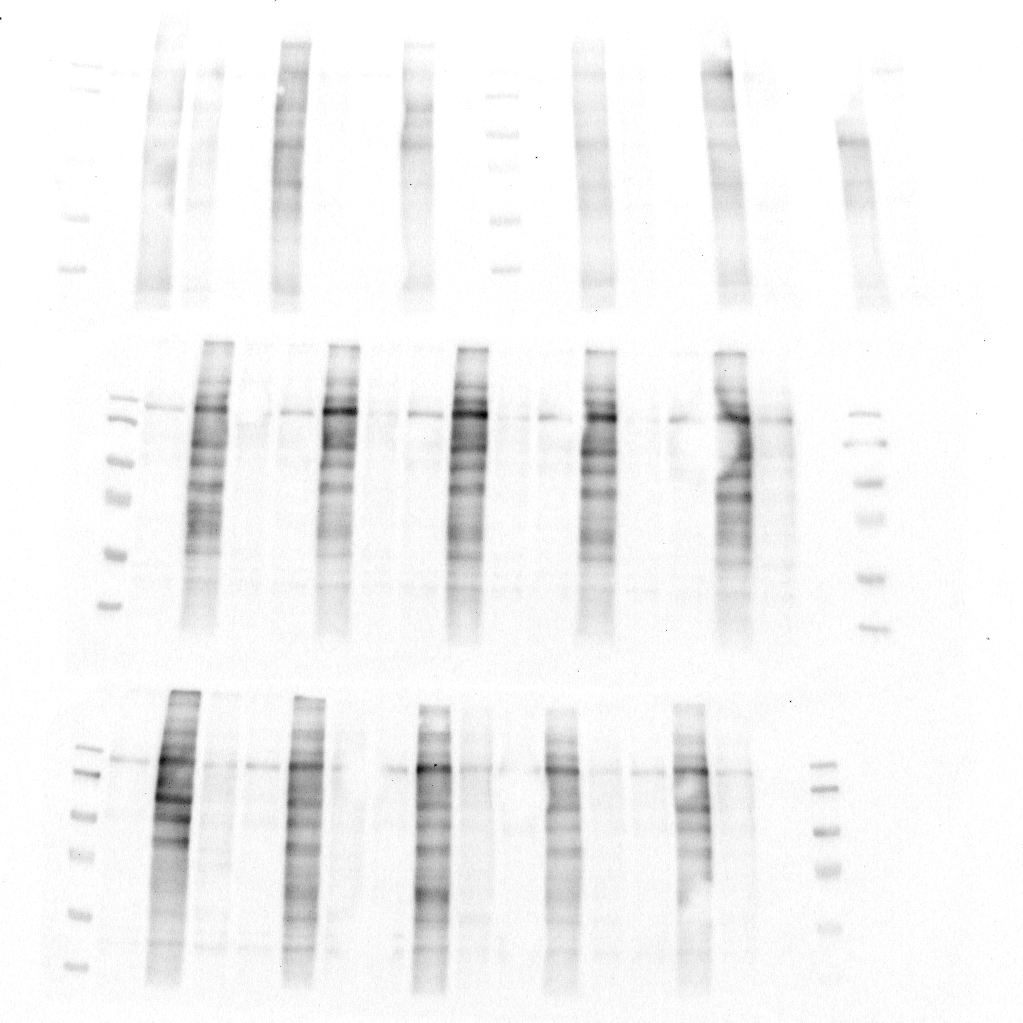

Supplement: Figure 1—figure supplement 2—source data 3. [file elife-79855-fig1-figsupp2-data3.zip › Figure 1 - figure supplement 2 - source data 3/Original files/Figure 1 - figure supplement 2 - source data 3 - unmodified/pTyr - bottom blot.tif]

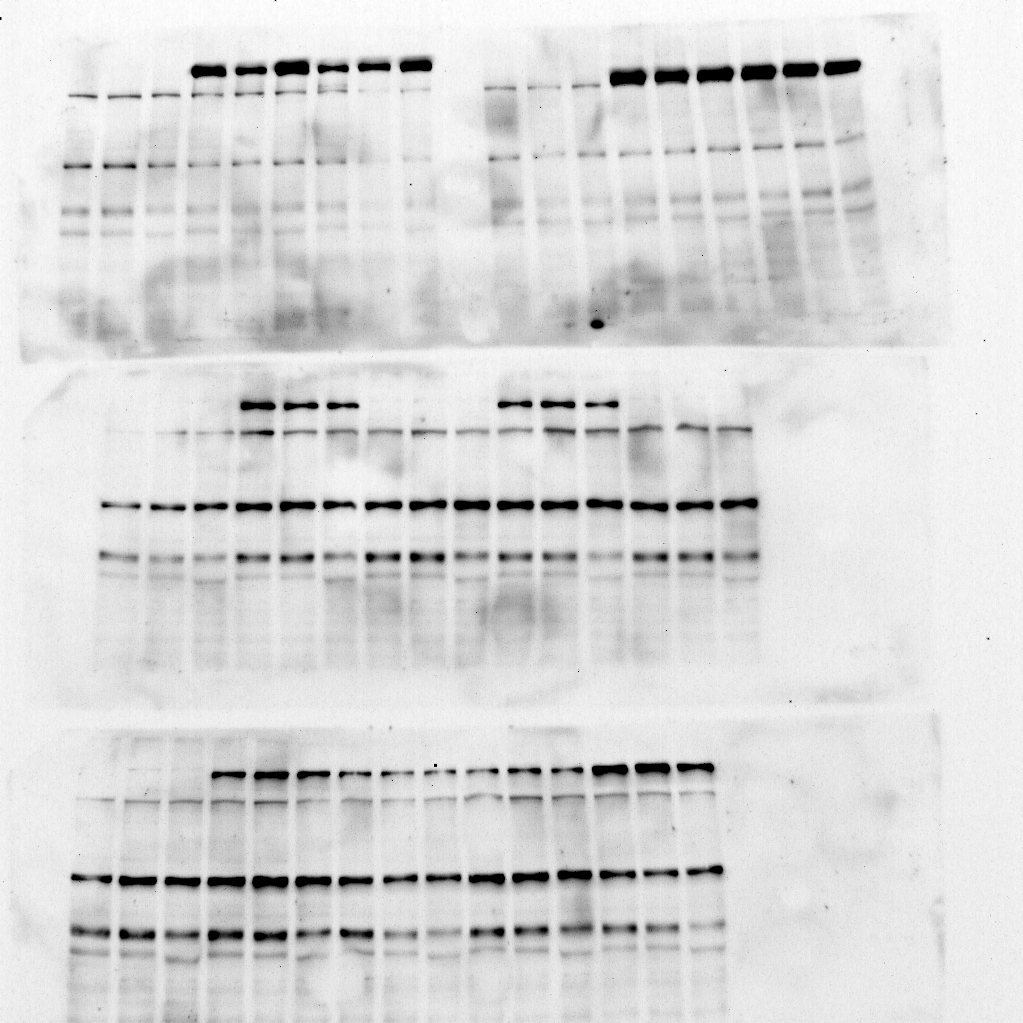

Supplement: Figure 1—figure supplement 2—source data 3. [file elife-79855-fig1-figsupp2-data3.zip › Figure 1 - figure supplement 2 - source data 3/Original files/Figure 1 - figure supplement 2 - source data 3 - unmodified/RFP- bottom blot.tif]

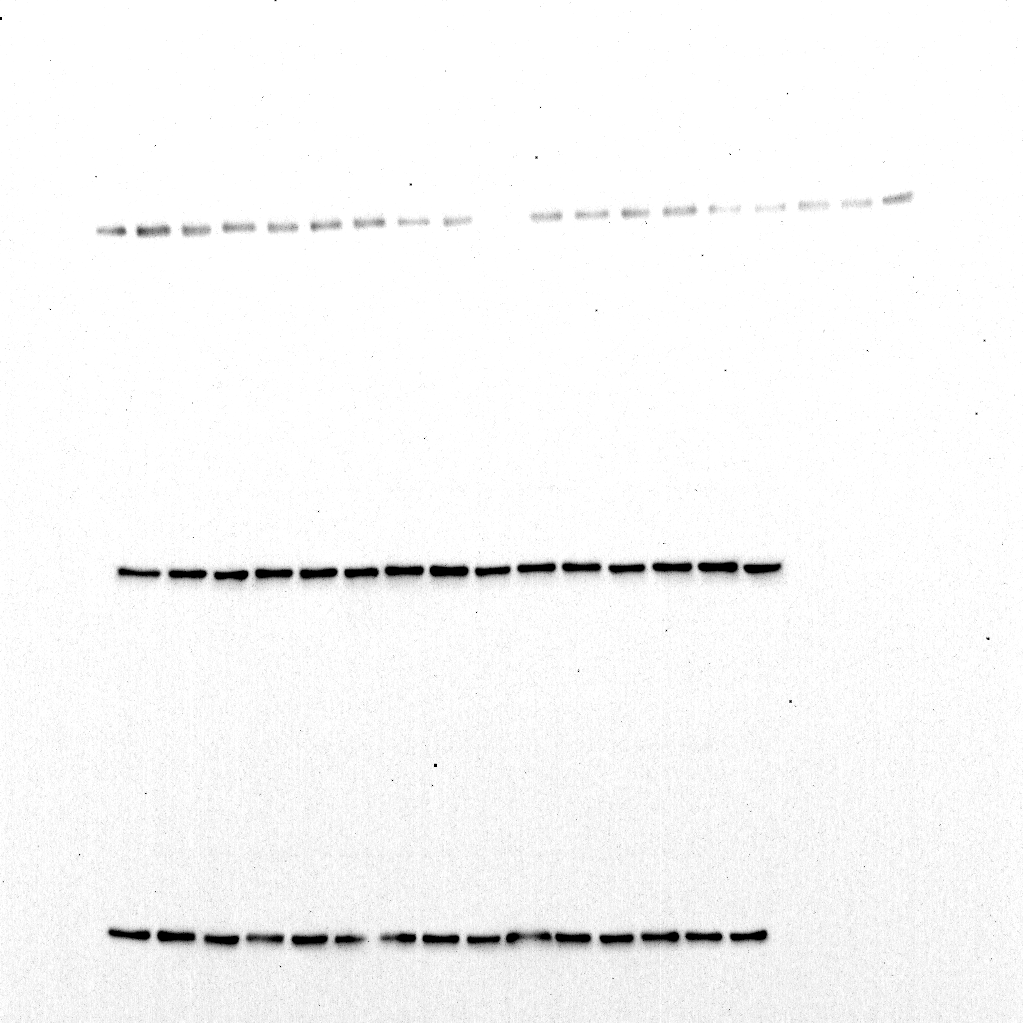

Supplement: Figure 1—figure supplement 2—source data 3. [file elife-79855-fig1-figsupp2-data3.zip › Figure 1 - figure supplement 2 - source data 3/Original files/Figure 1 - figure supplement 2 - source data 3 - unmodified/Tubulin - bottom blot.tif]

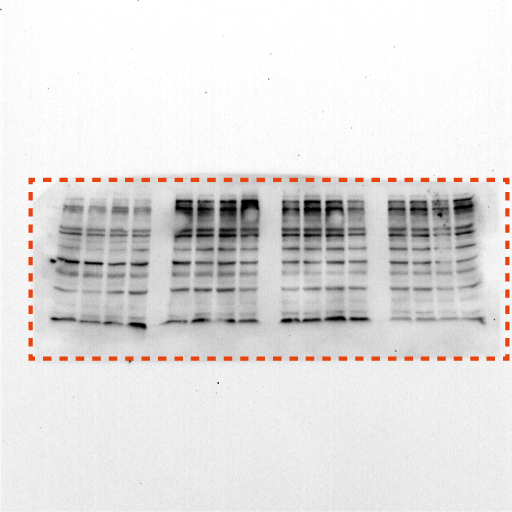

Supplement: Figure 1—figure supplement 2—source data 5. [file elife-79855-fig1-figsupp2-data5.zip › Figure 1 - figure supplement 2 - source data 5/Original files/Figure 1 - figure supplement 2 - source data 5 - highlighted/Afadin pY1230 high exp - highlighted.tif]

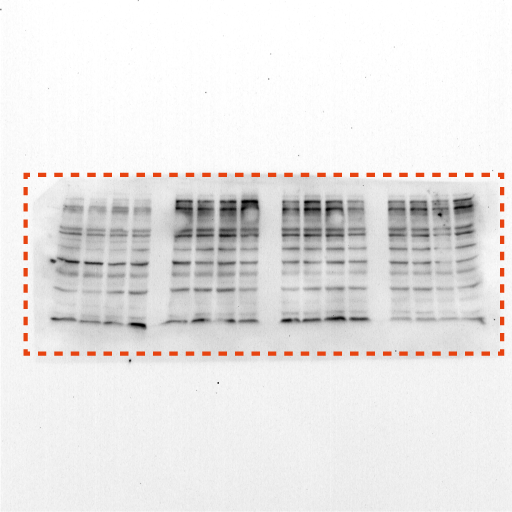

Supplement: Figure 1—figure supplement 2—source data 5. [file elife-79855-fig1-figsupp2-data5.zip › Figure 1 - figure supplement 2 - source data 5/Original files/Figure 1 - figure supplement 2 - source data 5 - highlighted/Afadin pY1230 low exp - highlighted.tif]

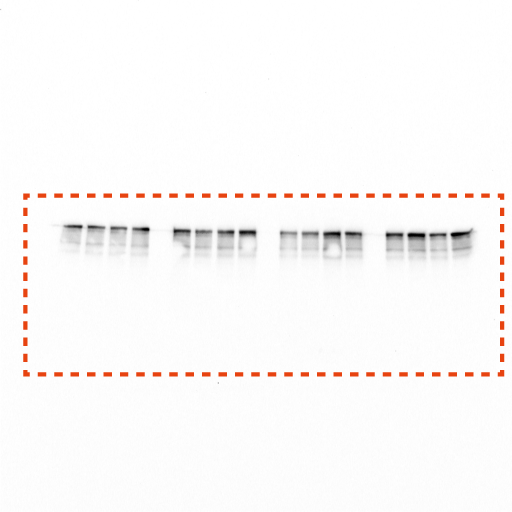

Supplement: Figure 1—figure supplement 2—source data 5. [file elife-79855-fig1-figsupp2-data5.zip › Figure 1 - figure supplement 2 - source data 5/Original files/Figure 1 - figure supplement 2 - source data 5 - highlighted/Afadin total - highlighted.tif]

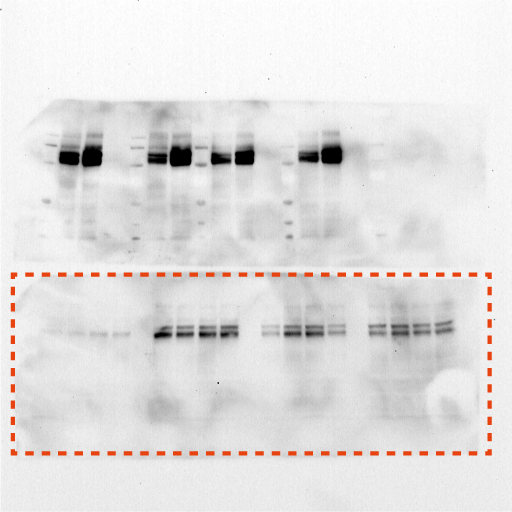

Supplement: Figure 1—figure supplement 2—source data 5. [file elife-79855-fig1-figsupp2-data5.zip › Figure 1 - figure supplement 2 - source data 5/Original files/Figure 1 - figure supplement 2 - source data 5 - highlighted/p120 catenin pY228 - highlighted.tif]

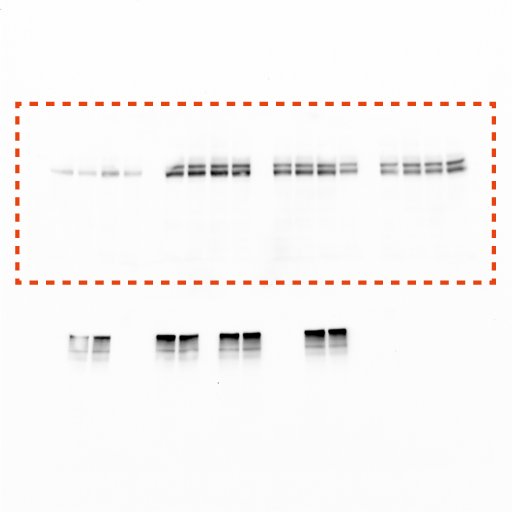

Supplement: Figure 1—figure supplement 2—source data 5. [file elife-79855-fig1-figsupp2-data5.zip › Figure 1 - figure supplement 2 - source data 5/Original files/Figure 1 - figure supplement 2 - source data 5 - highlighted/p120 catenin pY904 - highlighted.tif]

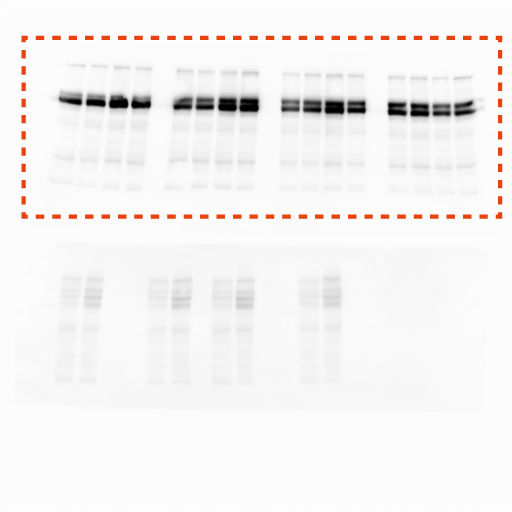

Supplement: Figure 1—figure supplement 2—source data 5. [file elife-79855-fig1-figsupp2-data5.zip › Figure 1 - figure supplement 2 - source data 5/Original files/Figure 1 - figure supplement 2 - source data 5 - highlighted/p120 catenin total - highlighted.tif]

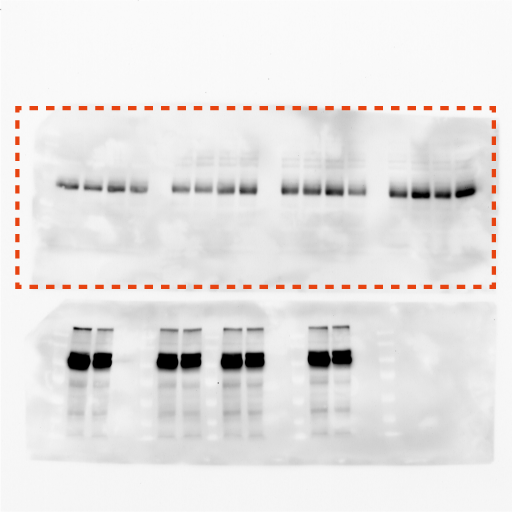

Supplement: Figure 1—figure supplement 2—source data 5. [file elife-79855-fig1-figsupp2-data5.zip › Figure 1 - figure supplement 2 - source data 5/Original files/Figure 1 - figure supplement 2 - source data 5 - highlighted/Paxillin pY118 - highlighted.tif]

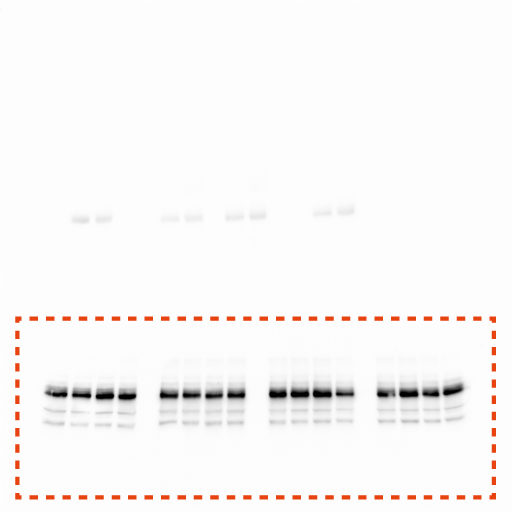

Supplement: Figure 1—figure supplement 2—source data 5. [file elife-79855-fig1-figsupp2-data5.zip › Figure 1 - figure supplement 2 - source data 5/Original files/Figure 1 - figure supplement 2 - source data 5 - highlighted/Paxillin total - highlighted.tif]

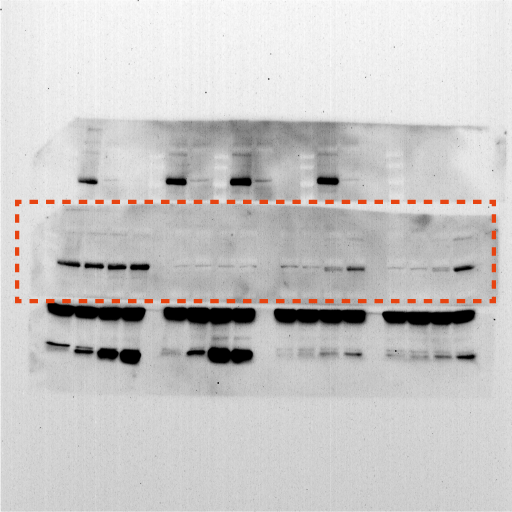

Supplement: Figure 1—figure supplement 2—source data 5. [file elife-79855-fig1-figsupp2-data5.zip › Figure 1 - figure supplement 2 - source data 5/Original files/Figure 1 - figure supplement 2 - source data 5 - highlighted/PTPRK total - highlighted.tif]

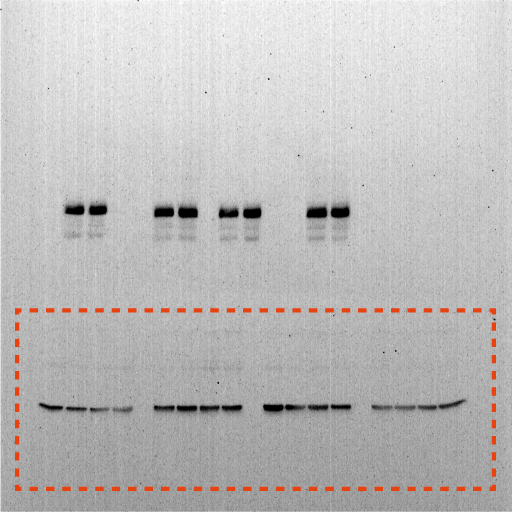

Supplement: Figure 1—figure supplement 2—source data 5. [file elife-79855-fig1-figsupp2-data5.zip › Figure 1 - figure supplement 2 - source data 5/Original files/Figure 1 - figure supplement 2 - source data 5 - highlighted/Tubulin - highlighted.tif]

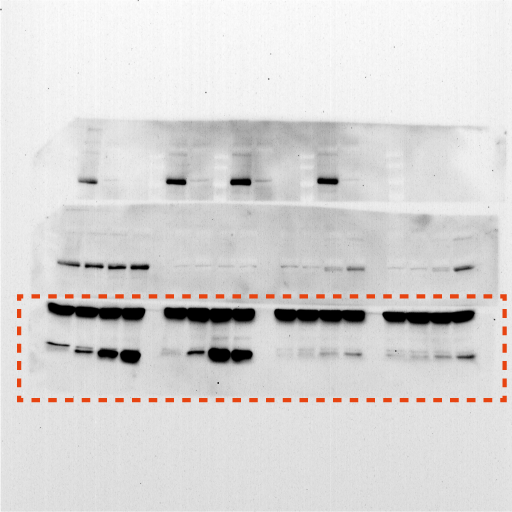

Supplement: Figure 1—figure supplement 2—source data 5. [file elife-79855-fig1-figsupp2-data5.zip › Figure 1 - figure supplement 2 - source data 5/Original files/Figure 1 - figure supplement 2 - source data 5 - highlighted/Turbo-GFP - highlighted.tif]

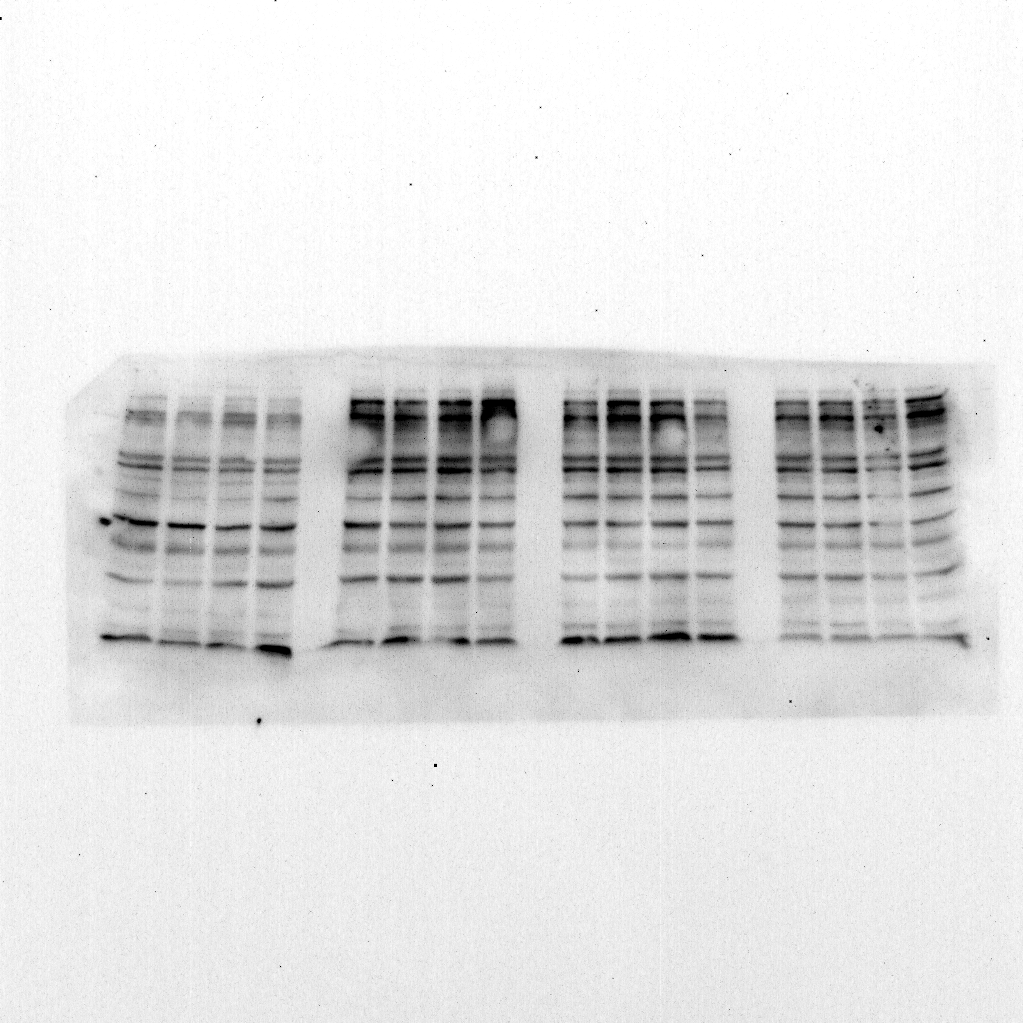

Supplement: Figure 1—figure supplement 2—source data 5. [file elife-79855-fig1-figsupp2-data5.zip › Figure 1 - figure supplement 2 - source data 5/Original files/Figure 1 - figure supplement 2 - source data 5 - unmodified/Afadin pY1230 high exp.tif]

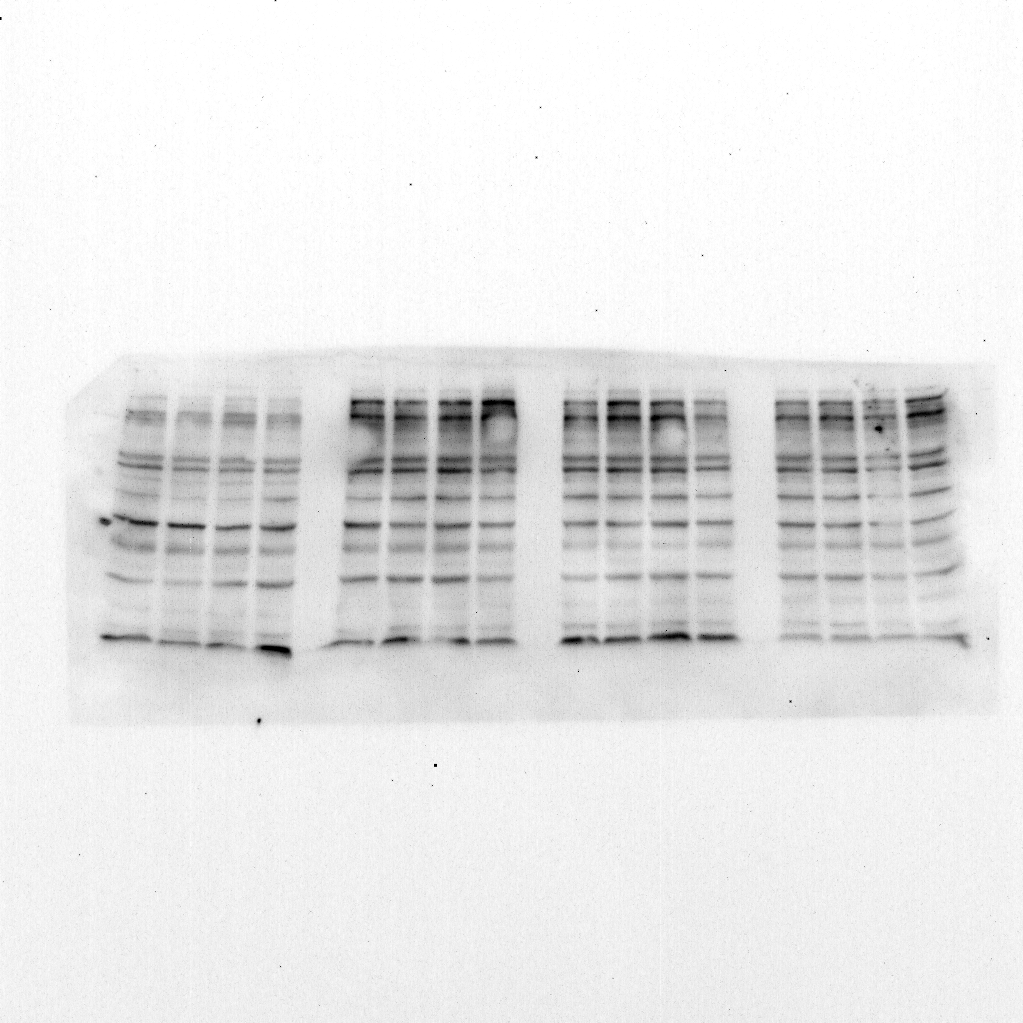

Supplement: Figure 1—figure supplement 2—source data 5. [file elife-79855-fig1-figsupp2-data5.zip › Figure 1 - figure supplement 2 - source data 5/Original files/Figure 1 - figure supplement 2 - source data 5 - unmodified/Afadin pY1230 low exp.tif]

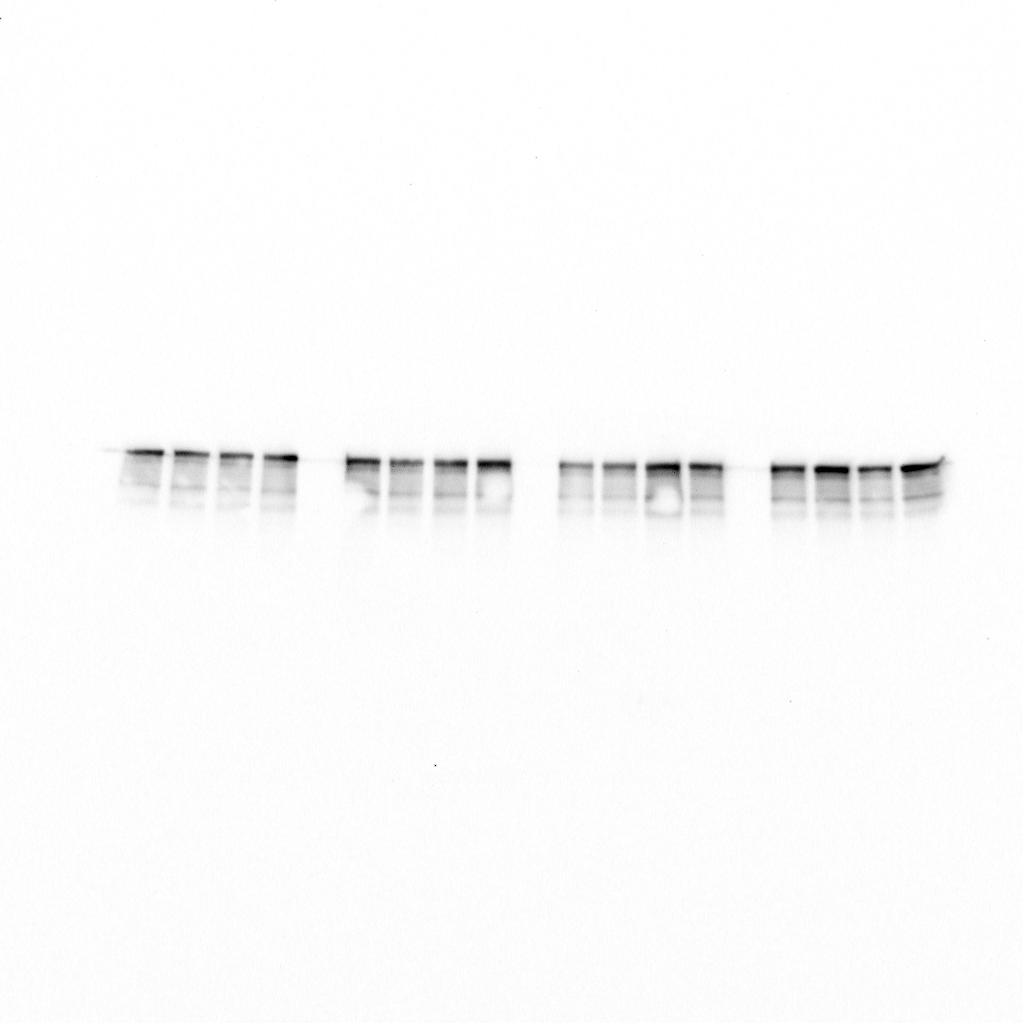

Supplement: Figure 1—figure supplement 2—source data 5. [file elife-79855-fig1-figsupp2-data5.zip › Figure 1 - figure supplement 2 - source data 5/Original files/Figure 1 - figure supplement 2 - source data 5 - unmodified/Afadin total.tif]

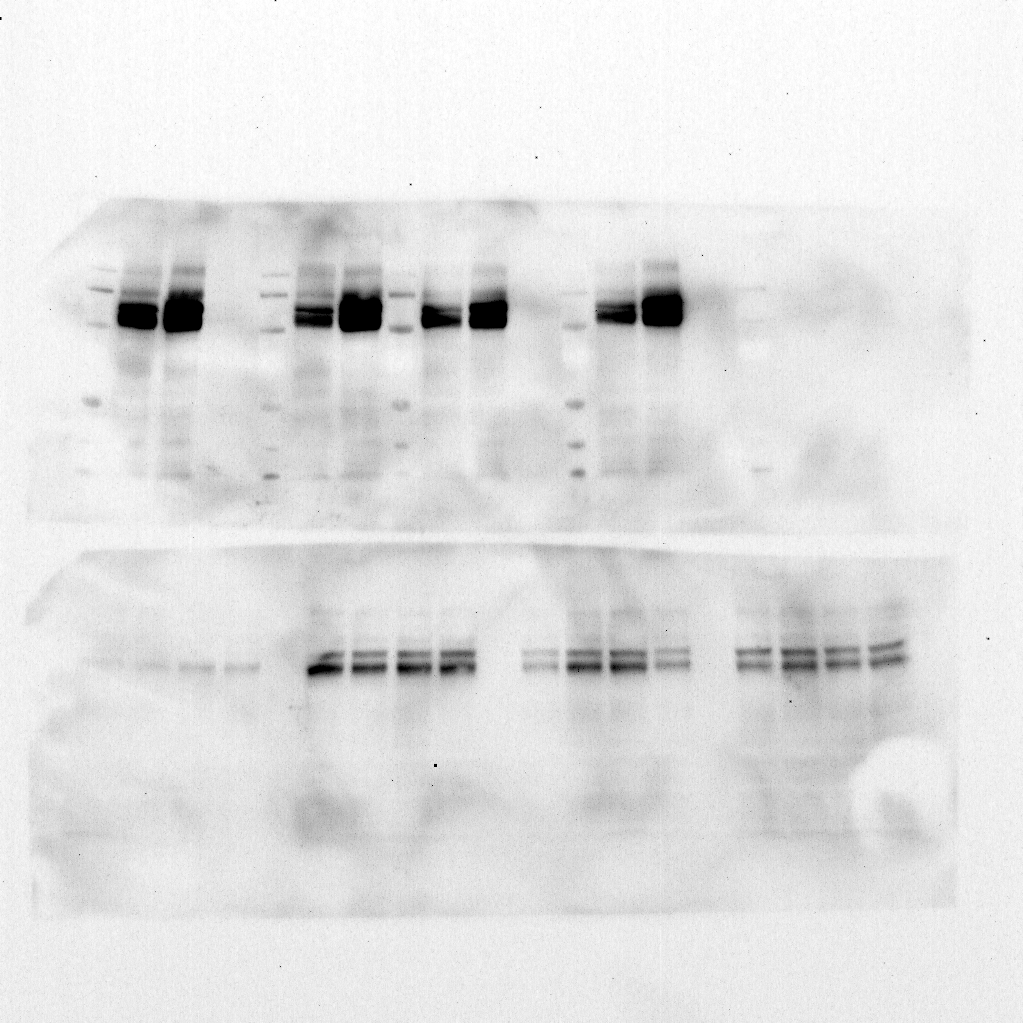

Supplement: Figure 1—figure supplement 2—source data 5. [file elife-79855-fig1-figsupp2-data5.zip › Figure 1 - figure supplement 2 - source data 5/Original files/Figure 1 - figure supplement 2 - source data 5 - unmodified/p120 catenin pY228 - bottom blot.tif]

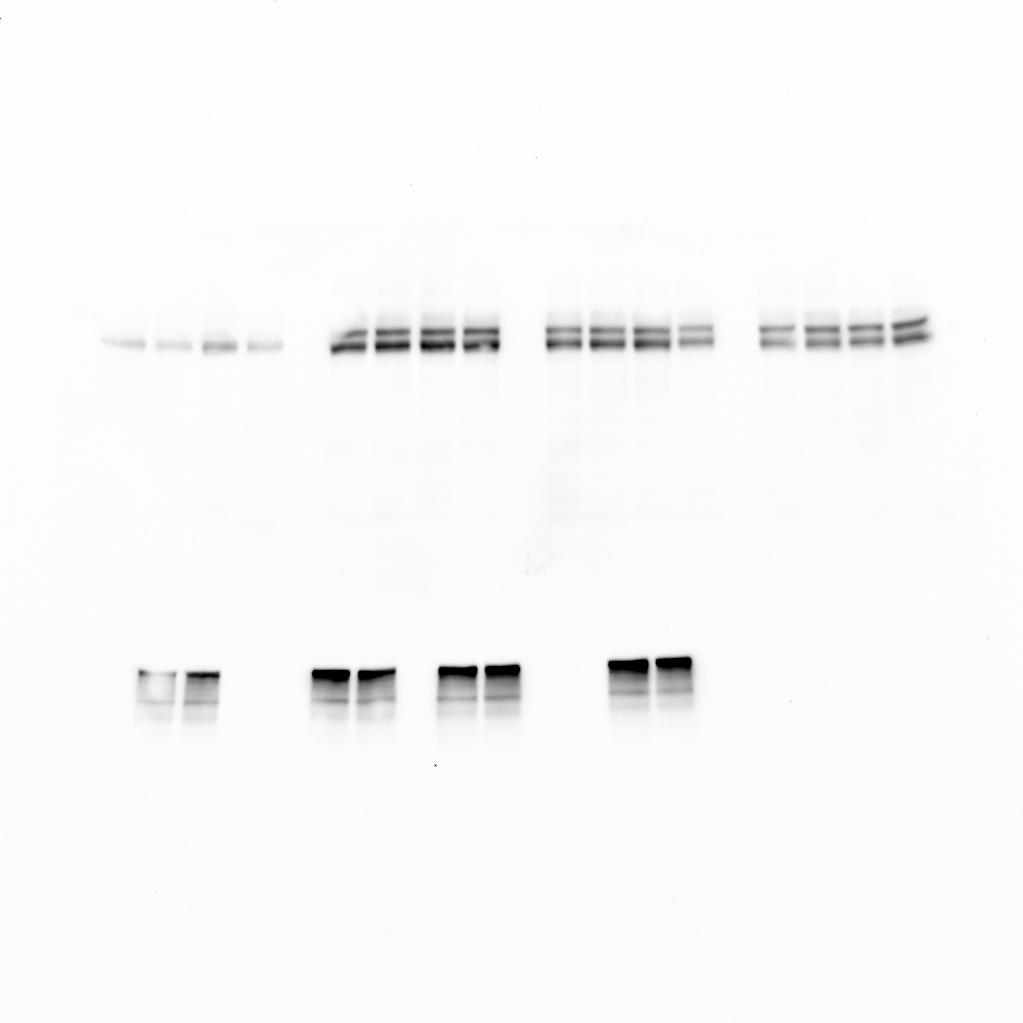

Supplement: Figure 1—figure supplement 2—source data 5. [file elife-79855-fig1-figsupp2-data5.zip › Figure 1 - figure supplement 2 - source data 5/Original files/Figure 1 - figure supplement 2 - source data 5 - unmodified/p120 catenin pY904 - top blot.tif]

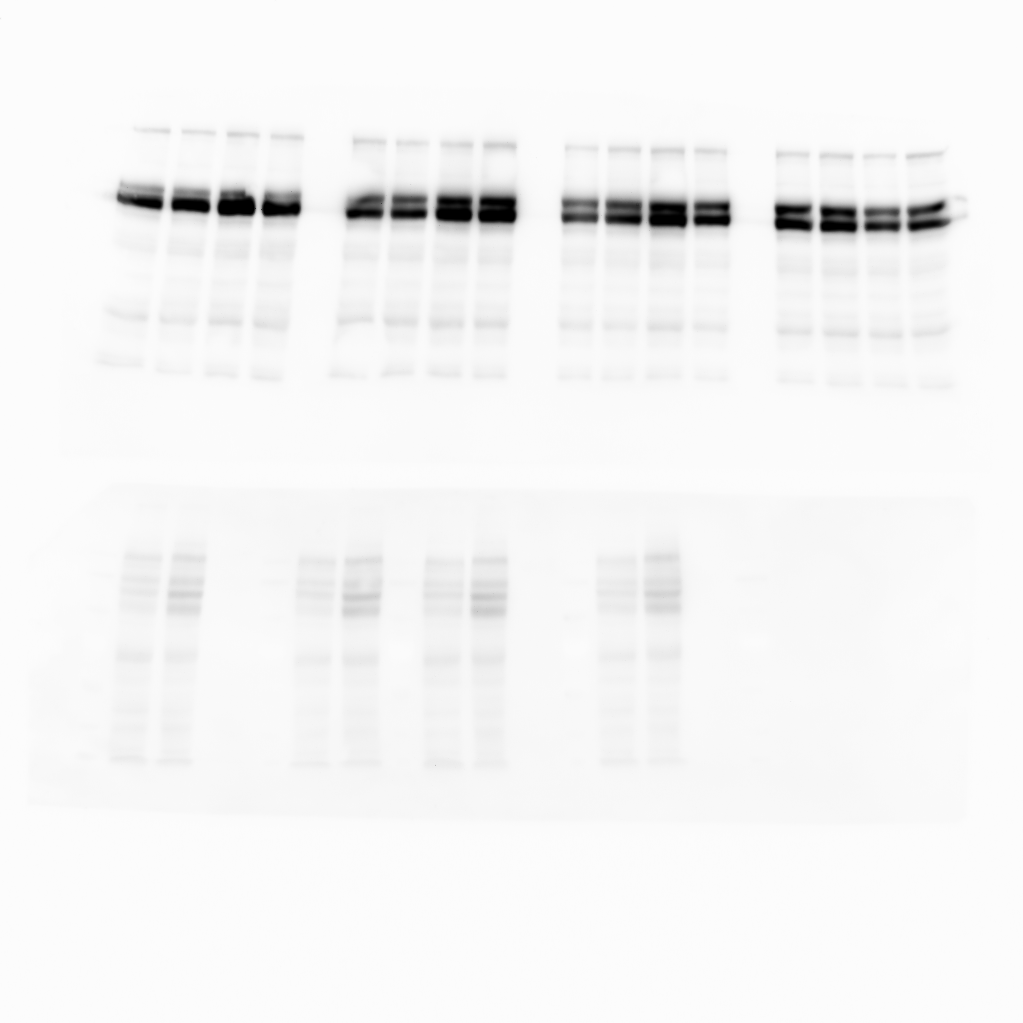

Supplement: Figure 1—figure supplement 2—source data 5. [file elife-79855-fig1-figsupp2-data5.zip › Figure 1 - figure supplement 2 - source data 5/Original files/Figure 1 - figure supplement 2 - source data 5 - unmodified/p120 catenin total - top blot.tif]

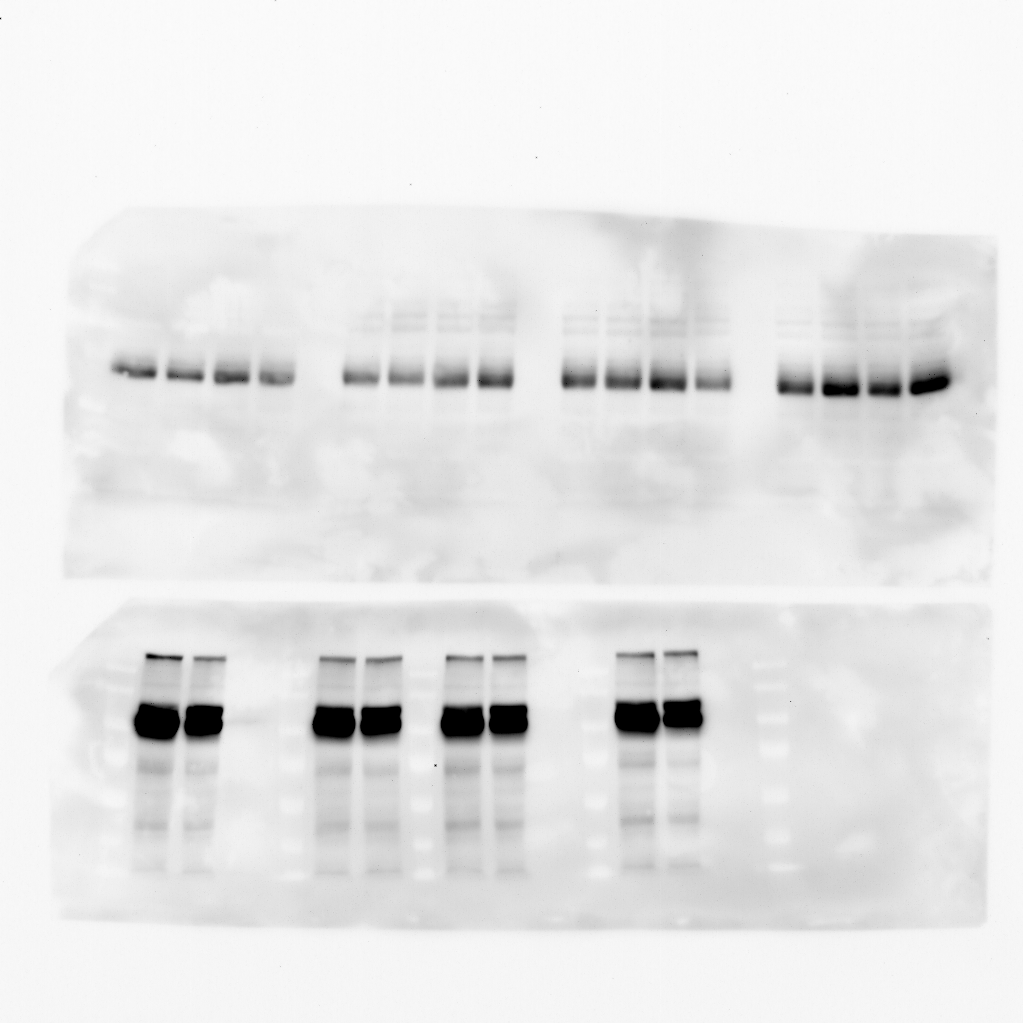

Supplement: Figure 1—figure supplement 2—source data 5. [file elife-79855-fig1-figsupp2-data5.zip › Figure 1 - figure supplement 2 - source data 5/Original files/Figure 1 - figure supplement 2 - source data 5 - unmodified/Paxillin pY118 - top blot.tif]

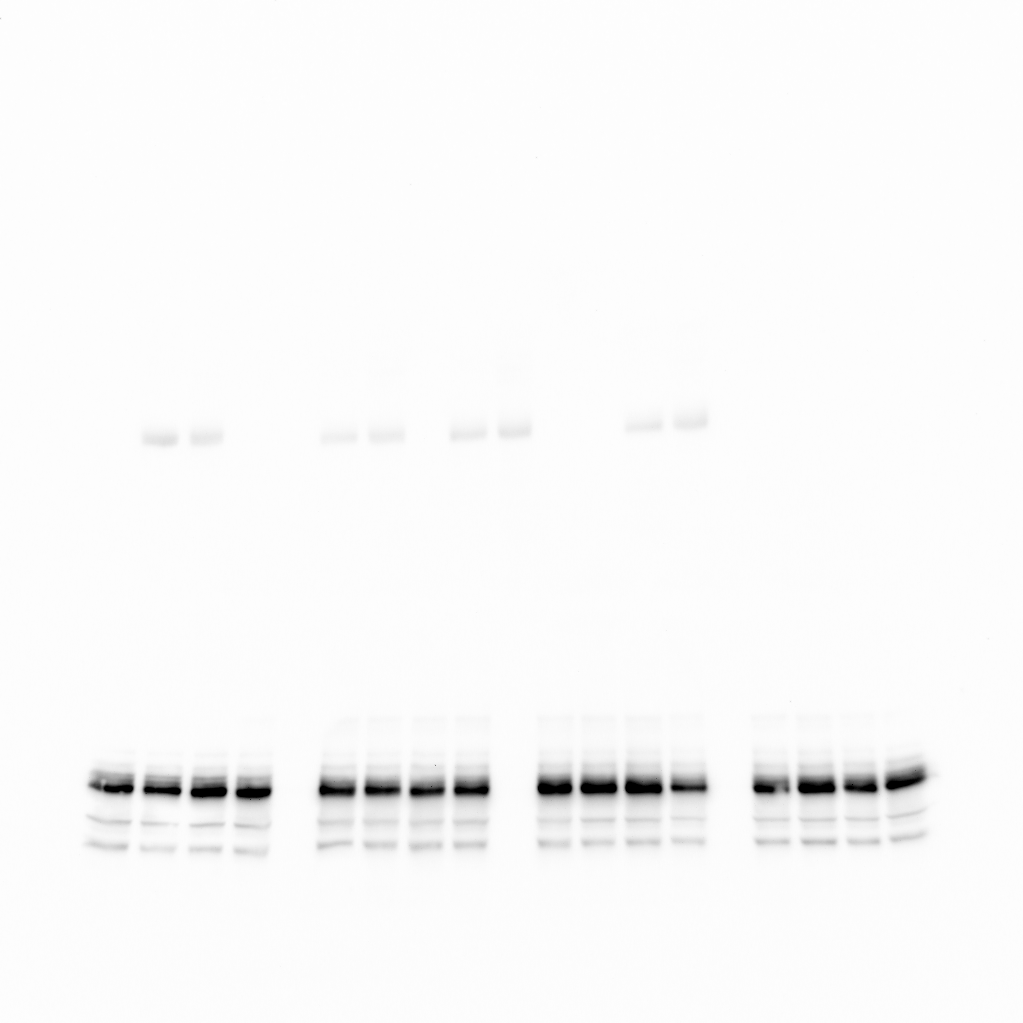

Supplement: Figure 1—figure supplement 2—source data 5. [file elife-79855-fig1-figsupp2-data5.zip › Figure 1 - figure supplement 2 - source data 5/Original files/Figure 1 - figure supplement 2 - source data 5 - unmodified/Paxillin total - bottom blot.tif]

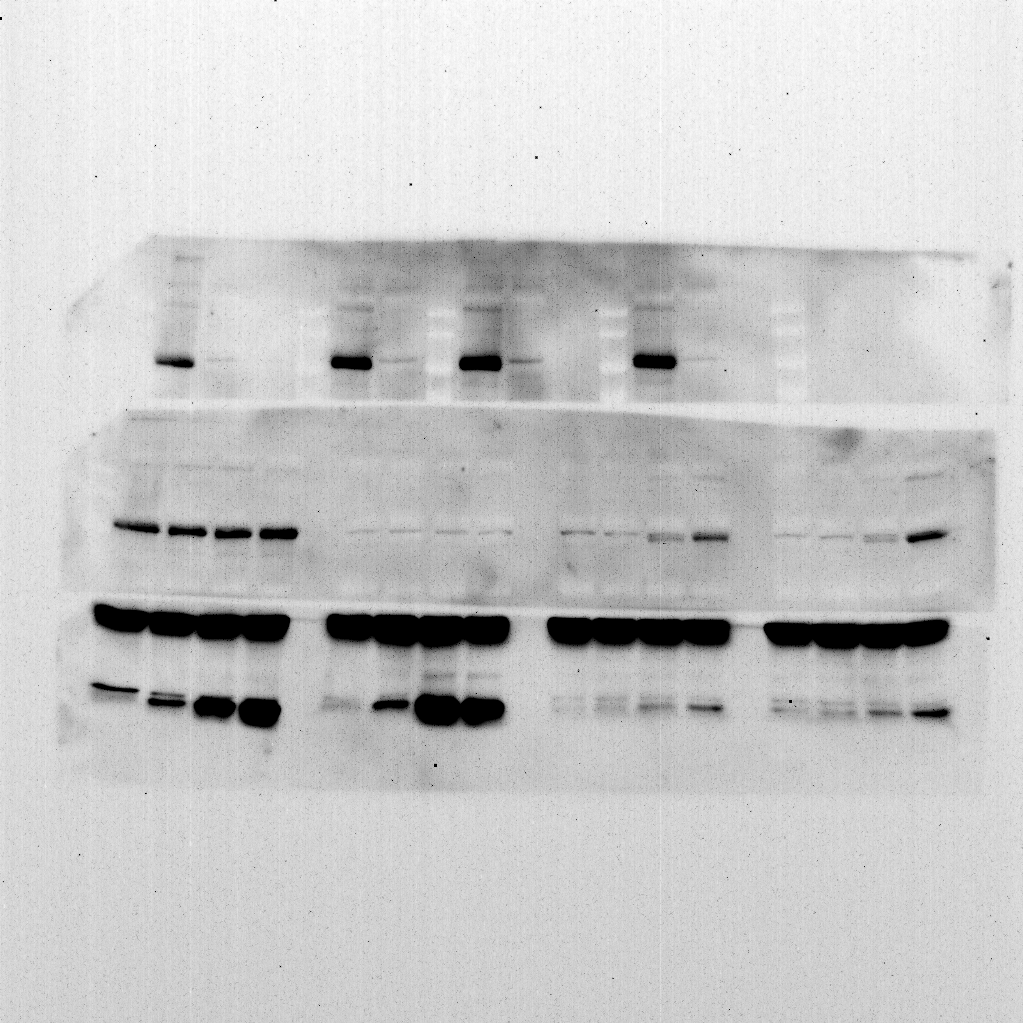

Supplement: Figure 1—figure supplement 2—source data 5. [file elife-79855-fig1-figsupp2-data5.zip › Figure 1 - figure supplement 2 - source data 5/Original files/Figure 1 - figure supplement 2 - source data 5 - unmodified/PTPRK total - middle blot.tif]

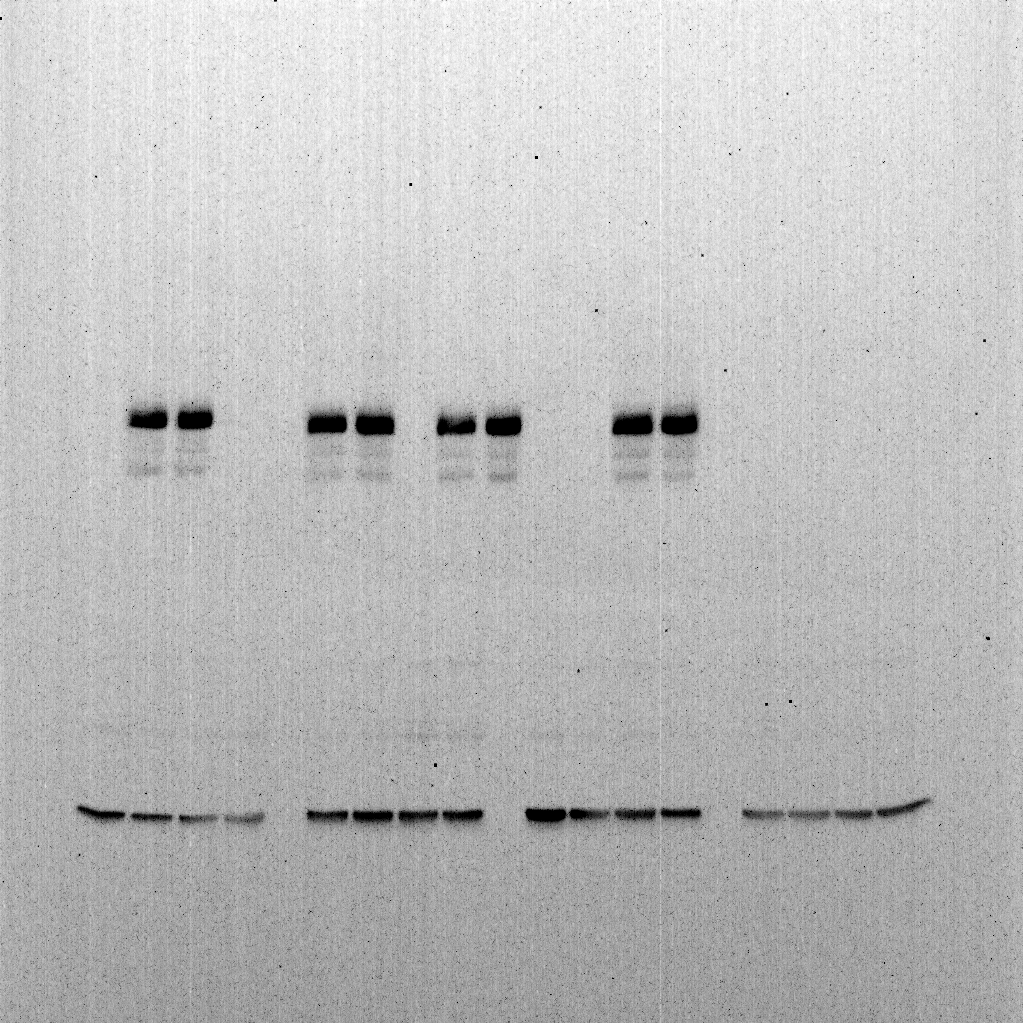

Supplement: Figure 1—figure supplement 2—source data 5. [file elife-79855-fig1-figsupp2-data5.zip › Figure 1 - figure supplement 2 - source data 5/Original files/Figure 1 - figure supplement 2 - source data 5 - unmodified/Tubulin - bottom blot.tif]

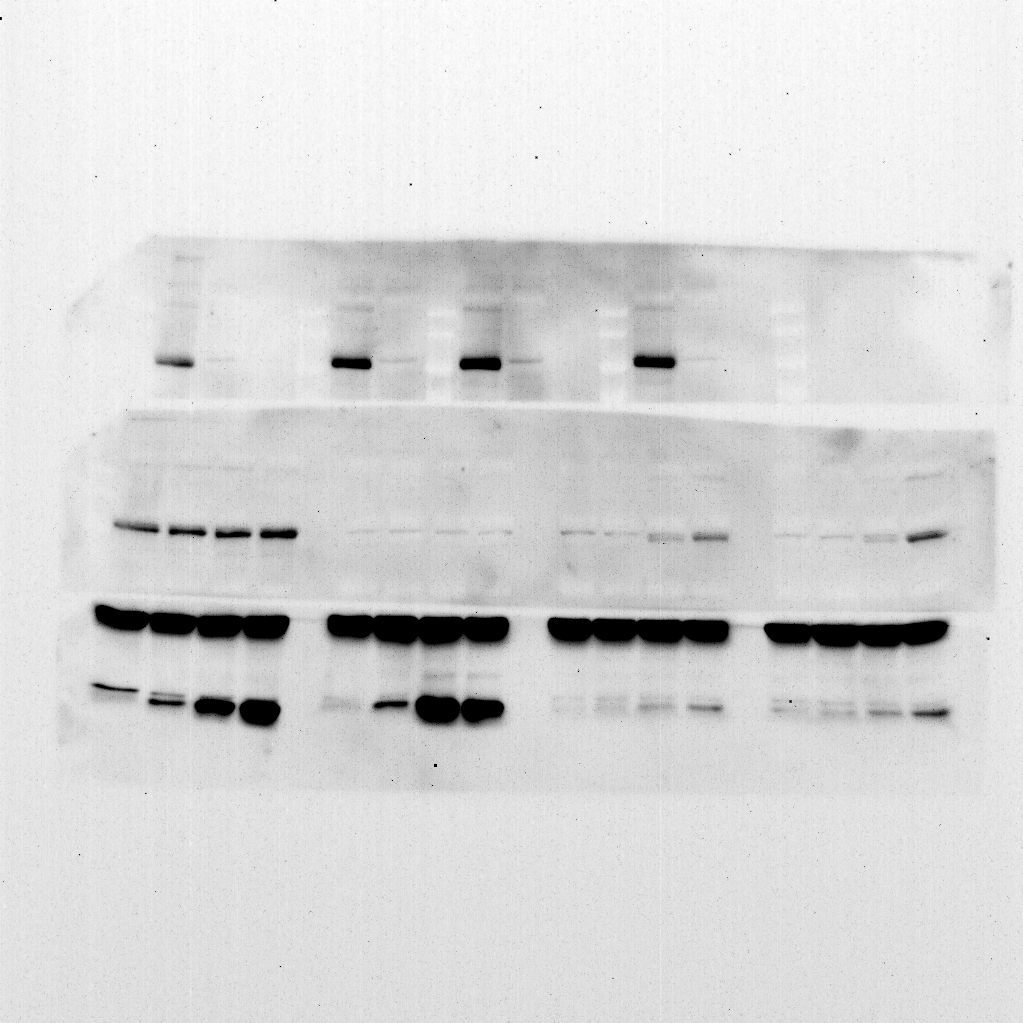

Supplement: Figure 1—figure supplement 2—source data 5. [file elife-79855-fig1-figsupp2-data5.zip › Figure 1 - figure supplement 2 - source data 5/Original files/Figure 1 - figure supplement 2 - source data 5 - unmodified/Turbo-GFP - bottom blot.tif]

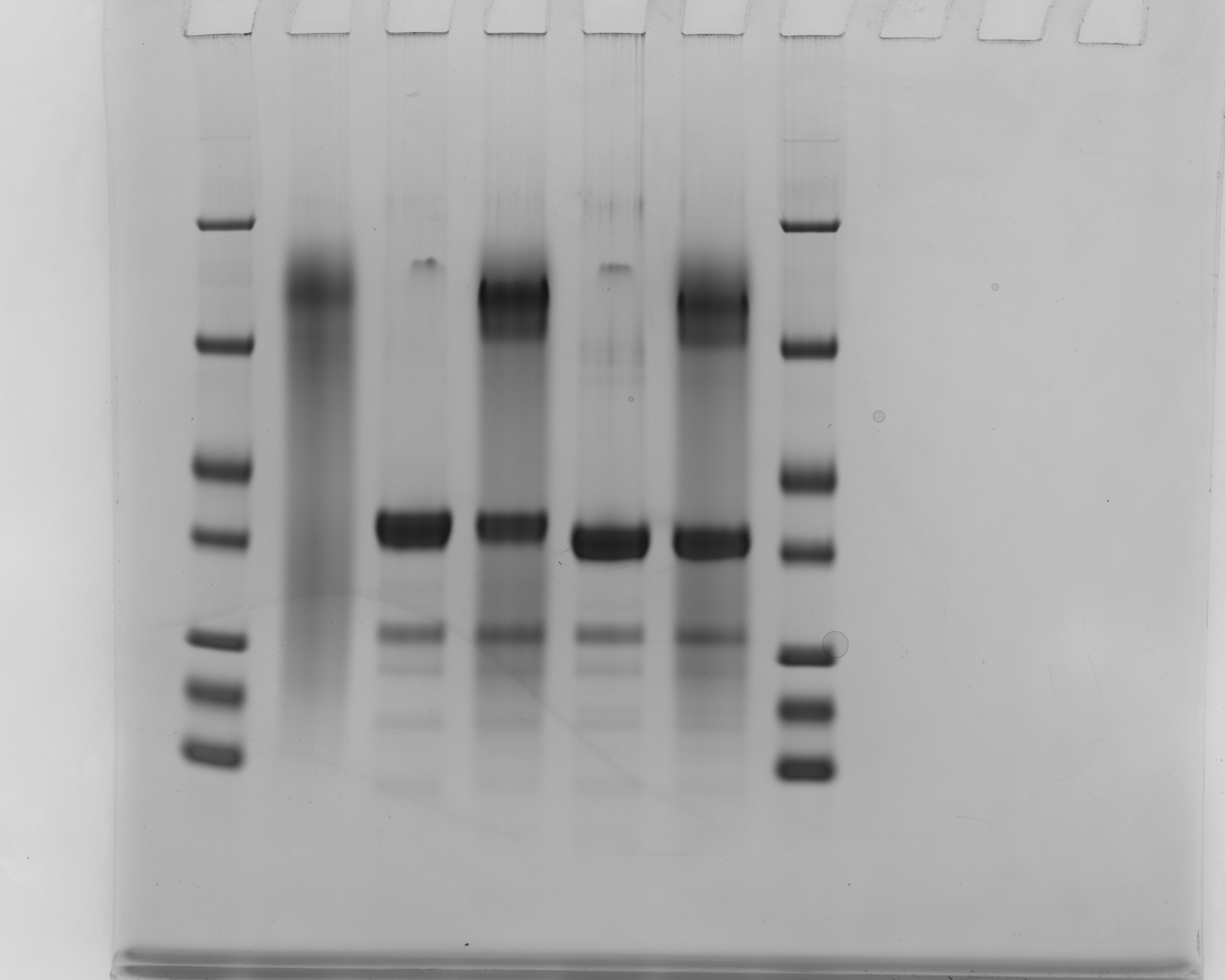

Supplement: Figure 1—figure supplement 3—source data 1. [file elife-79855-fig1-figsupp3-data1.zip › Figure 1 - figure supplement 3 - source data 1/Original files/F1_S3A.tif]

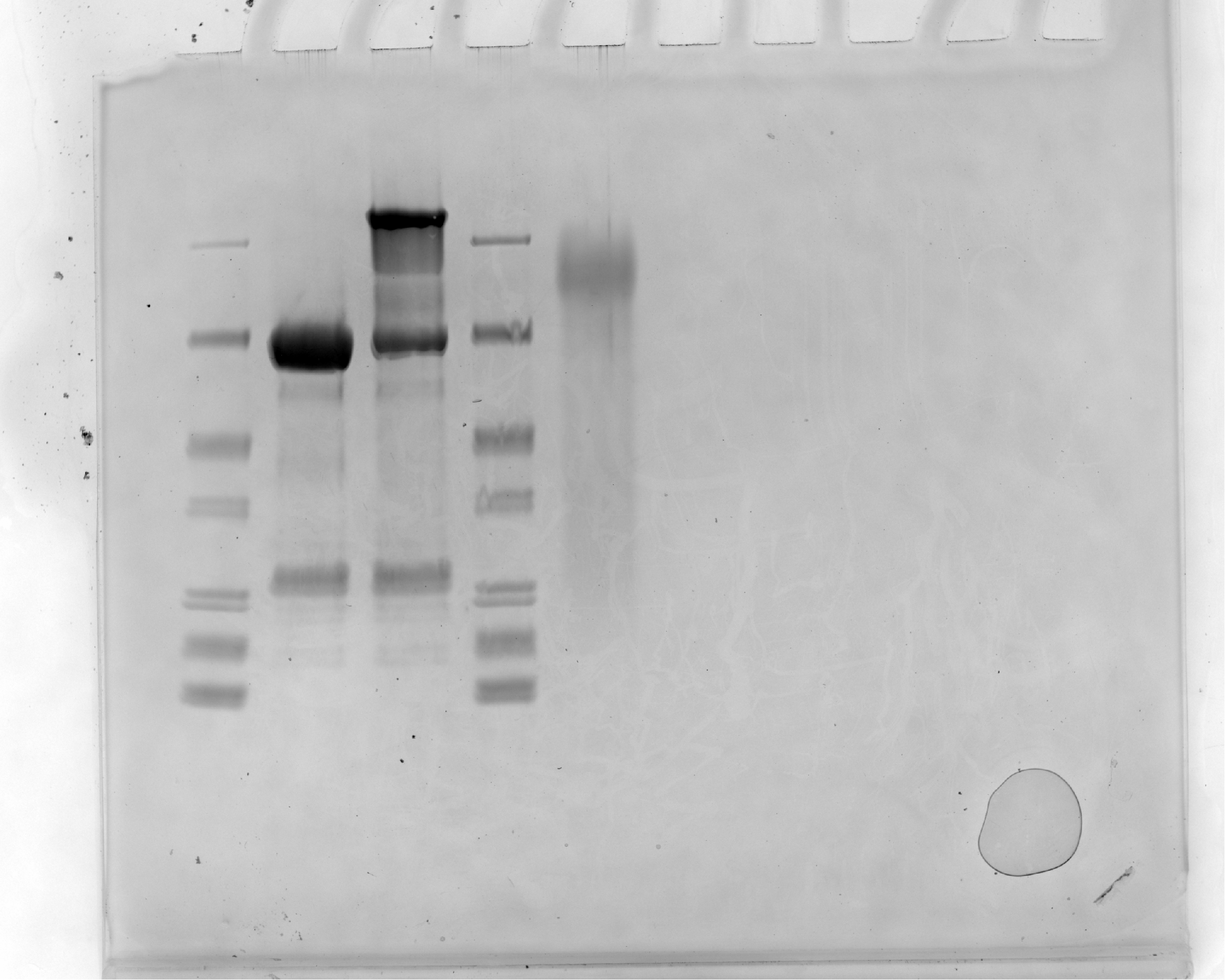

Supplement: Figure 1—figure supplement 3—source data 2. [file elife-79855-fig1-figsupp3-data2.zip › Figure 1 - figure supplement 3 - source data 2/Original files/F1_S3B.tif]

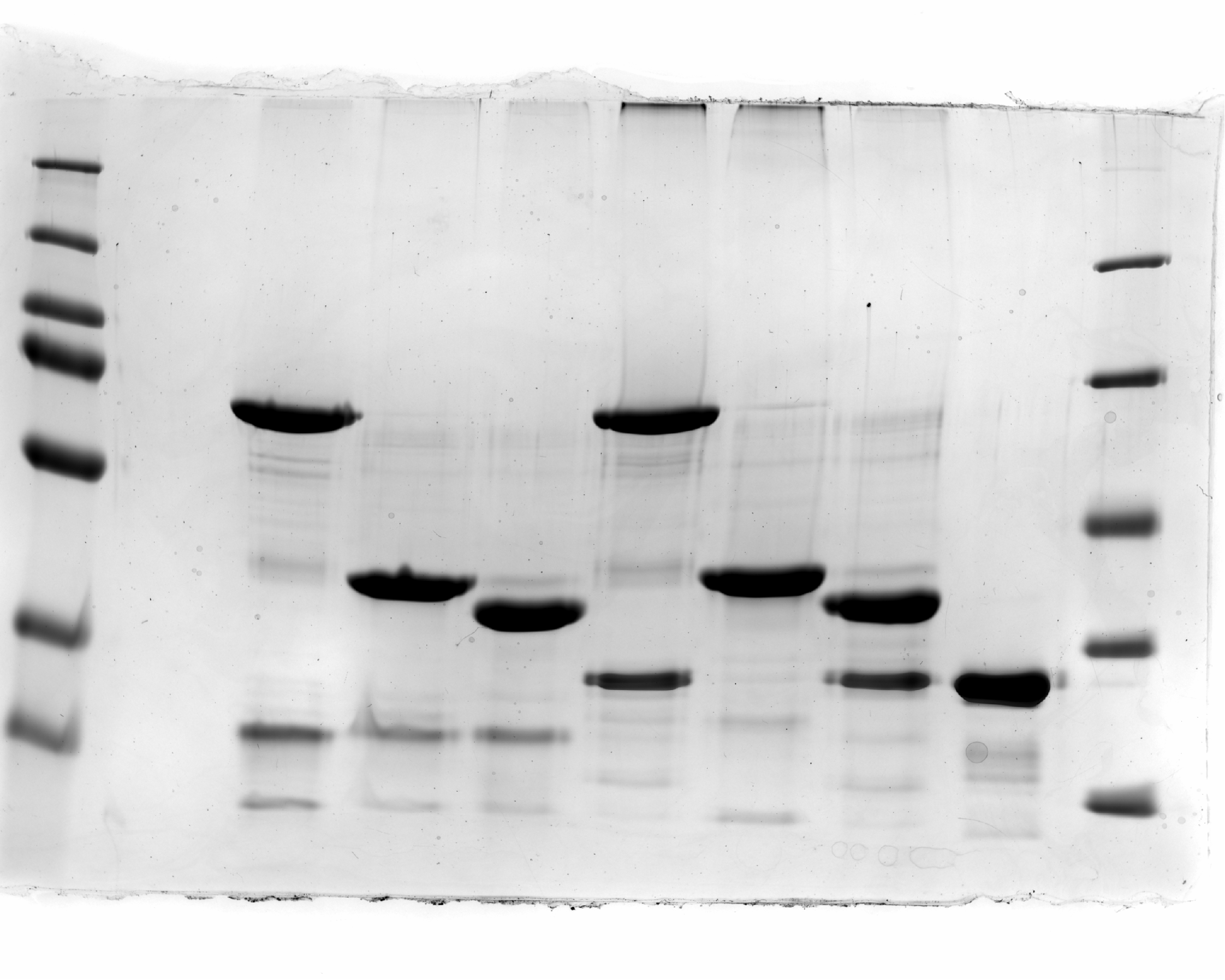

Supplement: Figure 2—source data 1. [file elife-79855-fig2-data1.zip › Figure 2 - source data 1/Original files/2C.tif]

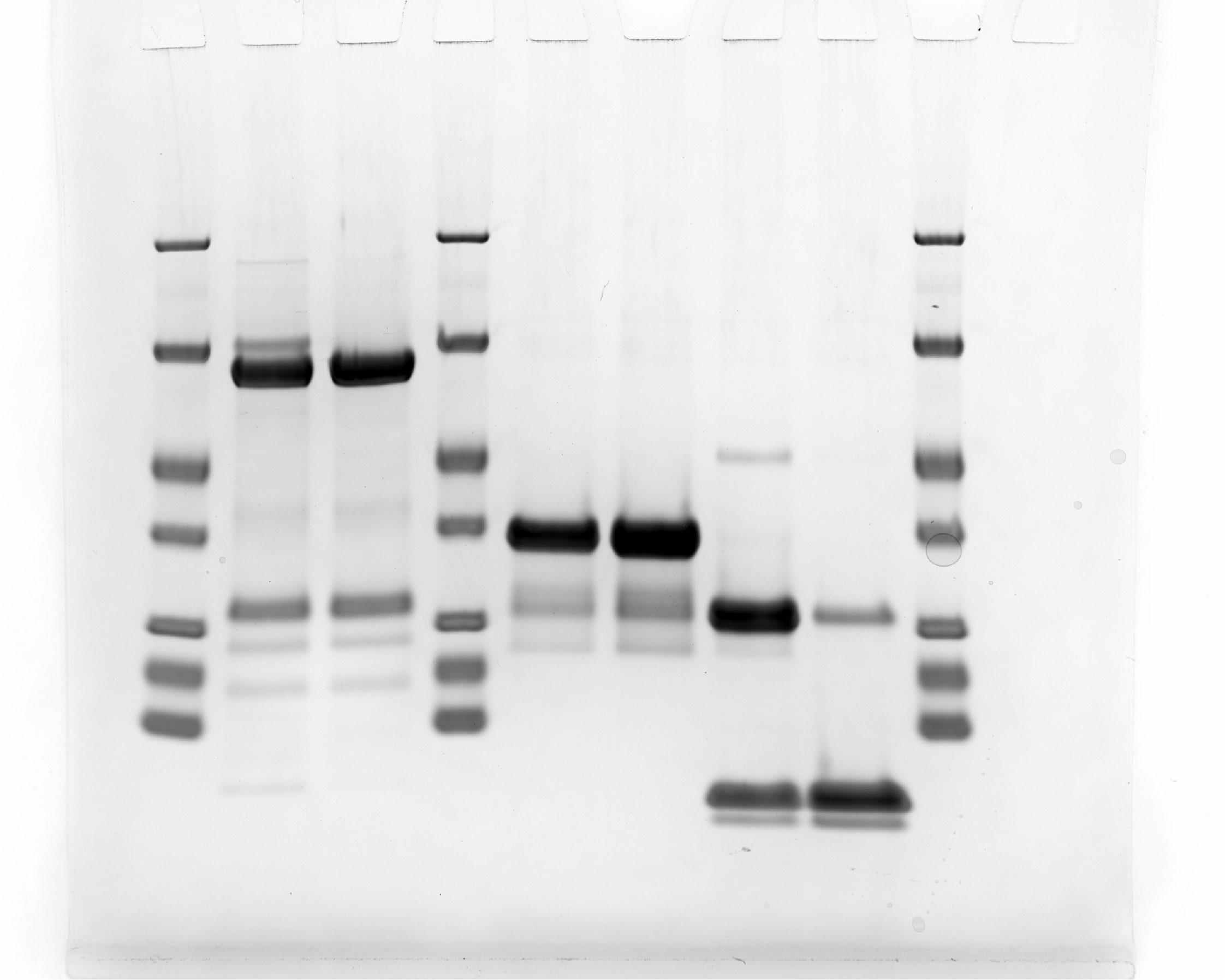

Supplement: Figure 2—figure supplement 1—source data 1. [file elife-79855-fig2-figsupp1-data1.zip › Figure 2 - figure supplement 1 - source data 1/Original files/F2_S1A.tif]

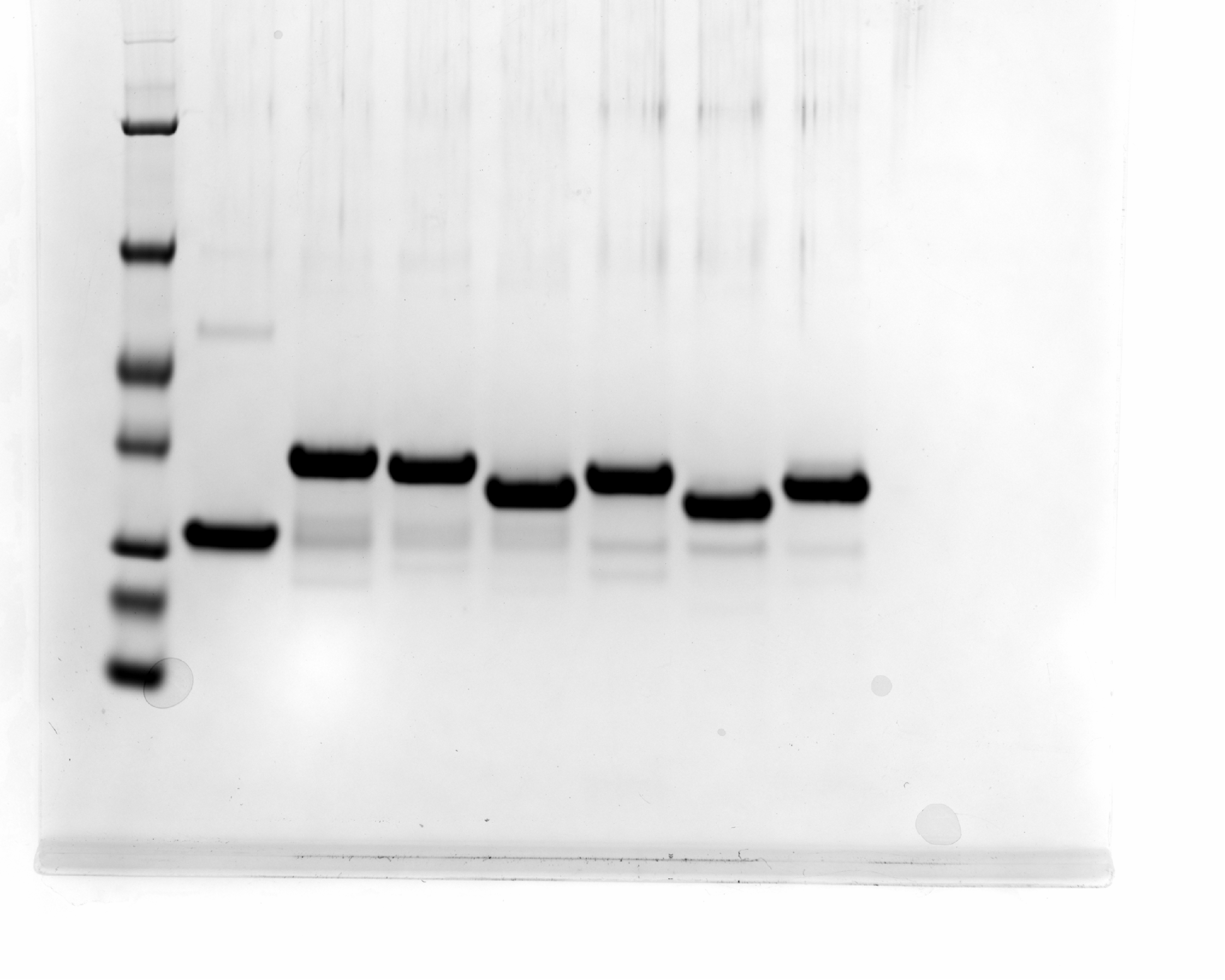

Supplement: Figure 3—source data 1. [file elife-79855-fig3-data1.zip › Figure 3 - source data 1/Original files/3A_inputs.tif]

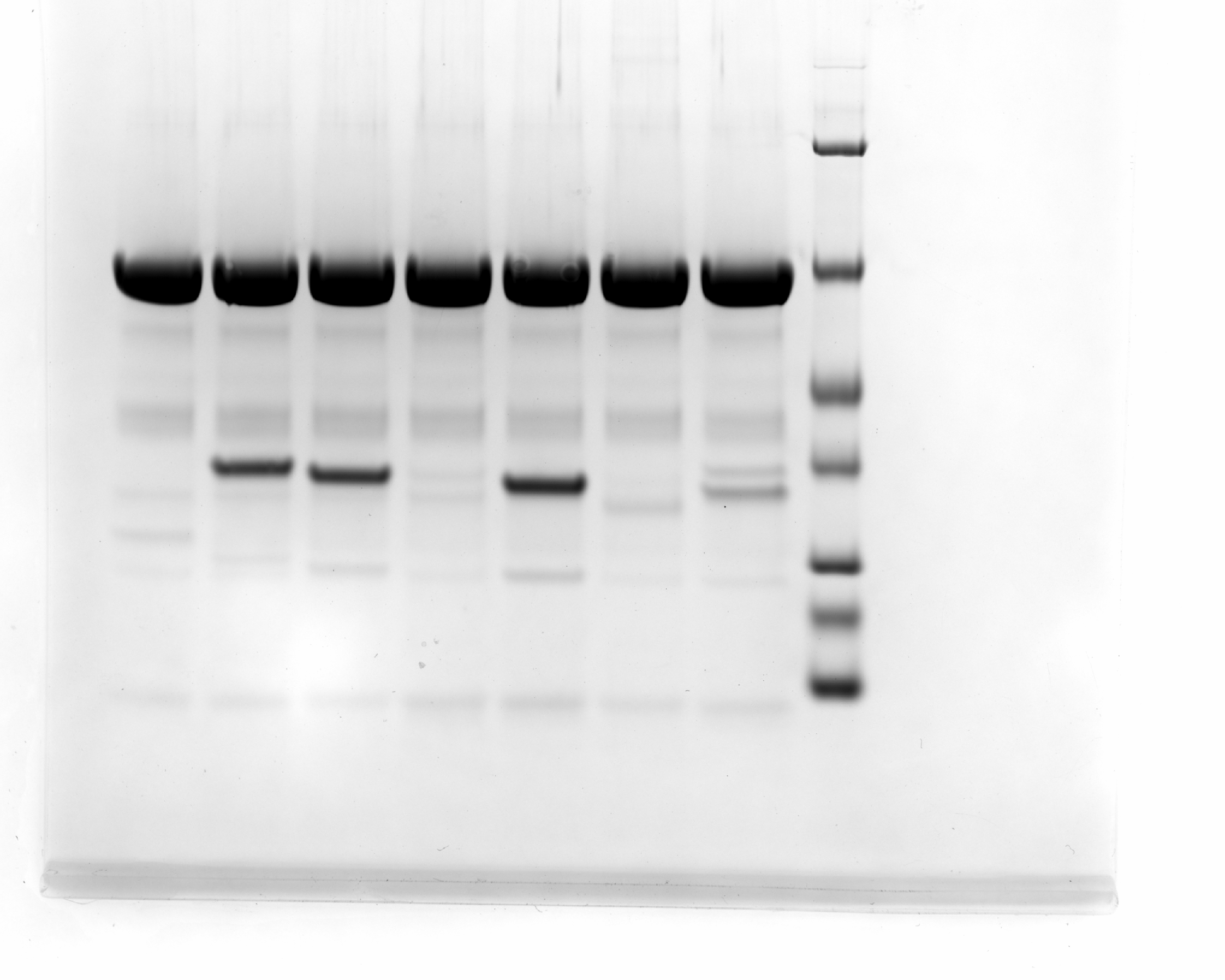

Supplement: Figure 3—source data 1. [file elife-79855-fig3-data1.zip › Figure 3 - source data 1/Original files/3A_pull-downs.tif]

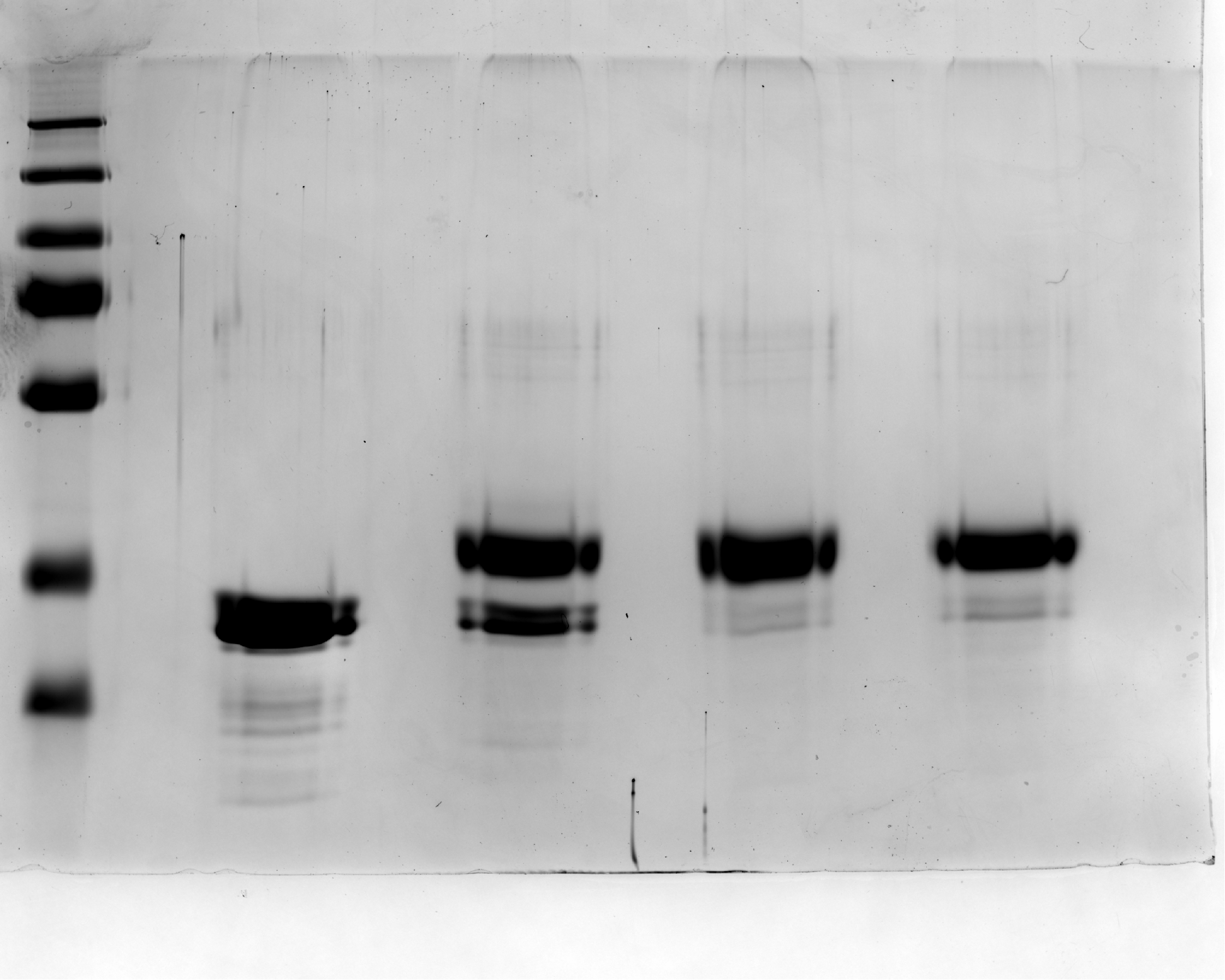

Supplement: Figure 4—source data 1. [file elife-79855-fig4-data1.zip › Figure 4 - source data 1/Original files/4D.tif]

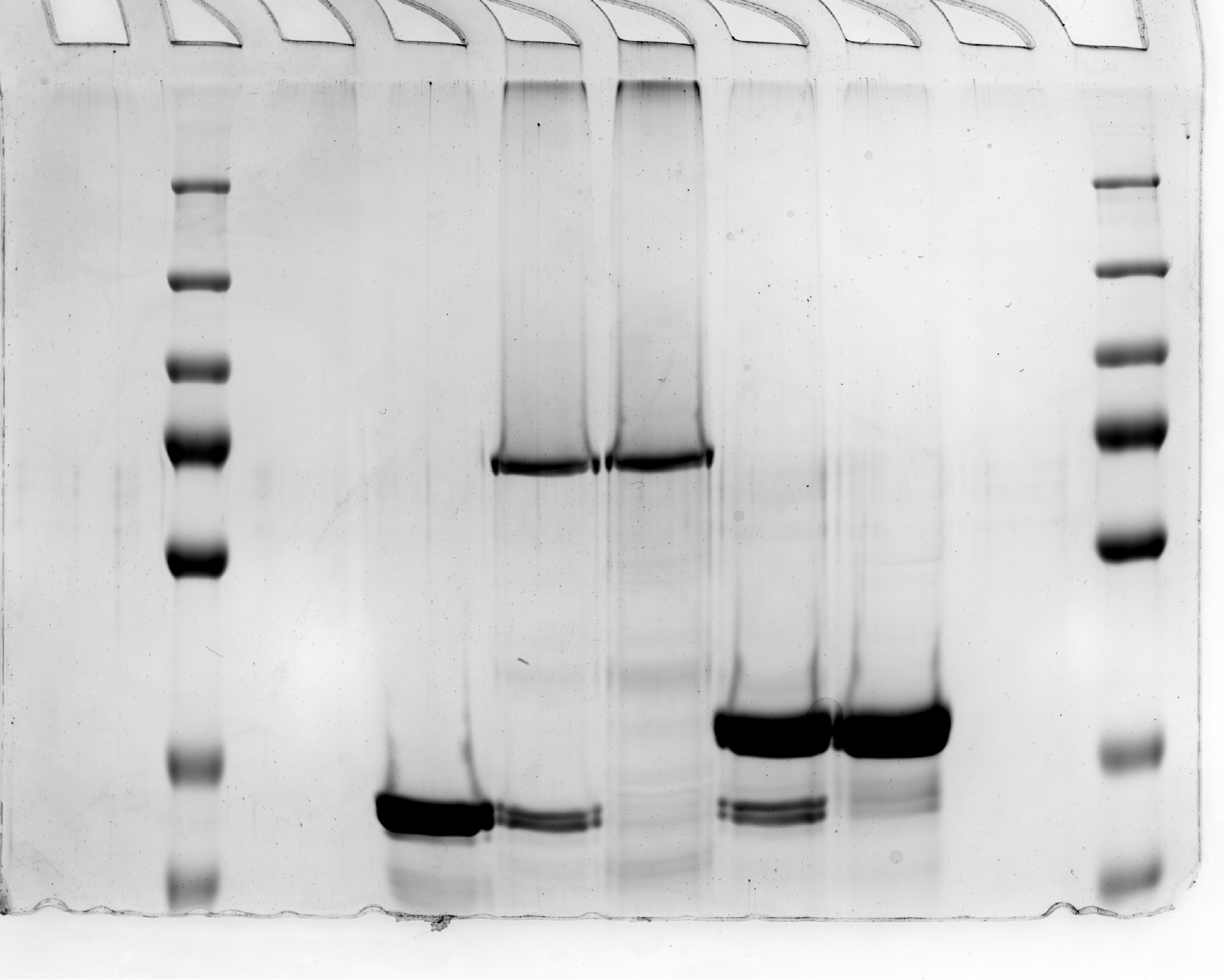

Supplement: Figure 4—source data 2. [file elife-79855-fig4-data2.zip › Figure 4 - source data 2/Original files/4E.tif]

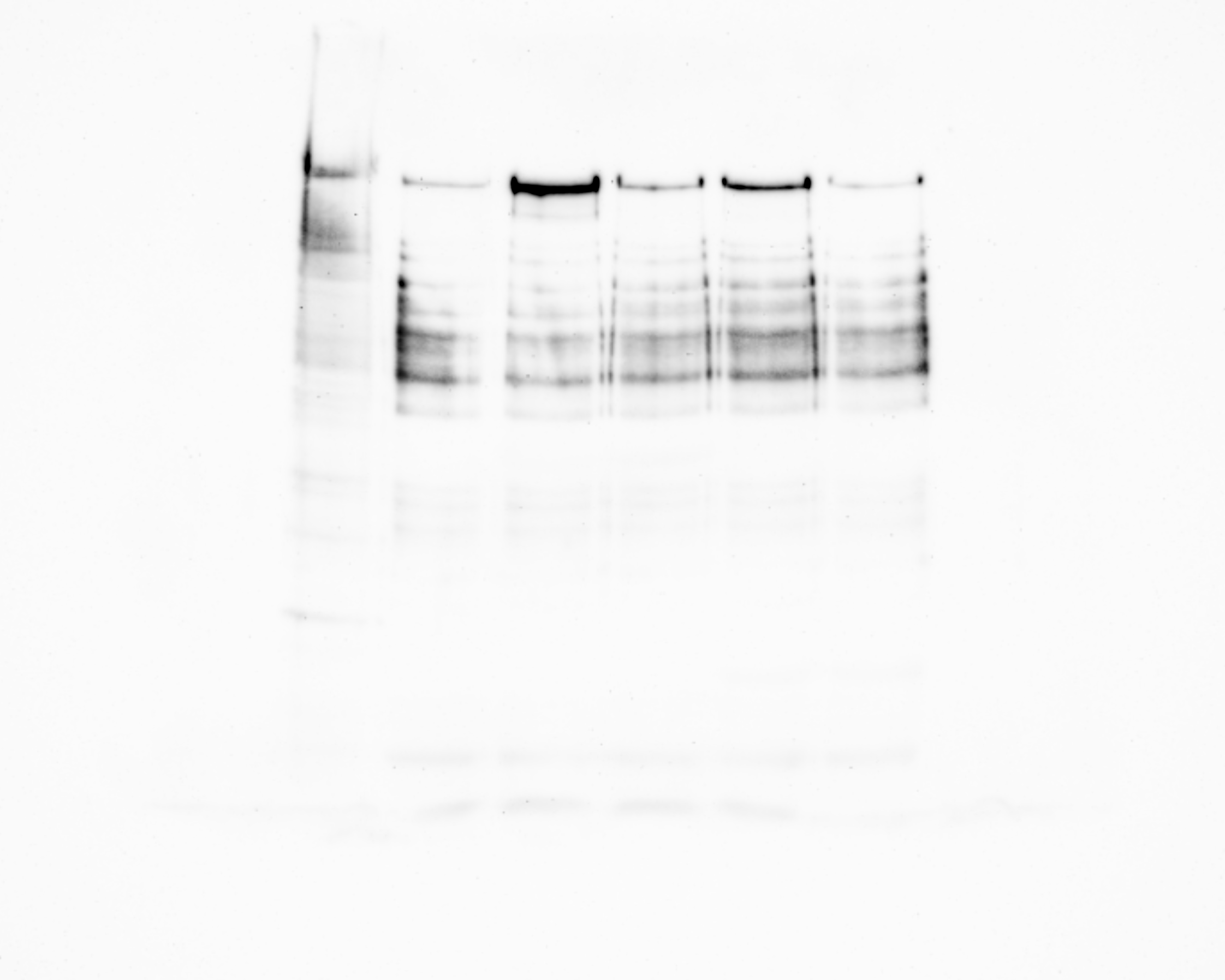

Supplement: Figure 4—source data 3. [file elife-79855-fig4-data3.zip › Figure 4 - source data 3/Original files/4G_Myctag.tif]

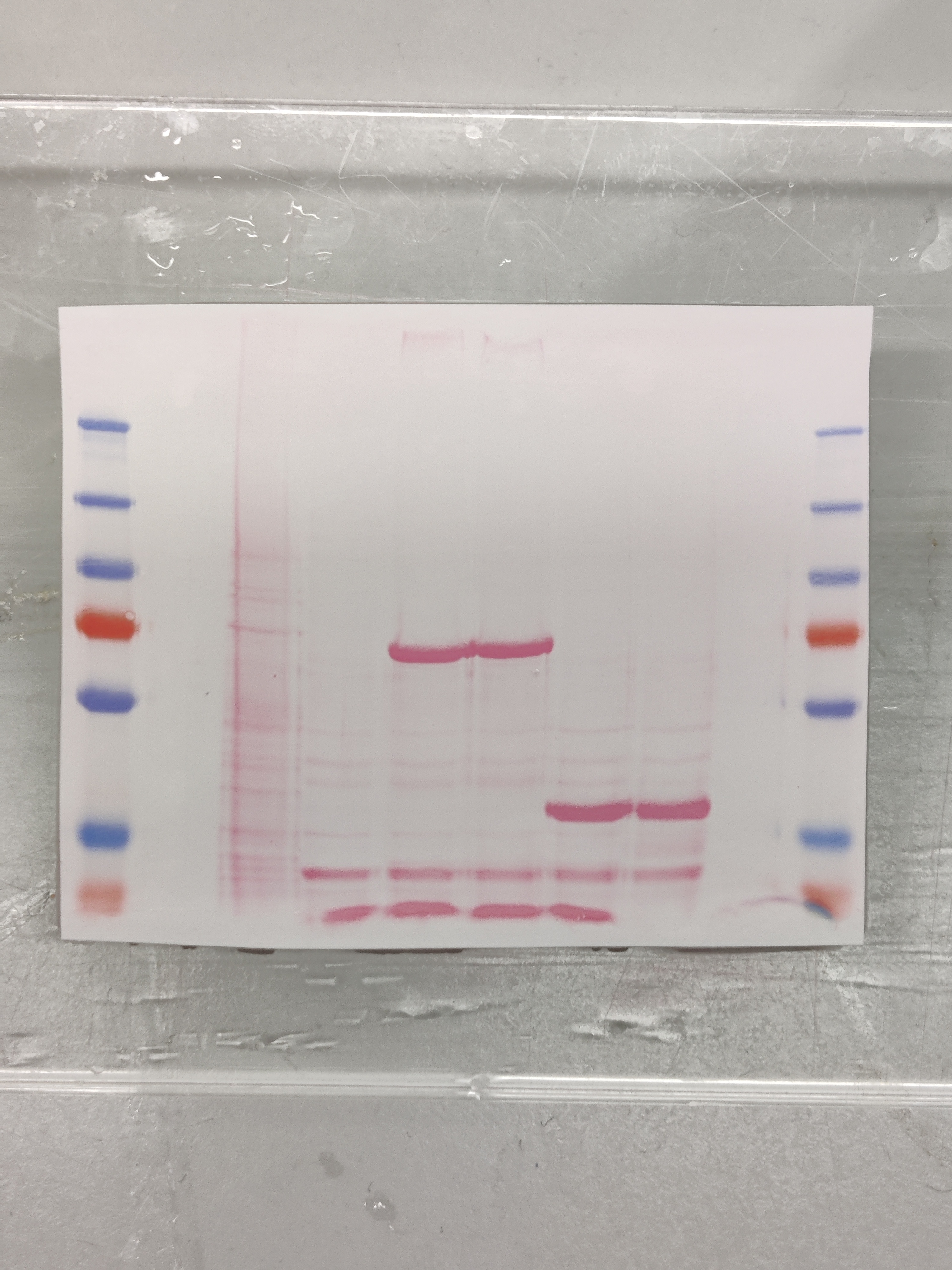

Supplement: Figure 4—source data 3. [file elife-79855-fig4-data3.zip › Figure 4 - source data 3/Original files/4G_ponceau.jpg]

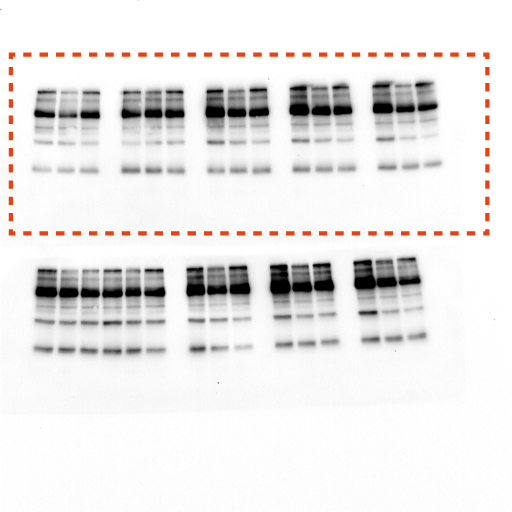

Supplement: Figure 5—source data 1. [file elife-79855-fig5-data1.zip › Figure 5 – source data 1/Original files/Figure 5 - source data 1 - highlighted/Afadin pY1230 - highlighted.tif]

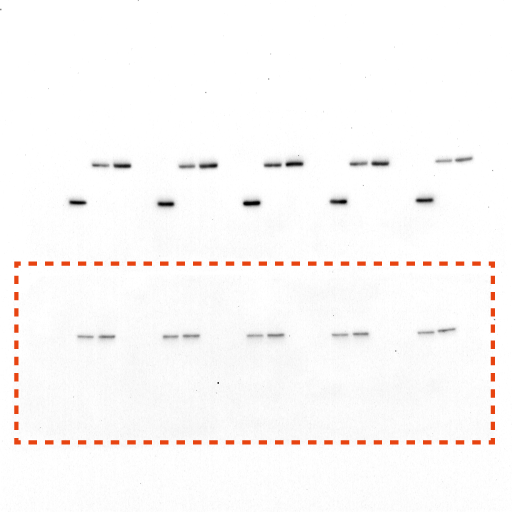

Supplement: Figure 5—source data 1. [file elife-79855-fig5-data1.zip › Figure 5 – source data 1/Original files/Figure 5 - source data 1 - highlighted/His totals - highlighted.tif]

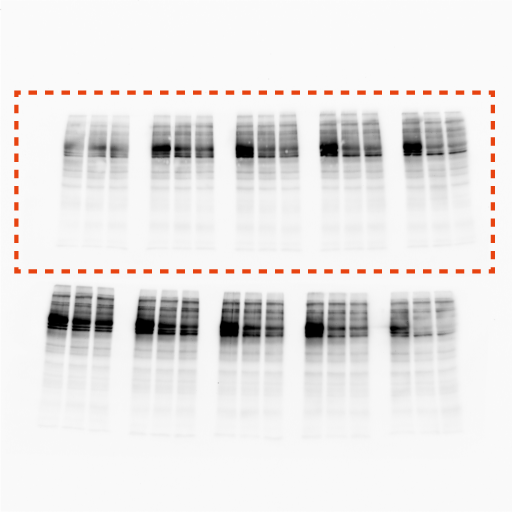

Supplement: Figure 5—source data 1. [file elife-79855-fig5-data1.zip › Figure 5 – source data 1/Original files/Figure 5 - source data 1 - highlighted/p120 catenin pY228 - highlighted.tif]

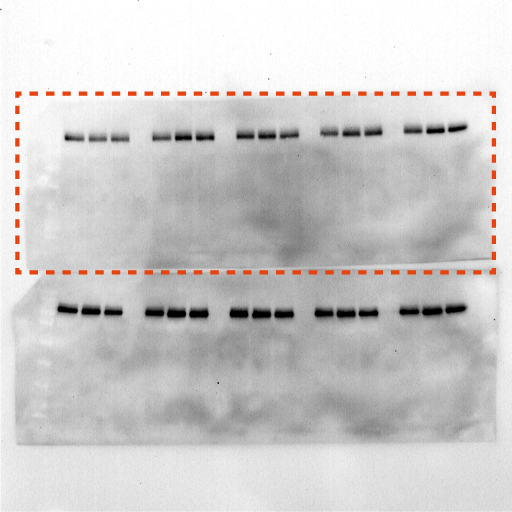

Supplement: Figure 5—source data 1. [file elife-79855-fig5-data1.zip › Figure 5 – source data 1/Original files/Figure 5 - source data 1 - highlighted/p120 catenin total - highlighted.tif]

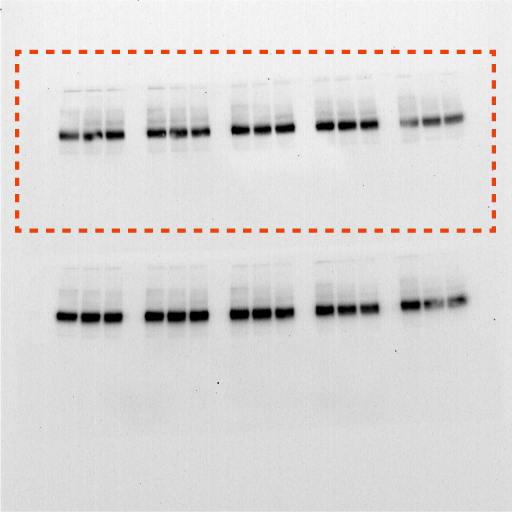

Supplement: Figure 5—source data 1. [file elife-79855-fig5-data1.zip › Figure 5 – source data 1/Original files/Figure 5 - source data 1 - highlighted/Paxillin pY118 - highlighted.tif]

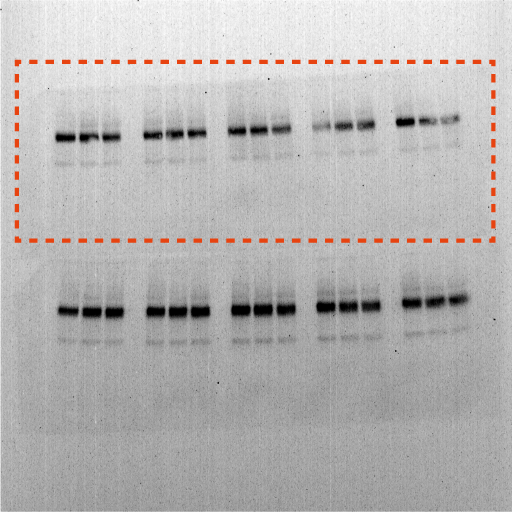

Supplement: Figure 5—source data 1. [file elife-79855-fig5-data1.zip › Figure 5 – source data 1/Original files/Figure 5 - source data 1 - highlighted/Paxillin total - highlighted.tif]
