# Supplementary material for: Molecular mechanism of Afadin substrate recruitment to the receptor phosphatase PTPRK via its pseudophosphatase domain
Source: eLife. 2022 Oct 20;11:e79855. doi: 10.7554/eLife.79855 (PMC9640194; doi:10.7554/eLife.79855)
Supplement: Figure 3—source data 2. [file elife-79855-fig3-data2.zip › Figure 3 – source data 2/Original files/PTPRK-Afadin CC point mutant pulldown 5.pdf]

Image ID: 0004597_06
Acquire Time: 19-Aug-2022 17:20:45

Acquisition Information

#	Image ID	Acquire Time	Channels	Resolution	Intensities	Quality	Analysis	Image Name	Comment
1	0004597_06	19-Aug-2022 17:20:45	700 800	169um	Auto Auto	high	Manual	0004597_06	

Image Display Values

Channel	Color	Minimum	Maximum	K
700	Gray Scale (Black on White)	0.183	38.9	0
800	Gray Scale (Black on White)	0.570	3.56	0

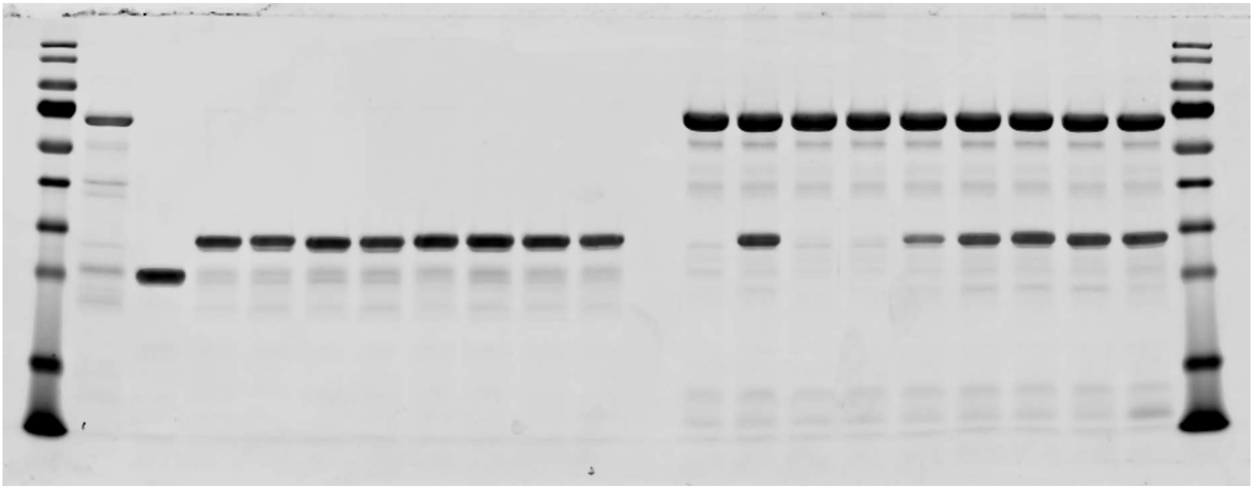

Acquisition Information (continued)

#	Image Modifications
1	Flip Top to Bottom Image ID: 0004597_01; Noise Removal Image ID: 0004597_05
