# Supplementary material for: Molecular mechanism of Afadin substrate recruitment to the receptor phosphatase PTPRK via its pseudophosphatase domain
Source: eLife. 2022 Oct 20;11:e79855. doi: 10.7554/eLife.79855 (PMC9640194; doi:10.7554/eLife.79855)
Supplement: Figure 4—source data 4. [file elife-79855-fig4-data4.zip › Figure 4 – source data 4/Original files/Afadin CC-PTPRK-mutant pulldown 7.pdf]

Image ID: 0004599_11
Acquire Time: 22-Aug-2022 09:51:08

Acquisition Information

#	Image ID	Acquire Time	Channels	Resolution	Intensities	Quality	Analysis	Image Name	Comment
1	0004599_11	22-Aug-2022 09:51:08	700 800	169um	Auto Auto	high	Manual	0004599_11	

Image Display Values

Channel	Color	Minimum	Maximum	K
700	Gray Scale (Black on White)	2.95	41.4	0.49
800	Gray Scale (Black on White)	0.639	9.51	0

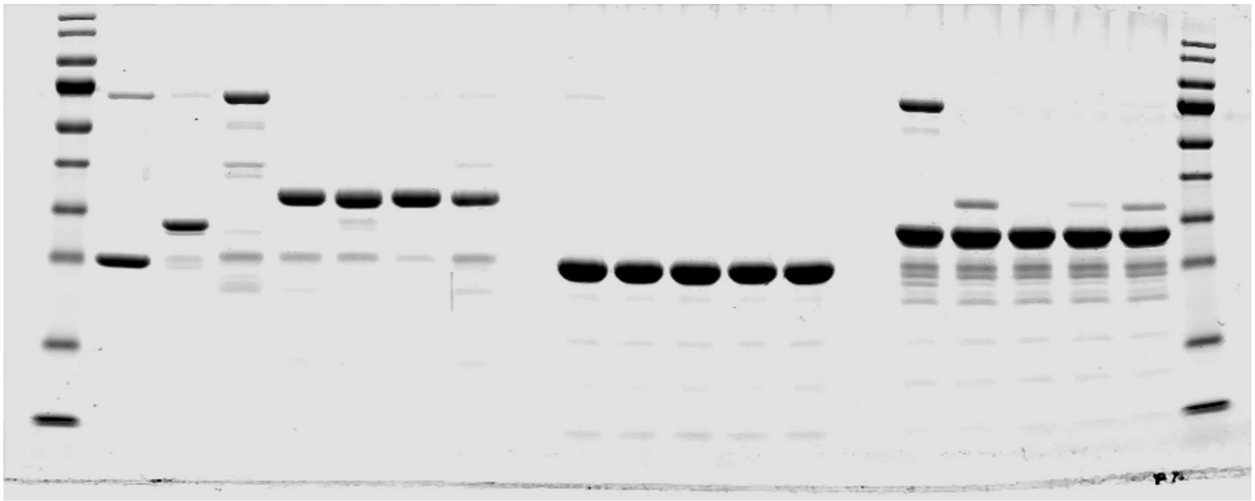

Acquisition Information (continued)

#	**Image Modifications**
1	Flip Top to Bottom Image ID: 0004599_01; Noise Removal Image ID: 0004599_02; Duplicate Image ID: 0004599_08; Sharpen Image ID: 0004599_1[illegible]
