# Supplementary figures and images for: Molecular mechanism of Afadin substrate recruitment to the receptor phosphatase PTPRK via its pseudophosphatase domain (part 2 of 2)
Source: eLife. 2022 Oct 20;11:e79855. doi: 10.7554/eLife.79855 (PMC9640194; doi:10.7554/eLife.79855)

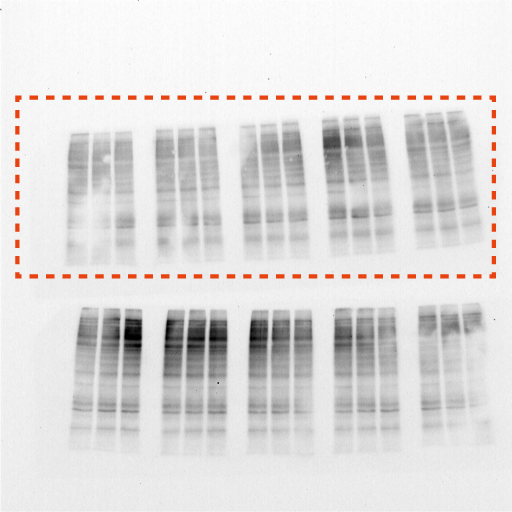

Supplement: Figure 5—source data 1. [file elife-79855-fig5-data1.zip › Figure 5 – source data 1/Original files/Figure 5 - source data 1 - highlighted/pTyr - highlighted.tif]

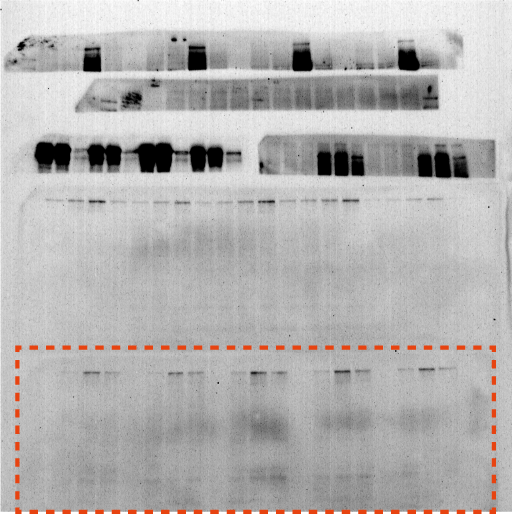

Supplement: Figure 5—source data 1. [file elife-79855-fig5-data1.zip › Figure 5 – source data 1/Original files/Figure 5 - source data 1 - highlighted/Total Afadin - highlighted.tif]

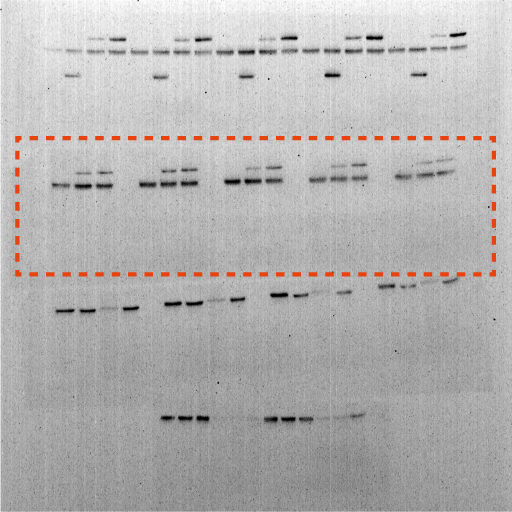

Supplement: Figure 5—source data 1. [file elife-79855-fig5-data1.zip › Figure 5 – source data 1/Original files/Figure 5 - source data 1 - highlighted/Tubulin - highlighted.tif]

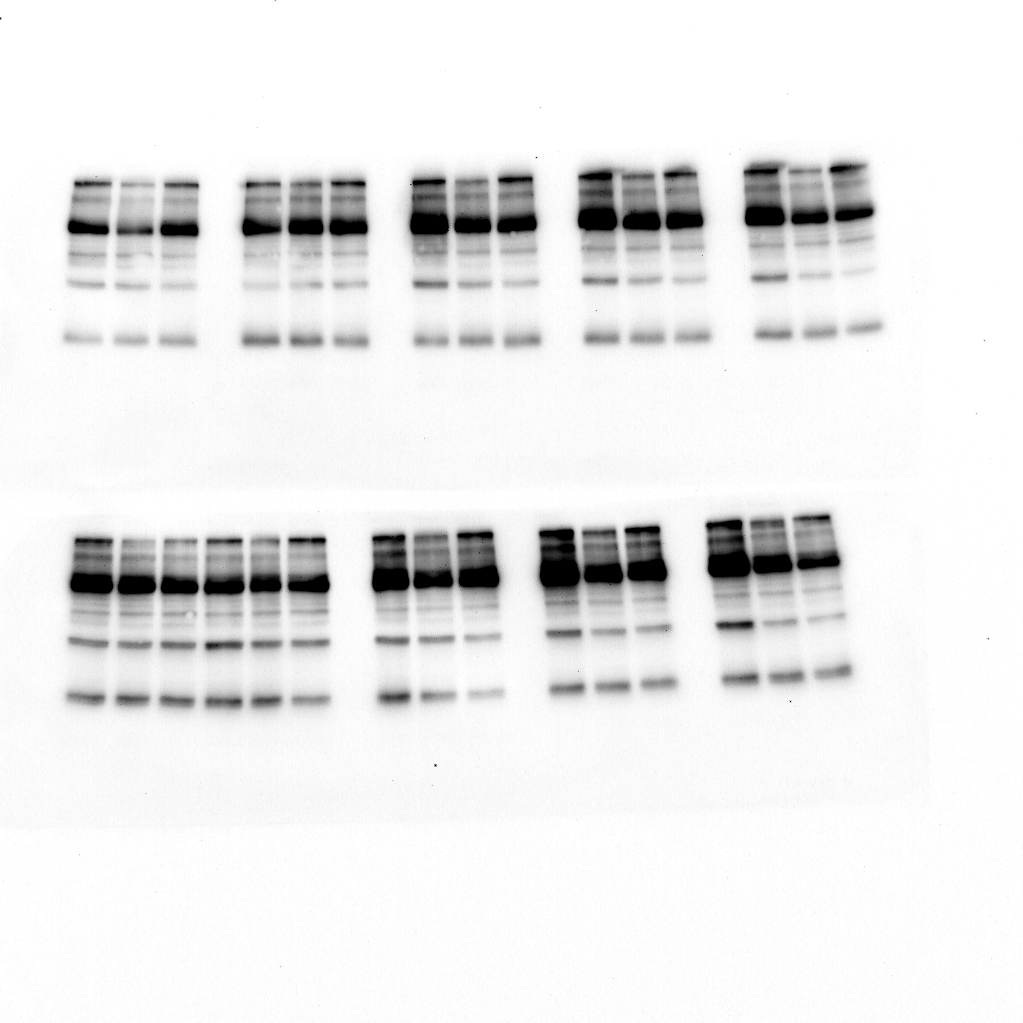

Supplement: Figure 5—source data 1. [file elife-79855-fig5-data1.zip › Figure 5 – source data 1/Original files/Figure 5 source data 1 - unmodified/Afadin pY1230 - top blot.tif]

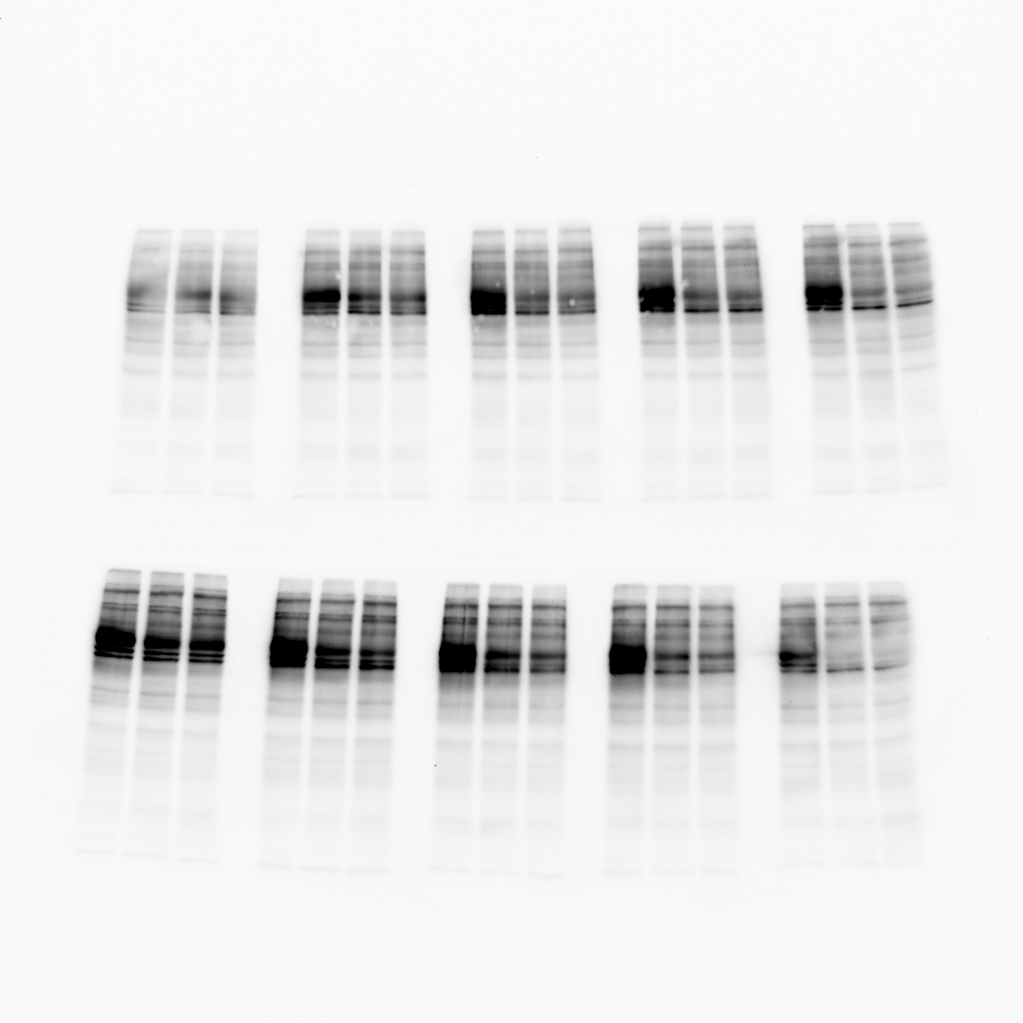

Supplement: Figure 5—source data 1. [file elife-79855-fig5-data1.zip › Figure 5 – source data 1/Original files/Figure 5 source data 1 - unmodified/p120 catenin pY228 - top blot.tif]

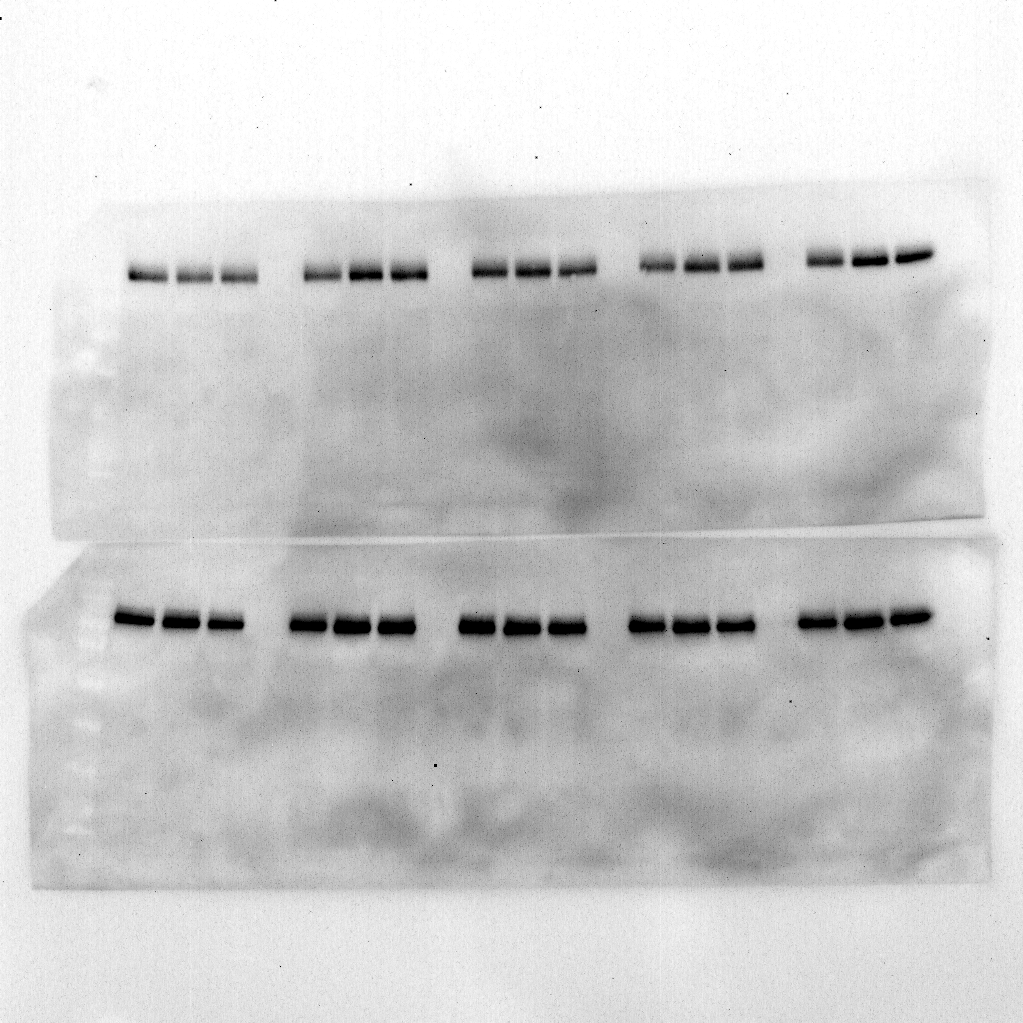

Supplement: Figure 5—source data 1. [file elife-79855-fig5-data1.zip › Figure 5 – source data 1/Original files/Figure 5 source data 1 - unmodified/p120 catenin total - top blot.tif]

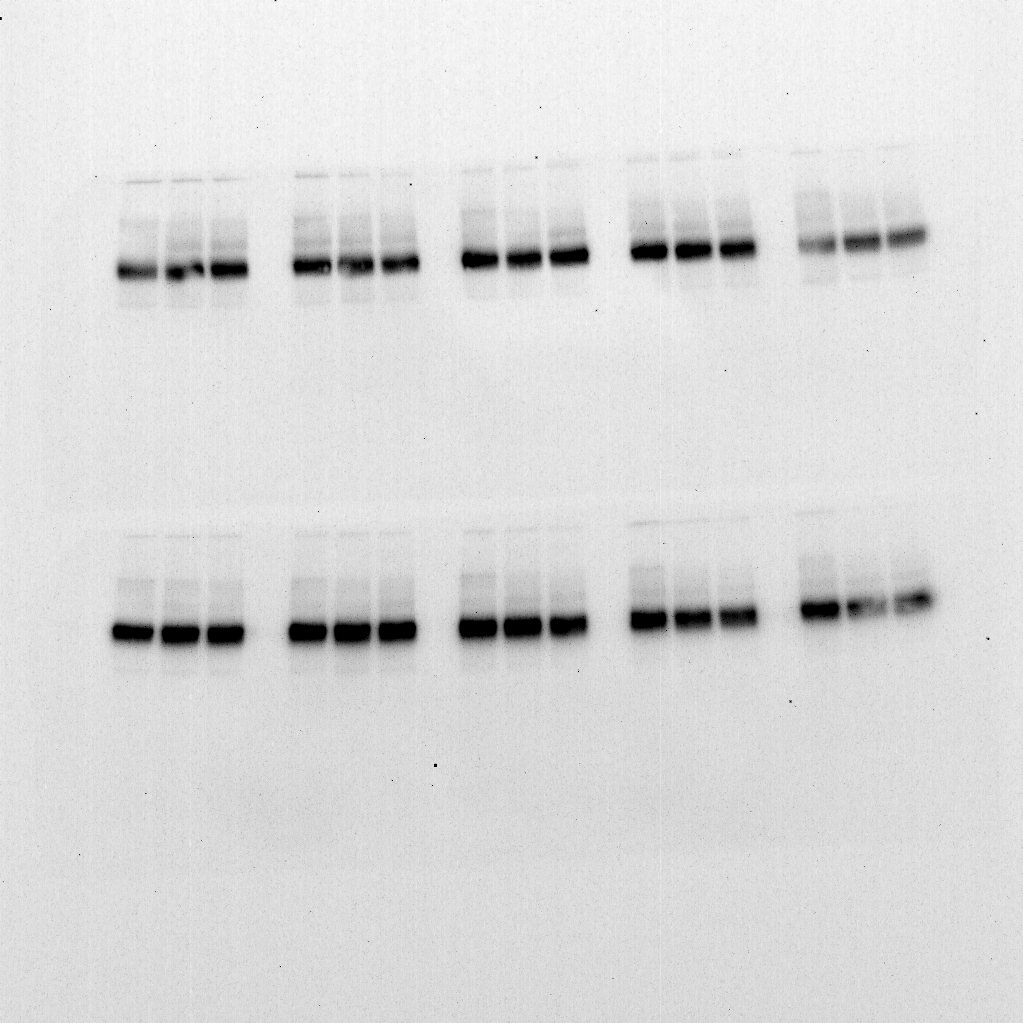

Supplement: Figure 5—source data 1. [file elife-79855-fig5-data1.zip › Figure 5 – source data 1/Original files/Figure 5 source data 1 - unmodified/Paxillin pY118 - top blot.tif]

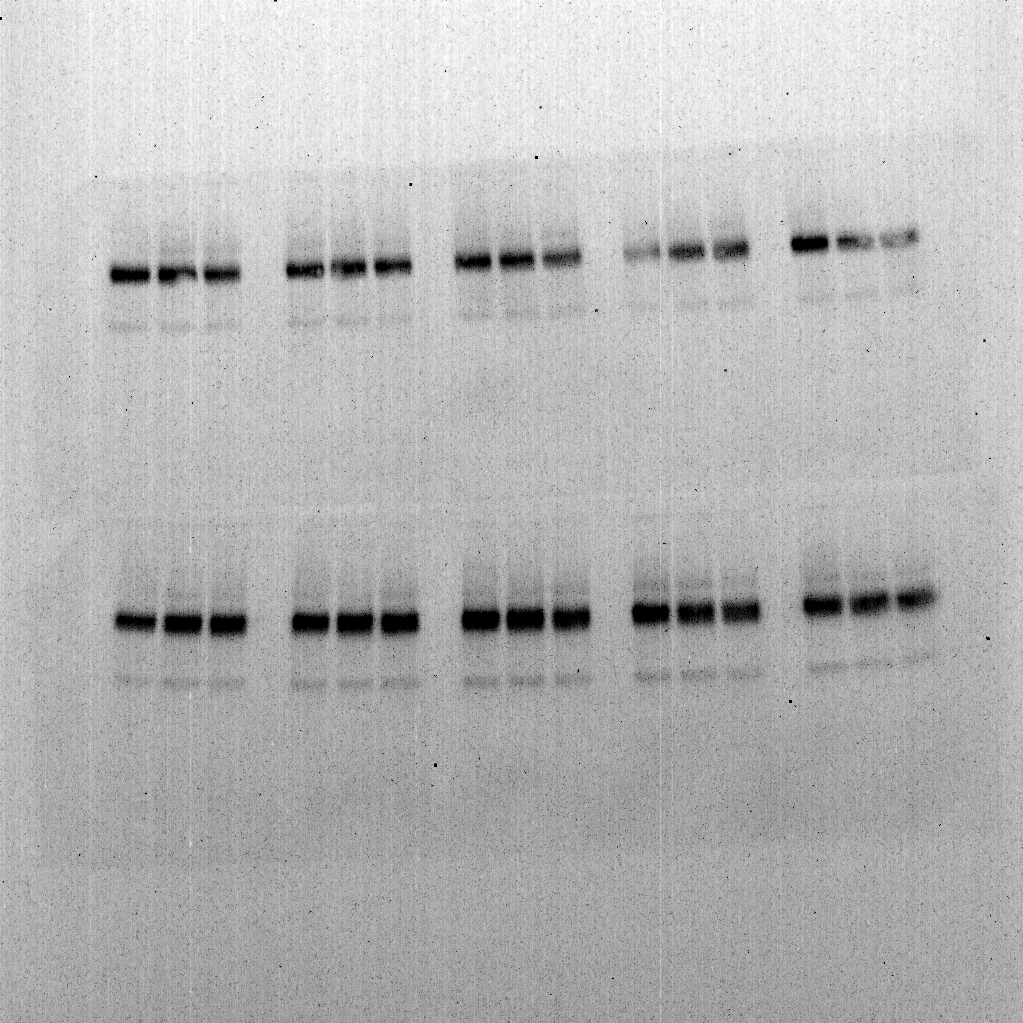

Supplement: Figure 5—source data 1. [file elife-79855-fig5-data1.zip › Figure 5 – source data 1/Original files/Figure 5 source data 1 - unmodified/Paxillin total - top blot.tif]

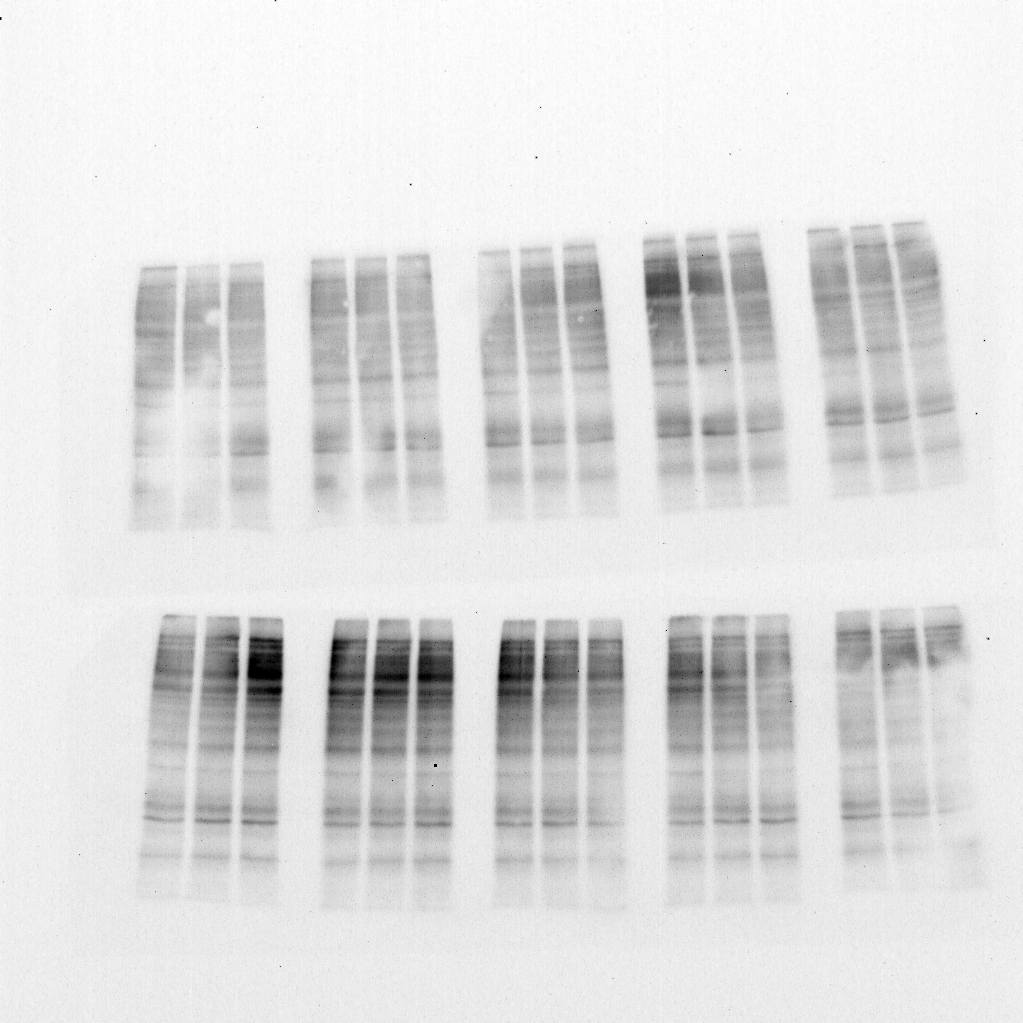

Supplement: Figure 5—source data 1. [file elife-79855-fig5-data1.zip › Figure 5 – source data 1/Original files/Figure 5 source data 1 - unmodified/pTyr - top blot.tif]

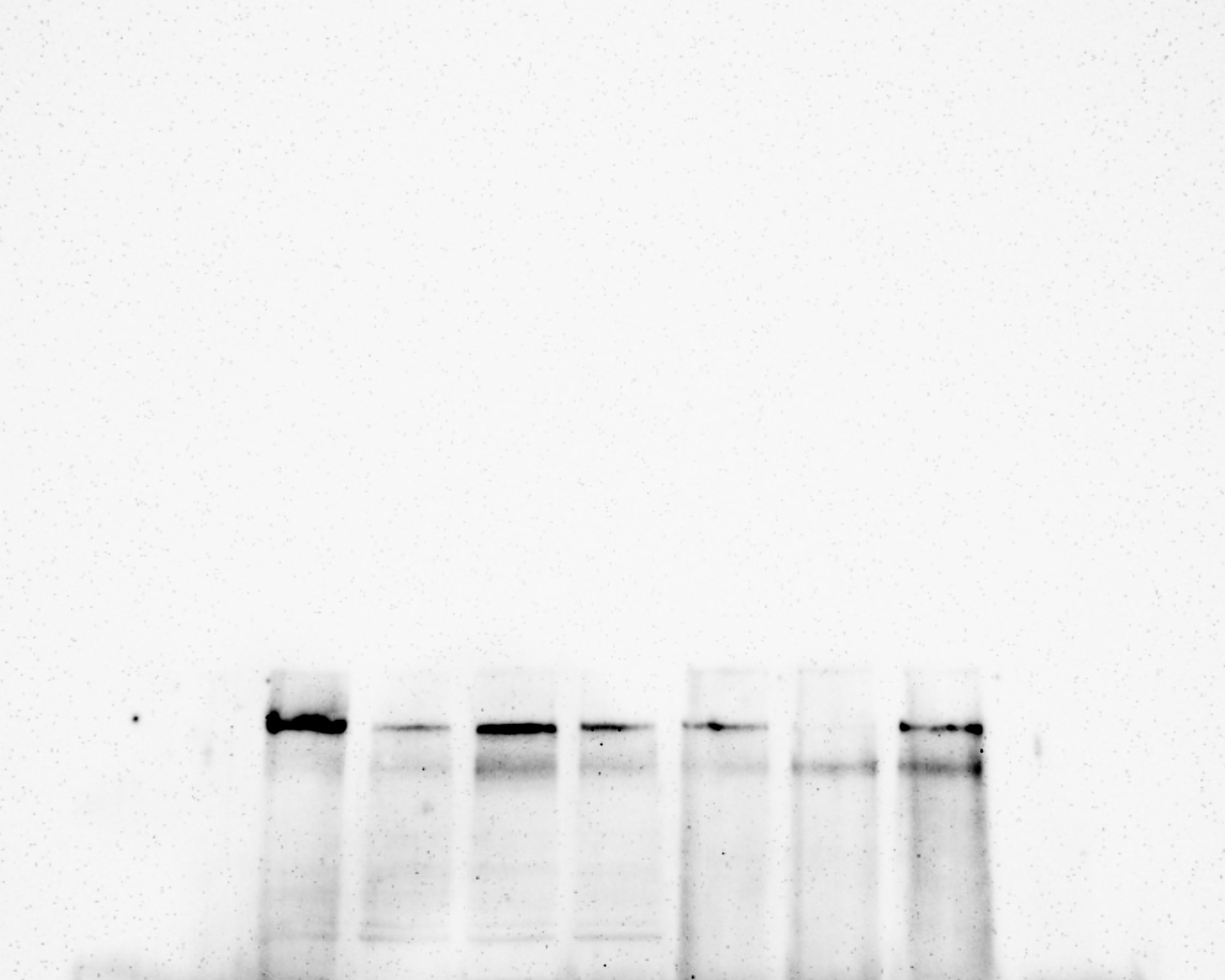

Supplement: Figure 5—source data 2. [file elife-79855-fig5-data2.zip › Figure 5 - source data 2/Original files/5C_Afadin.tif]

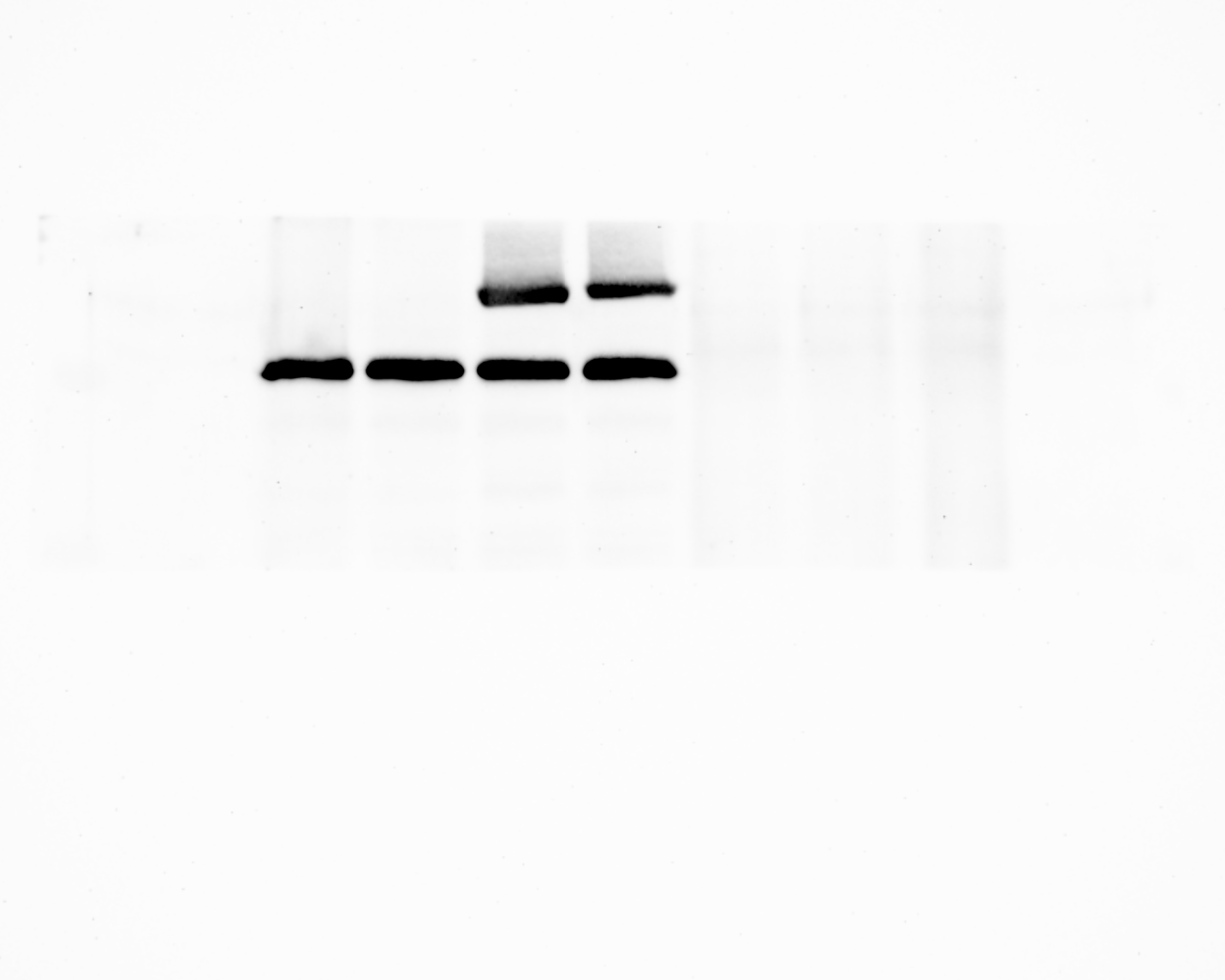

Supplement: Figure 5—source data 2. [file elife-79855-fig5-data2.zip › Figure 5 - source data 2/Original files/5C_Histag.tif]

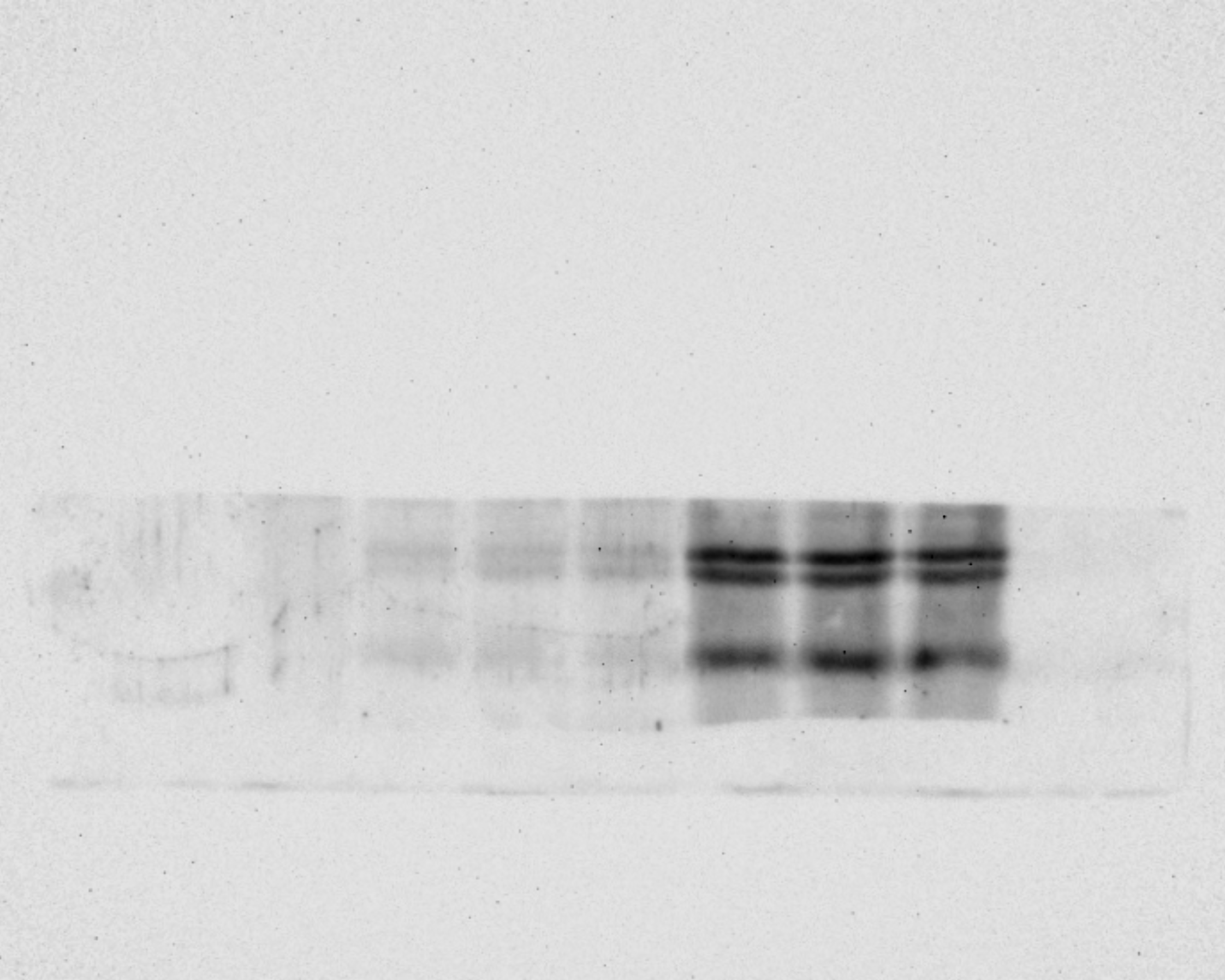

Supplement: Figure 5—source data 2. [file elife-79855-fig5-data2.zip › Figure 5 - source data 2/Original files/5C_IgG.tif]

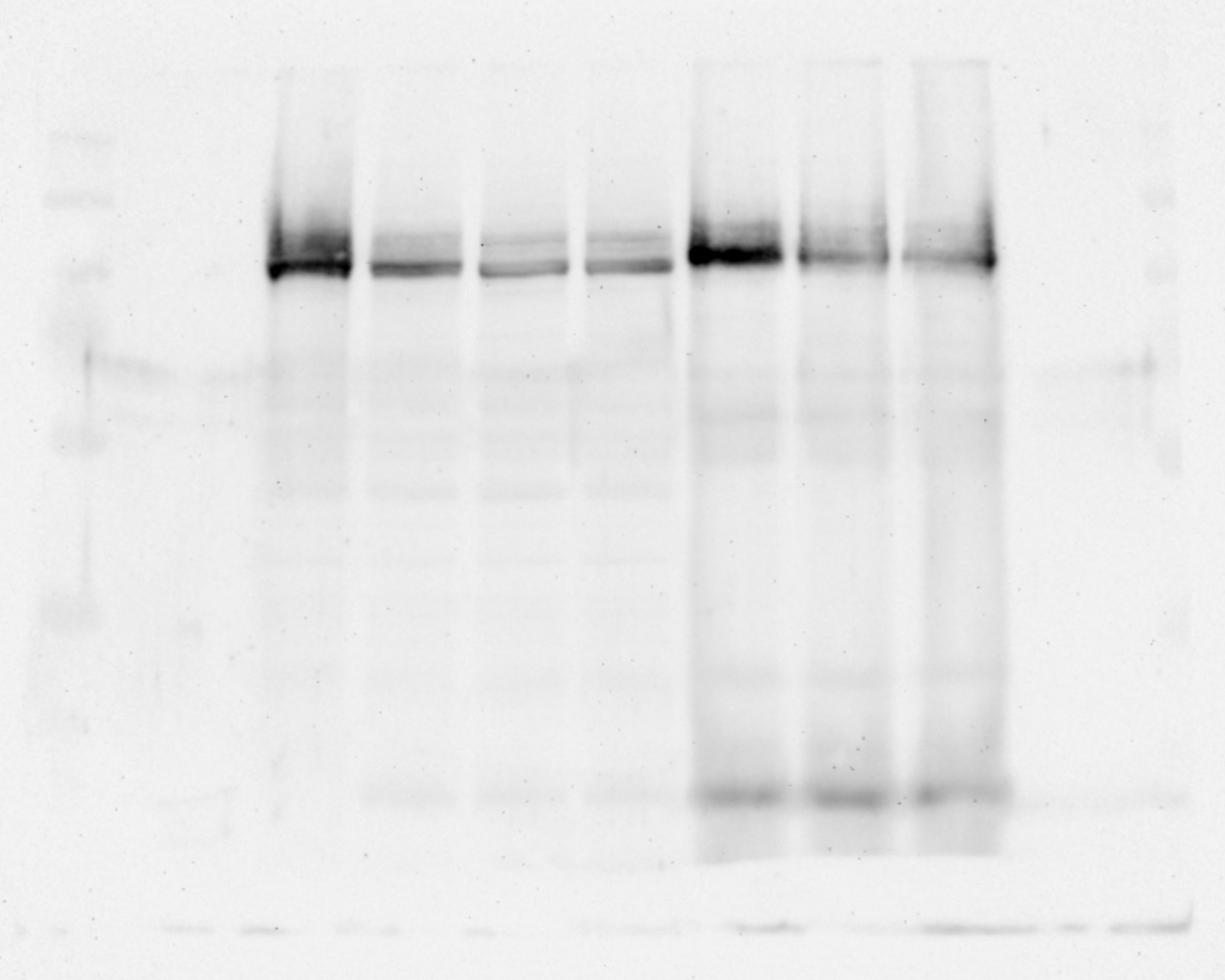

Supplement: Figure 5—source data 2. [file elife-79855-fig5-data2.zip › Figure 5 - source data 2/Original files/5C_p120.tif]

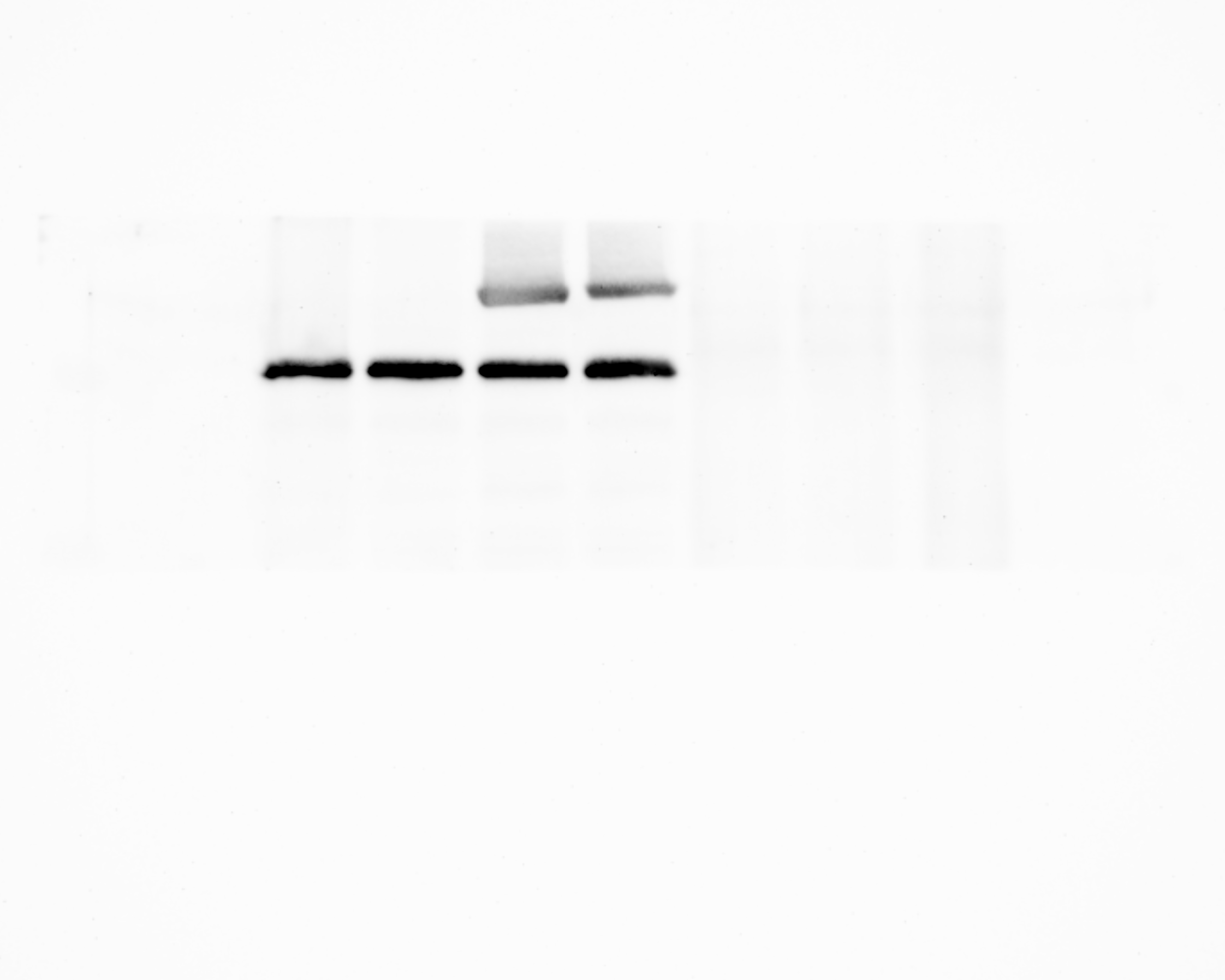

Supplement: Figure 5—source data 2. [file elife-79855-fig5-data2.zip › Figure 5 - source data 2/Original files/5C_Tubulin.tif]
